# Discovery of imidazole-based GSK-3**β** inhibitors for transdifferentiation of human mesenchymal stem cells to neurons: A potential single-molecule neurotherapeutic foresight

**DOI:** 10.3389/fnmol.2022.1002419

**Published:** 2022-12-15

**Authors:** Varsha Gupta, Tanushree Mahata, Rajsekhar Roy, Prabir Kumar Gharai, Aniket Jana, Shubham Garg, Surajit Ghosh

**Affiliations:** ^1^Organic and Medicinal Chemistry and Structural Biology and Bioinformatics Division, CSIR-Indian Institute of Chemical Biology, Kolkata, West Bengal, India; ^2^Department of Bioscience and Bioengineering, Indian Institute of Technology Jodhpur, Karwar, Rajasthan, India; ^3^Smart Healthcare, Interdisciplinary Research Platform, Indian Institute of Technology Jodhpur, Karwar, Rajasthan, India

**Keywords:** hMSC, transdifferentiation, GSK-3**β**, **β**-catenin, imidazole, **β**-III tubulin, neurodegeneration

## Abstract

The transdifferentiation of human mesenchymal stem cells (hMSC) to functional neurons is crucial for the development of future neuro-regenerative therapeutics. Currently, transdifferentiation of hMSCs to neurons requires a “*chemical cocktail*” along with neural growth factors. The role of the individual molecules present in a “chemical cocktail” is poorly understood and may cause unwanted toxicity or adverse effects. Toward, this goal, we have showcased the discovery of an imidazole-based “single-molecule” transdifferentiation initiator SG-145C. This discovery was achieved *via* screening of a small molecule library through extensive *in silico* studies to shortlist the best-fitting molecules. This discovery evolved through a careful selection to target Glycogen synthase kinase-3β (GSK-3β), which is one of the important proteins responsible for neurogenesis. Rigorous computational experiments, as well as extensive biological assays, confirmed that SG-145C has significant potential to transdifferentiate hMSCs to neurons. Interestingly, our results suggest that SG-145C can inhibit the proteasomal degradation of phosphorylated β-catenin, in turn promoting transdifferentiation of hMSCs into neurons *via* the Wnt pathway.

## Introduction

Neurodegenerative diseases such as Alzheimer’s disease (AD), Parkinson’s disease (PD), amyotrophic lateral sclerosis (ALS), and multiple system atrophy (MSA) cause neurological impairments, functional loss of neurons through progressive nerve cell deaths, and cognitive dysfunction. Despite rigorous investigations and continuous clinical attempts, only limited progress has been made in this direction, such as symptomatic treatments and preventive measures to improve the quality of life for the patients. Currently available medications are only effective to delay the degeneration of neuronal loss, which ranges from just months to years. Therefore, the discovery of an effective and potential therapeutic strategy is highly important to address these critical issues. Toward this goal, recently, exploration of stem cell-based therapy against neurodegenerative diseases has been attempted and research results are promising (Singh et al., 2016). Like in the case of PD, transplantation of neuronal stem cells has been shown to not only improve motor functions but also represses the disease progression to a good extent in the patients (Lindvall and Kokaia, 2010). MSC-derived NPCs have gained a lot of attention in recent years for the treatment of neurodegenerative diseases (Harris et al., 2012; Yang et al., 2012; Liu et al., 2014). In this line of work, exploration of Mesenchymal stem cells (MSCs) gain much attention. MSCs are multipotent stem cells, which have recently been explored against an extensive array of neurodegenerative diseases as a preventive as well as a curative measure. There are several advantages of using MSCs over other cells such as quick accessibility, non-invasive collection procedure, lower immunogenic potential, lack of graft versus host disease, and higher capacity of differentiation (Jung et al., 2009). Several reports have suggested that hMSCs are capable to enhance axonal growth when transplanted, in turn increasing motor functions and even promoting cognitive functions in *in-vivo* models (MacDonald et al., 2009). Moreover, studies show umbilical cord-derived MSCs can differentiate into neural cells and can even revive the damaged sections. Although stem cell therapy shows great potential to treat neurodegeneration, however, it has also some limitations such as lower rates of differentiation or otherwise uncontrollable differentiation in an *in vivo* environment (Salado-Manzano et al., 2020). Currently, transdifferentiation of hMSCs to functional neurons requires a mixture of multiple chemicals (chemical cocktail) along with neural growth factors (Divya et al., 2012; Rafieemehr et al., 2016; Cortés-Medina et al., 2019; Kruminis-Kaszkiel et al., 2020). Despite its great efficiency, this strategy creates complications like (i) difficulty in understanding the role of individual molecules in the cocktail, (ii) concern of toxicity, (iii) fate of individual molecules in downstream pathways involved, etc. Moreover, cells from different sources react differently to the cocktail (Sacchetti et al., 2016). Therefore, to avoid the complications related to this approach there is a need to develop an ideal strategy for “single-molecule” based transdifferentiation. In previous literature, it has been shown that activation of the Wnt pathway could promote the differentiation of neural precursor cells (NPCs) from embryonic stem cells (ESCs; Večeřa et al., 2020). Furthermore, the signaling pathway has always been associated with developmental stages of the hippocampal region of the cortex, supposedly because of its role in the proliferation and differentiation of NPCs (Gonçalves et al., 2016). Thus, targeting and activating the canonical Wnt pathway with a designed “single-molecule” that itself harbors the characteristics of other components of the cocktail would lead to a phenomenal outcome. GSK3 proteins have always been associated with neural development pathways including Wnt/β-catenin, Sonic Hedgehog, Notch, and growth factor/RTK. In the canonical Wnt/β-catenin pathway downstream protein GSK-3β (a type of serine/threonine-protein kinase) plays a crucial role in initiating the differentiation of neural progenitors (Doble and Woodgett, 2003; Bradley et al., 2012; Kim et al., 2014; McCubrey et al., 2016). Along with the other subtype GSK-3α, GSK-3β is expressed throughout the brain, thus any dysregulation can reciprocate into neurodegenerative disease progression (Urenjak et al., 1992; Klein and Melton, 1996; Emamian et al., 2004; Voeltz et al., 2006). In the Fibroblast growth factors (FGF) pathway GSK-3β activates c-myc’s proteasomal degradation by phosphorylating, thus inhibiting the activation of transcription factors responsible for neuronal differentiation (Hur and Zhou, 2010). In the NOTCH pathway GSK-3β binds to NOTCH 2 and phosphorylates it, thus inhibiting the transcriptional activation (Chen and Jiang, 2013). Moreover, in the sonic hedgehog pathway transcriptional factor Gli is activated or deactivated by the GSK-3β subtype only. The overactivation of GSK-3β mediated hyperphosphorylation will eventually lead to enhanced proteasomal degradation of β-catenin, ultimately reducing the differentiation factors such as TCF/LEF (Schön et al., 1998; Vignaux et al., 2020). Several other studies also have shown disruption in the neuronal links if GSK-3β has been knocked out. In case both the subtypes of GSK3 are knocked out, severe hyperproliferation was observed (Kimura et al., 2008). Therefore, it is prominent from these signaling pathways that GSK-3β has always been associated with developmental stages of neuronal development in one way or the other. Moreover, deleting the responsible genes in infants can cause improper migration and dendritic detriments of excitatory neurons (Clay et al., 2014). Altogether GSK-3β plays a very crucial role in adult neurogenesis and the study of Glycogen synthase kinase-3 signaling can play an important role in understanding neuropathological diseases (Jope and Roh, 2006; Almudena et al., 2013; Llorens-Martín et al., 2014; Lauretti et al., 2020).

Since biologically active therapeutics contain five-membered heterocycle ring imidazole, which acts on protein kinases and cell signaling molecules, we have made an effort to construct a library considering it as a core template. Numerous reports showed important heterocyclic compounds, which play crucial roles in inhibiting GSK3 but their clinical outcome is poor (Ding and Schultz, 2004; Brooks et al., 2009; Palomo et al., 2012; Thompson et al., 2019). Considering the above-multifaceted problem in this area, herein, we have carefully designed and developed an “imidazole-based small molecule library” that can anchor at the hydrophobic core of GSK-3β to deactivate its enzymatic action. *In silico* method was used to minimize the unnecessary usage of the chemical cocktail. The library was further screened using 3D-QSAR pharmacophore models. Next, depending on their drug-likeliness molecules were tested through ADMET studies from which SG-145C was found to be the most drug-like compound in the library of the molecule. Furthermore, we were also able to crystalize SG-145C and retrieve the ground-state molecular structure. Next, we performed density functional theory (DFT) with the SG-145C docked with GSK-3β to check the HOMO-LUMO interaction of ligand and protein. After performing different *in vitro* studies like immunocytochemistry, western blot, and RT-PCR we found an increase in several neuronal markers. Additionally, western blot showed that the molecules upregulate β-catenin and downregulate GSK-3β marking their prominence in directing the differentiation toward neuronal cell lineage. Therefore, this study is an attempt to produce a single molecule-based transdifferentiating factor aimed at converting MSCs into neurons by inhibiting GSK-3β activation, which alters the components of canonical Wnt signaling.

## Experimental section

### Materials

Human Wharton’s jelly mesenchymal stem cells (Himedia CL001-T25), mesenchymal stem cell expansion medium (Himedia AL512), antimycotic antibiotic solution (Himedia A002), fetal bovine serum (Invitrogen 2023-01-30), Penicillin Streptomycin solution (Invitrogen 15140-122), Trypsin EDTA Solution (Sigma T4174), Formaldehyde solution (Sigma-Aldrich 47608), Dimethyl Sulfoxide (Sigma Aldrich D2650), Bisbenzimide H33258 Flurochrome Trihydrochloride (Millipore Calbiochem, 38206), Triton X-100 detergent (SRL 9002-923-1), DPX Mountant (Sigma Aldrich 06522), Tween 20 detergent (Sigma Aldrich P9416), Glycine (Himedia MB013), Tris Base (Himedia MB029), Protease Inhibitor (Sigma Aldrich P8340), Phosphatase Inhibitor (Thermo-scientific 78428), MTT(3-[4,5-dimethylthiazol-2-yl]-2,5 diphenyl tetrazolium bromide) (Sigma Aldrich M2128), Neurobasal Medium (Invitrogen 21103049), RIPA lysis buffer (Thermoscientific Pierce 89901), 2-Mercaptoethanol (Millipore 63689), 2-Propanol (Millipore 59304), Protease inhibitor cocktail, Ammonium Persulfate (SRL 7727-54-0), TEMED (Sigma Aldrich T7024), Acrylamide/Bis-acrylamide solution [SRL 67394 (0124437)], Sodium Dodecyl Sulfate (Sigma Aldrich L3771), Bromophenol Blue (Sigma Aldrich 114391), Gylcerol (Sigma Aldrich G5516), Methanol (Supelco 494291), Trizol Reagent (Thermoscientific 1559608), Nuclease free water (Thermoscientific AM9932), SYBR GREEN PCR Master Mix (Invitrogen 4367659), Verso cDNA synthesis Kit (Thermoscientific AB1453A), Staining Buffer (BD Biosciences 554656), Fixation Buffer (BD Biosciences 554655), Kinase-Glo Luminescent Kinase Assays (Promega V6712), GSK-3β Recombinant (Merck 14-306), Adenosine 5′-triphosphate disodium salt hydrate (Sigma Aldrich 34369-07-8), Bovine Serum Albumin [SRL 85171 (01402990)], BCA Protein Assay KIT (Thermo Scientific 23227), Immobilon Western HRP Substrate (Millipore WBLU F0500), Brdu cell proliferation Assay Kit (Millipore Calbiochem QIA58), Anti Beta III Tubulin Antibody (Milli-pore MAB1637), Anti Alpha tubulin Antibody (Millipore 05-829), Anti NeuN Antibody (Abcam ab177487), Anti Map2 antibody (Thermofischer 131500), Anti Sox 9 antibody (Abcam ab185230), Anti GAP43 antibody (Millipore AB5220), Anti GFAP antibody (Abcam ab7260), Anti vimentin antibody (Abcam ab92547), Anti GSK-3β Antibody (Santa Cruz Biotechnology Sc 377213), Anti Beta Catenin antibody (Abcam ab16051), Anti phospho Akt antibody (Milli-pore 07-1643), FITC Mouse Anti-Human CD73 (BD Biosciences 561254), Goat Anti-Mouse IgG Fluorescein conjugate (Millipore 12-506), Goat Anti-Mouse IgG Antibody, FITC conjugate (Millipore AP 181F), Goat Anti-Mouse IgG Antibody, Cy3 conjugate (Millipore AP124C), Goat Anti-Rabbit IgG (H + L) Antibody FITC conjugate (Millipore 12-507), Goat Anti-Rabbit IgG antibody, Cy3 conjugate (Millipore AP 132C), HRP-anti Mouse (Millipore AP308P), HRP-anti Rabbit (Millipore AP-307P), 3color Pre-stained ladder (Puregene PG-PMT2922), CHIR99021 (Sigma SML 1046-5MG), NSC 668036 hydrate (Sigma SML 0046-5MG), CUR61414 (Sigma SML 0386-5MG), PVDF Membrane (Millipore), Anti-CD44 (EPR18668, ab189524), Anti-Calpain-1 (sc-390677), Anti Bcl_2_ (abclonalA0208), and Anti-Phospho-GSK-3β (Ser9) (5B3) CST 9323. Alkaline phosphatase-conjugated secondary antibodies [Sigma A5153 (Mouse) and Sigma A9919 (Rabbit)]. NBT-BCIP (Fermentus R0841, R821). Primers were purchased from GCC Biotech.

### Parameter generation and preparing QSAR sets

Avogadro software was used to convert the 2D chemical structures to 3D format. The crystal structure of GSK-3β (PDB ID: 1Q4L) was collected from RCSB Protein Data Bank (PDB) in PDB format and prepared in Discovery Studio v3.5 (Accelrys, United States, 2020) software. Maximum contribution structure (MCS) was followed to align the training and test set. Eventually, all the alignments were examined manually and the conformations with the lowest energy were used (Roy et al., 2018). The training set and test set were selected randomly keeping 80% in the training set (Supplementary Figures S1–S10). Several statistical analyses were performed to comprehend the QSAR model that showed a good correlation coefficient (*R*^2^) and cross-validation regression coefficient (*q*^2^; Supplementary Tables S1–S9).

### Molecular docking

For the docking studies, crystal structures of target proteins were collected from the RCSB database. The target proteins were prepared through the deletion of the present inhibitor and water molecules followed by merging all the polar hydrogen. Next, missing atoms, conforming missing loops, and standardizing the residues were performed. Upon completion, parameterization was performed using Merck molecular forcefield 94 (MMFF94) to determine the hydrophilic and hydrophobic interactions (Brooks et al., 2009; Zhang et al., 2012). Top 21 poses were considered and visualized in a 2D format that depicted the amino acid partner residues from the protein target sites. For further validation, the top 10 molecules were again specifically docked with the active site of GSK-3β.

### Synthetic scheme and procedure and scheme of the compounds

All the imidazole-based small molecules of our library for neuronal differentiation were synthesized and the compounds named SG-109C, SG-138C, SG-139C, SG-141C, SG-143C, SG-144C, SG-145C, SG-146C, SG-160C, and SG-162C were purified using RP-HPLC (Shimadzu) with Symmetry C-18 (Waters) (Supplementary Schemes S1, S2). All the molecules were characterized by ^1^H NMR, ^13^C NMR, and ESI-mass spectrometry (Supplementary Figures S11–S20). Detailed synthetic schemes, procedures, and single-crystal XRD data are elaborately described in the supplementary information (Supplementary Table S10).

### Single crystal X-ray diffraction study, MD simulation, and DFT with the crystal structure of SG-145C

Single colorless block crystals of SG-145C (0.24 × 0.22 × 0.15 mm^3^) were grown by slow evaporation from acetonitrile solvent. A suitable crystal was selected and mounted on a Bruker Kappa APEX-II CCD diffractometer with Cu Kα radiation. The crystal was kept at 101.0 K during data collection and the structure was solved by SHELXT and refined with SHELXL using the Olex2 program. CCDC data (deposition number 2132568) can be obtained free of charge from the Cambridge Crystallographic Data Centre *via* www.ccdc.cam.ac.uk/datarequest.cif. The crystal structure of the molecule was solved in the monoclinic crystal system with the P21/c space group. Next, the crystal data was placed through docking with the GSK-3β crystal structure retrieved from RCSB PDB (PDB ID: 1H8F), and the complex was subjected to molecular dynamics (MD) simulation. The simulation studies were performed in Biovia discovery studio client 2020 Discovery Studio v3.5 (Accelrys, United States, 2013) software. Unnecessary prebound ligands were removed and protein was fixed of any missing residue according to the generated protein report. Water was used as the solvation medium and the complex was put in CHARMM36 forcefield before solvating it with water. The forcefield helps in bringing the necessary restraints to escape the unnecessary surface characteristics. The next standard dynamic cascade was run keeping pressure (NPT), volume (NVT) and in the end minimizing the energy (NVE), in 1,000 steps of conjugate gradient minimization using the forcefield. The system was run for 20 nanoseconds (ns) and was heated from 50 K to 300 K throughout the process. The initial confirmation was taken as a reference to determine the RMSD of the entire complex. DFT calculations were done keeping time-dependent DFT (TDDFT) with the B3LYP function, resulting in Dmol3 properties of HOMO energy-LUMO energy and band gap energy.

### Cell culture

Mesenchymal stem cells isolated from umbilical cord blood were cultured in a mesenchymal stem cell expansion medium supplemented with 10% FBS (Invitrogen) and 1% antimycotic antibiotic and penicillin–streptomycin solution. Cells were maintained at 37°C and 5% CO_2_ using a humidified CO_2_ incubator (Thermofisher Scientific Heracell 150i). Trypsinisation was performed using 0.05% trypsin–EDTA after reaching 70%–80% confluency and harvested by centrifugation at 3000 rpm for 3 min.

### Cell viability assay

This assay was performed using MTT (3-[4,5-dimethylthiazol-2-yl]-2,5 diphenyl tetrazolium bromide) to check the viability of mesenchymal stem cells after treatment with imidazole-based small molecules. For this purpose, cells were seeded in a 96-well plate. After 24 h of plating, cells were treated with each small molecule at varied concentrations (3.125, 6.25, 12.5, 25, 50, 100 μM) and incubated for 24 h. Then, MTT solution (5 mg/ml) in filtered PBS was added to each well and incubated for another 4 h at 37°C. Post-incubation solution was discarded and 50 μl of 1:1 (v/v) DMSO: MeOH mixture was used to dissolve the purple formazan product formed by the reduction of MTT. A microplate reader (THERMO SCIENTIFIC; VARIOSKAN LUX) was used to measure the absorbance at 550 nm.

### Neuronal differentiation

A one-step induction protocol was used for the neuronal differentiation of hMSCs. For this, MSCs were cultured as mentioned above. After reaching 70%–80% confluency, cells were exposed to a neurobasal medium supplemented with 1% FBS containing different doses of small molecules and incubated for up to 7 days in the maintained environment to induce the differentiation of MSCs into neuron-like cells. The cells were checked at regular intervals to monitor the day-wise neuronal induction. Phase-contrast images were captured using Dewinter Microscope under 10× magnification.

### Immunocytochemistry

For this MSCs were cultured on 35 mm glass-bottom confocal dishes. After reaching sub-confluency cells were treated with different doses of small molecules and incubated for 7 days. Post-transdifferentiation, cells were fixed with 4% formaldehyde for 1 h at 37°C, washed twice with PBS, and permeabilized with 0.1% Triton X-100 containing 1% BSA in PBS for 15 min. Cells were again washed twice with PBS and were incubated with primary antibodies overnight at 4°C. After incubation, cells were washed twice with PBS and were incubated with FITC/Cy3 (Millipore) conjugated secondary antibodies. Nuclear staining was performed for 30 min using Hoechst 33258. Finally, after a PBS wash, images were captured using a fluorescence microscope (Olympus IX83) under 40X objective, Leica confocal microscope under 20X objectives, or Confocal Microscope Zeiss LSM 980 under 20X objective.

### Protein isolation and western blot analysis

For immunoblot analysis, proteins from transdifferentiated cells were lysed using RIPA lysis buffer containing a 1% protease inhibitor cocktail. The quantification of the proteins was performed using the BCA protein assay kit. Around 30 μg of protein was loaded in SDS-polyacrylamide gel and transferred onto PVDF (Polyvinylidene difluoride) membrane. Blocking was done with 5% BSA for 1 h at RT. After this probing was performed with the Primary antibodies: at 4°C overnight. After washing thrice with 1X TBST buffer, membranes were incubated with anti-mouse or anti-rabbit HRP-conjugated secondary antibody for 2 h at room temperature. Finally, after washing thrice with 1X TBST, the proteins were detected through either chemiluminescence using immobilon forte western HRP substrate or the Alkaline Phosphatase method using NBT-BCIP. The densitometric analysis was performed using ImageJ software where alpha-tubulin was used as a loading control.

### FACS analysis

For Facs Analysis, differentiated cells were trypsinized, and centrifuged and the pellet was collected in 15 ml tubes, fixed with 100 μl of fixation buffer for 10 min at RT. The fixative was removed by subsequent washing with 1X PBS twice. Permeabilization was performed using 0.1% Triton X for 5–10 min at RT followed by washing twice with 1X PBS. After this, cells were stained with fluorescence-tagged antibody using the stain buffer. For the staining of mesenchymal stem cells, FITC tagged CD73 antibody was used. Cells were incubated with the antibody for 1 h at 37°C. Finally, cells were washed using PBS, transferred in FACS tubes, and analyzed using a BD LSR-FORTESA flow cytometer.

### RNA extraction and reverse transcription-polymerase chain reaction

RNA isolation was performed using TRIzol reagent from mesenchymal stem cells after 7 days of treatment with SG-145C. The concentration of RNA and purity (A260/A280 > 1.8) was analyzed using Nanodrop. Total RNA isolated from differentiated cells was subjected to cDNA synthesis using Verso cDNA synthesis kit following the manufacturer’s instructions. The genes of interest were analyzed using SYBR green PCR master mix in an Applied Biosystems PCR machine. Primer designing was performed using a primer bank. The alpha-tubulin mRNA level was used as an internal normalization control. The amplification was performed using primer sequences provided in Table 1.

**Table 1:** Sequences of Primers used for RT-PCR.

Genes	Forward primer	Reverse primer
Alpha Tubulin	TCGATATTGAGCGTCCAACCT	CAAAGGCACGTTTGGCATACA
Beta III Tubulin	GGCCAAGGGTCACTACACG	GCAGTCGCAGTTTTCACACTC
Map2	GGATGGGCTTGTGTCTGATT	CTGGACCCACTCCACTCCACAAACT
Neuro D1	GGATGACGATCAAAAGCCCAA	GCGTCTTAGAATAGCAAGGCA
GFAP	CTGCGGCTCGATCAACTCA	TCCAGCGACTCAATCTTCCTC
Vimentin	GACGCCATCAACACCGAGTT	CTTTGTCGTTGGTTAGCTGGT
GSK-3β	AGACGCTCCCTGTGATTTATGT	CCGATGGCAGATTCCAAAGG
β-Catenin	CATCTACACAGTTTGATGCTGCT	GCAGTTTTGTCAGTTCAGGGA
Gap-43	GGCCGCAACCAAAATTCAGG	CGGCAGTAGTGGTGCCTTC
NF 200	GCAGTCCGAGGAGTGGTTC	CGCATAGCGTCTGTGTTCA
SOX 2	GCCGAGTGGAAACTTTTGTCG	GGCAGCGTGTACTTATCCTTCT
NSE	AGCCTCTACGGGCATCTATGA	TTCTCAGTCCCATCCAACTCC

### Kinase-Glo luminescent assay

This assay was performed using a black 96-well plate. In this particular assay 10 μl of SG-145C (Concentrations ranging from 3.125 to 100 μM) was added to each well. The serial dilution of the compound in the desired concentrations was prepared in Assay Buffer in advance. After this 10 μl (20 ng) of GSK-3β enzyme was added to each well followed by 20 μl of Assay Buffer containing 1 μM ATP and 25 μM substrate. After incubation for 30 min at 30°C, the enzymatic reaction was stopped using 40 μl of Kinase Glo Reagent. Then after 10 min of incubation luminescence was recorded using the Varioskan Lux multimode Microplate Reader. The IC50 values were calculated using origin software.

### BrdU cell proliferation assay

This cell proliferation assay was done using the Calbiochem BrdU cell proliferation assay kit (Millipore). For this, 100 μl of MSCs at 1–2 × 10^5^ cells/ml were seeded into a 96-well culture dish. After reaching 70%–80% confluency, cells were treated with SG-145C at Varied Concentrations from 3.125–100 μM and incubated at 37°C for 24 h. The cells without any treatment, i.e., undifferentiated MSC’s were used as a control set. Further steps were followed according to the manufacturer’s protocol. Finally, absorbance was measured using a Varioskan Lux multimode Microplate Reader at wavelength 450–595 nm.

### BrdU cell proliferation assay through immunocytochemistry

For this, cells were seeded in glass bottom confocal dishes and treated with SG-145C for 24 h. Cells without any treatment were used as a control set. Both control and treatment sets were labeled with a 10 μM BrdU labeling solution for 6 h. After that labeling, solution was removed and washed twice with PBS followed by fixation and permeabilization. DNA hydrolysis was done using 1 M HCl for 1 h at 37°C. HCl was removed and neutralized with 0.1 M Sodium Borate Buffer (pH 8.5) for 30 min at room temperature followed by PBS wash. After this anti-BrdU antibody was added and ICC protocol was followed as described above. Imaging was done using Confocal Microscope Zeiss LSM 980 under 20X objective.

### Live-dead cell assay

Live-dead cell assay was performed using Calcein AM and PI. Live cells were stained with Calcein AM (green) whereas dead cells were with Propidium Iodide (Red). In live cells, the nonfluorescent calcein AM is converted to a green-fluorescent calcein after acetoxymethyl ester hydrolysis by intracellular esterases. PI intercalates with the double-stranded nucleic acids of dead cells and imparts red fluorescence. For performing this assay, cells were differentiated using our molecule SG-145C while undifferentiated MSC’s were used as control. After 7 days of differentiation, cells were stained with 2 μϺ Calcein AM and 1 μϺ of PI for 30 min. After this, cells were washed thrice with 1× PBS and fluorescence imaging was done to assess the ratio of live and dead cells using a Fluorescence Microscope (Olympus IX83) under 40X.

### Statistical analysis

Statistical analysis was performed using Graph-Pad Prism Software version 8. One-way ANOVA or Student’s Unpaired 2-tailed *t*-test was used to calculate statistically significant differences between control and treatment groups. Data represent the mean ± Standard error. Value of *p* < 0.05 was set as the threshold for statistical significance between the control and treatment groups.

### Electrophysiology

The hMSCs were grown and differentiated into neurons on 18 mm coverslips placed in 24 well plates. On day 7 the SG-145C-treated MSCs were placed on the recording stage of the recording microscope. Next, the media was replaced with aCSF (artificial cerebrospinal fluid). The differentiated cells were seen and patched using an Olympus BX-83 microscope. Cells were patched in cell-attached configuration with gigaseal of 3–6 GΩ. The holding potential was set to −60 mV. Electrical stimulation was performed using a field electrode which was placed adjacent to the coverslip, the voltage gap was kept at a minimum and the recording was done using multi clamp 700B software. Sweep steps were kept at 10 steps. Episodic impulse was given at every 50 ms.

## Results

### Construction of imidazole-based small molecule library and screening through the 3D-QSAR model

**Figure 1:**
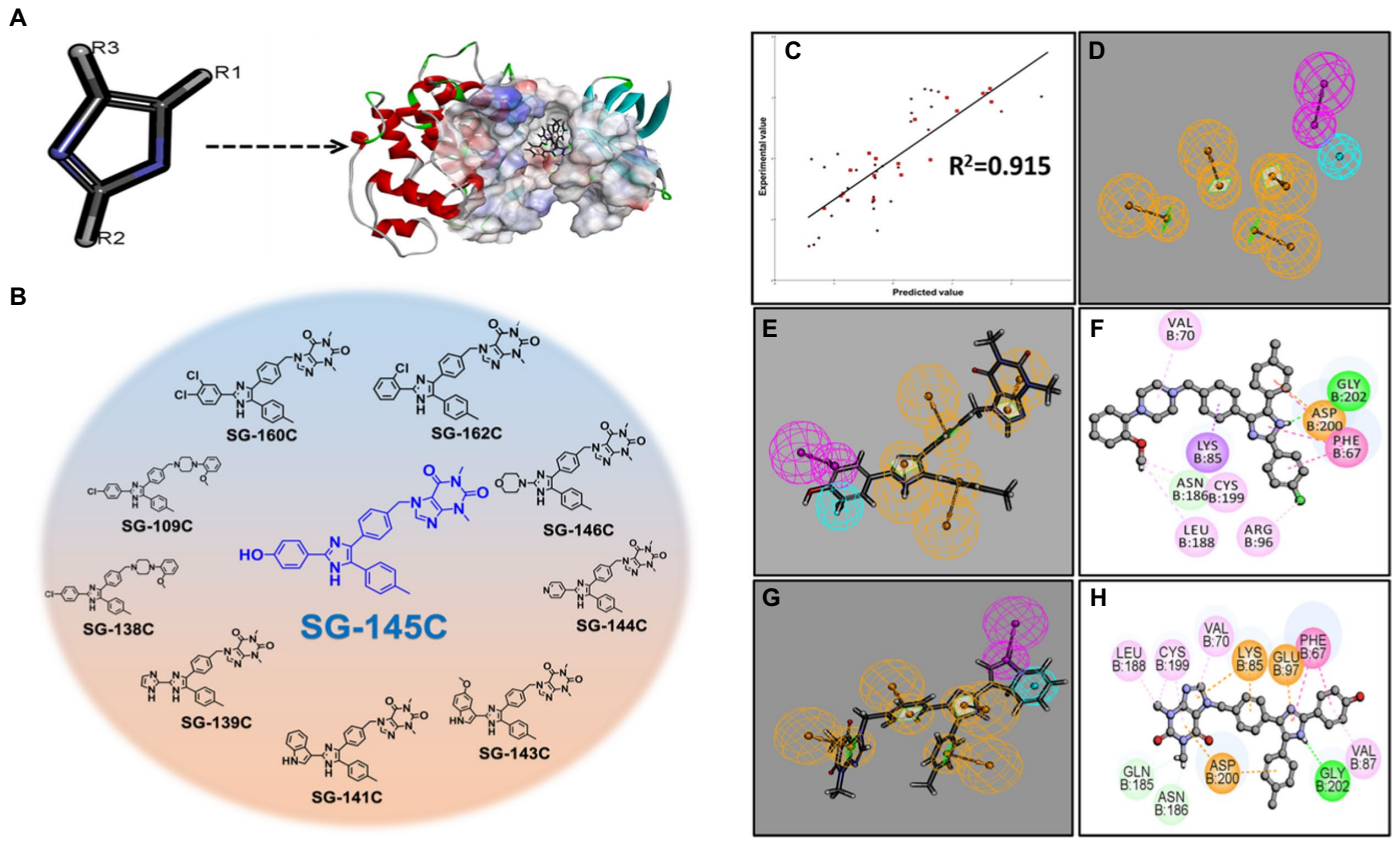
Establishment of the pharmacophoric model with GSK-3β and screening of lead molecules. **(A)** Projecting imidazole moiety into the GSK-3β pocket to modulate the scaffold at defined positions. **(B)** Among the top 10 screened molecule SG-145C stands out to be the best. **(C)** 3D-Pharmacophoric model showing a correlation coefficient of 0.915. **(D)** 3D coordinates of ligands with respect to pharmacophoric interactions **(E,F)** Pharmacophore mapping with the 3D pharmacophore model depicting SG-145C to be the best-fitted molecule. **(G,H)** Pharmacophore mapping with the 3D pharmacophore model depicting SG-138C to be the 2nd best-fitted molecule.

First, imidazole was placed in the active pocket of GSK-3β and, it was subjected to substitution with various key pharmacophores using the “Grow scaffold” module of Biovia Discovery Studio v3.5 (Accelrys, United States, 2020; Figures 1A,B). The projection was performed solely on the basis to find the best-fitted molecule with the chemical reactions that could be possible. Next, the highest-scoring molecules were then modified accordingly and screened through a 3D QSAR model that screened down the number of molecules in the library from 162 to 50 molecules. Next, these 50 molecules were further inspected by molecular docking and SG-145C showed the best docking score among all 50 molecules (Figures 1C,D). This procedure further screened down the number of molecules to 21. Further, ADMET studies were performed to analyze the drug likeliness and finally, we shortlisted a library of potential 10 molecules and decided to explore them experimentally.

### Screening of molecules using molecular docking studies

Discovery Studio v3.5 (Accelrys, United States, 2020) was used to perform the protein and ligand preparation. Libdock program of Discovery studio was used to determine the binding site poses that are biologically active within the active site. CHARMM36 force-field was used for the energy minimization within the polar and nonpolar components (Palomo et al., 2012). CAESAR (Conformer Algorithm based on Energy Screening and Recursive build-up) method was used for the generation of conformations keeping every other component as default ultimately to specify the structural interactions depicted in a 2D diagram of the result (Figures 1E–H). The top 10 molecules were analyzed in 3D coordinates under CHARMM36 force-field docking and the results were analyzed (Supplementary Figure S30). Analysis of the results showed the binding partners for both the receptors and the calculated LibDock score depicts SG-145C to be the best therapeutic lead for further screenings.

### Cytocompatibility of small molecules

**Figure 2:**
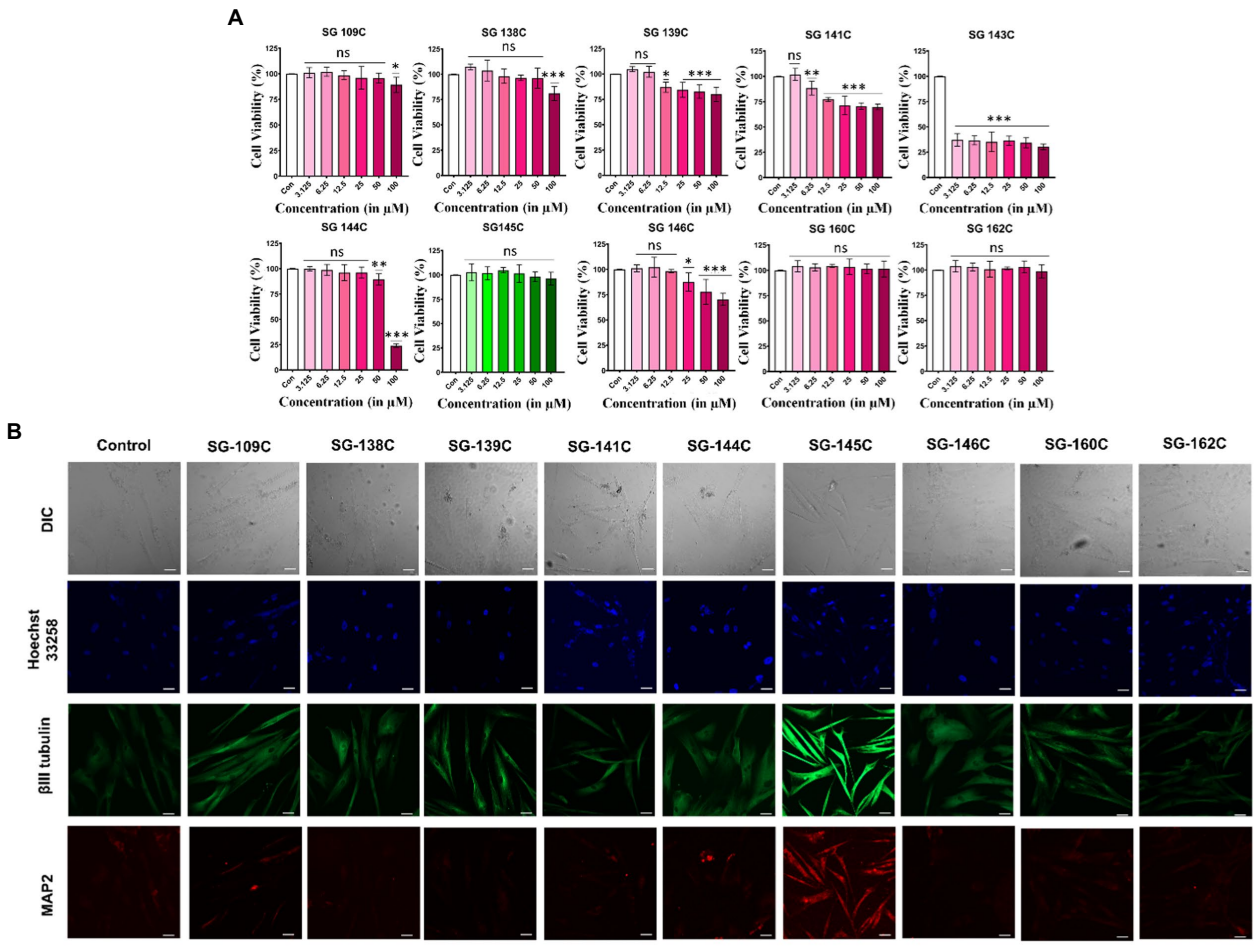
MTT and immunocytochemistry assay for identification of SG-145C as a lead molecule. **(A)** Percentage of cell survival after the treatment with the molecules in an increasing gradient of doses (3.125–100 μM) for 24 h. *n* ≥ 3 in all data sets and significance was calculated using one-way Anova. **(B)** MSCs were treated with the small molecules for 7 days and neuron specific markers were observed using β-III Tubulin (AF-488) and Map2 (AF-543) *via* immunocytochemistry. Images were captured in 20× magnification where the scale bar corresponds to 70 μm. Significance was calculated using students unpaired *t*-test *p < 0.05, **p < 0.01, ***p < 0.001.

The cytocompatibility of the molecules was assessed through MTT (3-(4,5-dimethylthiazol-2-yl)-2,5-diphenyltetrazolium Bromide) assay after the treatment with each molecule in increasing doses (3.125–100 μM) for 24 h. As shown in (Figure 2A), SG-145C, SG-109C, SG-162C, and SG-160C were found to be completely non-cytotoxic even at higher doses whereas SG-138C, SG-139C, SG-141C, SG-144C, and SG-146C were slightly cytotoxic in higher doses. SG-143C was cytotoxic across all tested doses indicating its inaptness for our study.

### Initial screening of molecules through immunocytochemistry

The ability of the shortlisted molecules to induce trans-differentiation in MSCs was analyzed by the treatment of 5 μM of molecules in MSCs for 7 days. Morphological changes were evident by neurite-like extensions in the treatment sets as compared to the control one (undifferentiated MSC’s kept for 7 days in Neurobasal media supplemented with 1% FBS in absence of SG-145C). Differentiated cells were stained with neuron-specific marker β-III Tubulin and somatodendritic marker Map2. The expression of neuronal protein β-III Tubulin was highest in the SG-145C treatment set compared to other molecules while Map2 staining was only observed in SG-145C treatment (Figure 2B). Therefore, using neuronal morphology (dendrite like extensions) and expression of somatodendritic marker Map2 as the attributes of neural trans-differentiation, SG-145C was selected as the lead candidate for further validation studies. This assay validated our *in silico* modeling prediction and we proceeded with SG-145C for understanding the detailed mechanism of transdifferentiation.

### Dose and time-dependent study of lead molecule SG-145C on transdifferentiation

**Figure 3:**
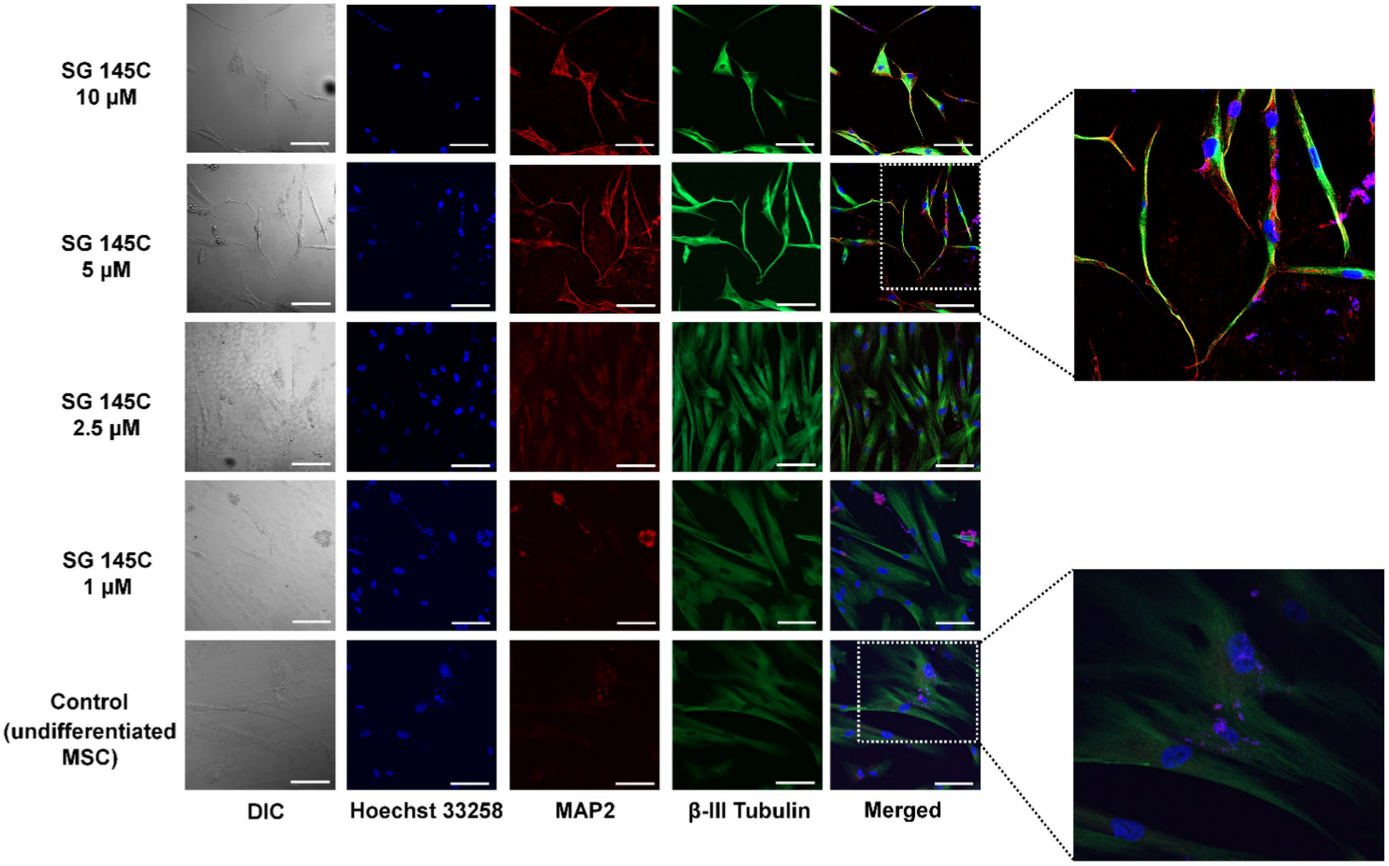
Evaluation of optimum dose of SG-145C for transdifferentiation. An increasing gradient of SG-145C was used to treat MSCs and immunofluorescence study of neuronal markers β-III Tubulin (AF-488) and Map2 (AF-543) performed to elucidate the optimal concentration. Images were captured in 20× magnification where the scale bar corresponds to 70 μm.

To optimize the time and dose of the lead molecule required for transdifferentiation, SG-145C was added in different concentrations (1, 2.5, 5, and 10 μϺ) after cells reached sub-confluency. Cells were monitored for morphological changes in a day-wise manner. Prominent changes in the morphology were observed from the 3rd day onwards in 5 and 10 μM treatment sets whereas no changes were seen in control and low concentration sets. Cells started exhibiting processes and prominent dendrite-like extensions till the 7th day of treatment (Supplementary Figure S21). The cells were fixed, permeabilized, and stained with neuron-specific antibodies Beta III Tubulin and Map2 which is also a somatodendritic marker. There was not any significant morphological and marker expression difference between the 5 and 10 μM doses, so we proceeded with the lower dose. By looking at the cellular morphology and expression of neuronal proteins, the optimum concentration of the molecule to show transdifferentiation was found to be 5 μM for 7 days (Figure 3).

### Characterization of transdifferentiated neurons

**Figure 4:**
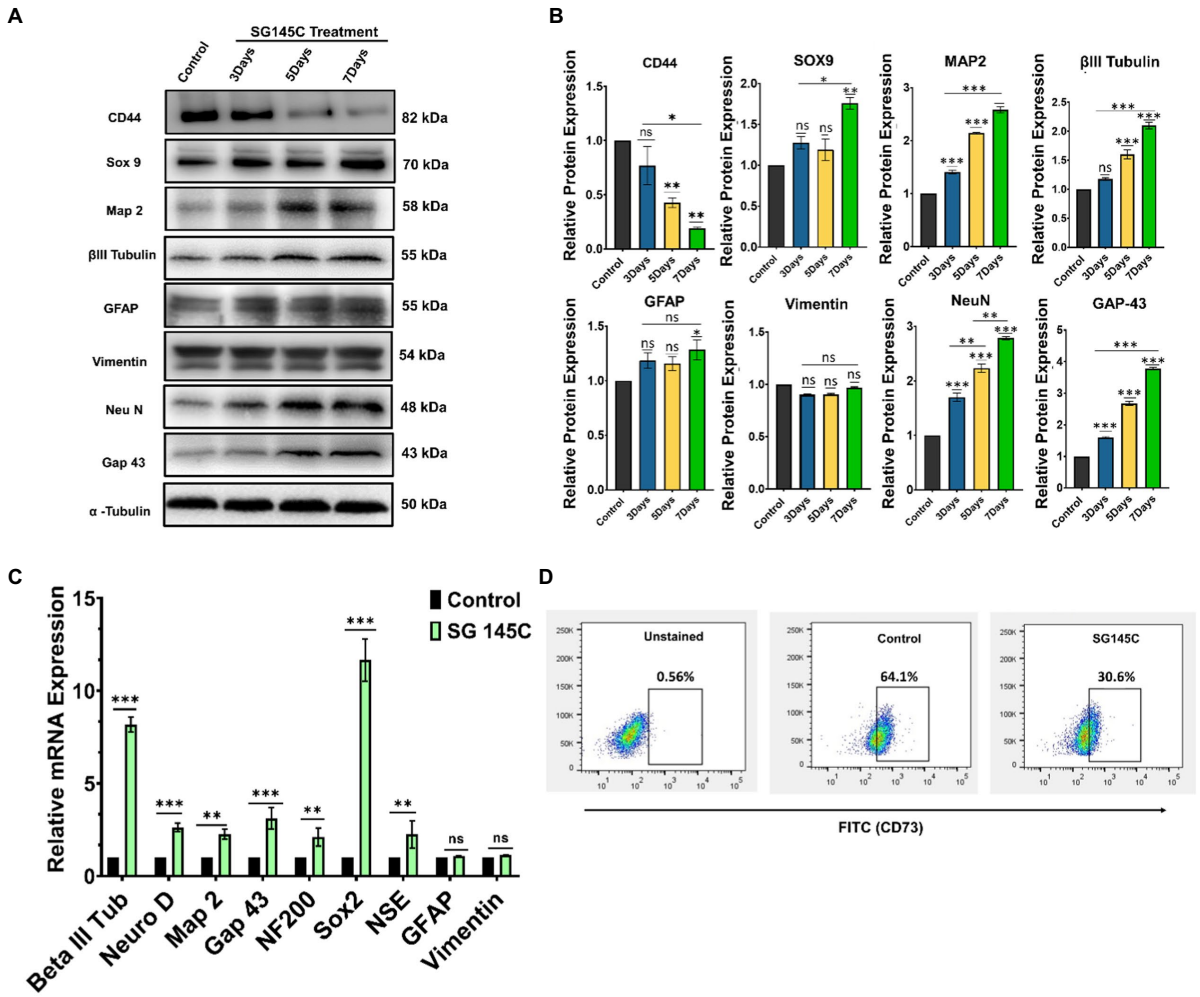
Characterization of the differentiated neurons. **(A)**. Immunoblot analysis to check the expression of neuronal marker proteins at various time points after treatment with 5 μM SG-145C followed by its **(B)** densitometric analysis. **(C)** qPCR analysis reveals the expression pattern of the neuronal marker genes in the transdifferentiated neurons after the treatment with SG-145C. **(D)** FACS analysis reveals the reduction in mesenchymal stem cell marker CD73 after differentiation. Significance was calculated using one-way Anova for Western Blots and student’s unpaired *t*-test for qPCR. *p < 0.05; **p < 0.01; ***p < 0.001.

To validate that mesenchymal stem cells differentiate into neuronal lineage after treatment with 5 μM SG-145C, the levels of neuron-specific proteins β-III Tubulin, Map-2, Neu N, Gap 43, and Sox9 were examined through western blotting in which undifferentiated MSC’s was used as a control set. The expression of these proteins increased gradually in a day-wise manner as compared to the control set and this gradual increase in the expression of neuronal proteins upon treatment with SG-145C (upto 7 days) reveals the time-dependent nature of the process. Moreover, there was no increase in the levels of astrocytic marker GFAP. In addition to this, the Mesenchymal marker CD44 was observed to be decreased in a day-wise manner, showing the gradual loss of Mesenchymal traits (Figures 4A,B). As the differentiation progresses, the stem-cell-like properties of MSCs reduce, which we have shown through the evaluation of CD73 population through FACS analysis. The differentiation into neuronal lineage was associated with a reduction in the mesenchymal stem cell marker CD73 (Figure 4D). RT-PCR data also reflected the elevated expression of neuronal genes such as β-III Tubulin, Map-2, Neu N, Gap 43, Sox2, NF200, and NSE after the treatment with SG-145C. There was no elevation in the non-neuronal genes such as GFAP and Vimentin proving MSCs were only transdifferentiated into neurogenic cells (Figure 4C). To check the expression pattern of neuronal proteins in the control set that is 7 days incubation of Mesenchymal stem cells without SG-145C treatment, a western blot was performed in a day-wise manner. The protein levels showed no changes revealing the fact that the neuronal differentiation was induced because of our drug treatment (Supplementary Figure S25).

### Effect of SG-145C on cell proliferation and cell survival

**Figure 5:**
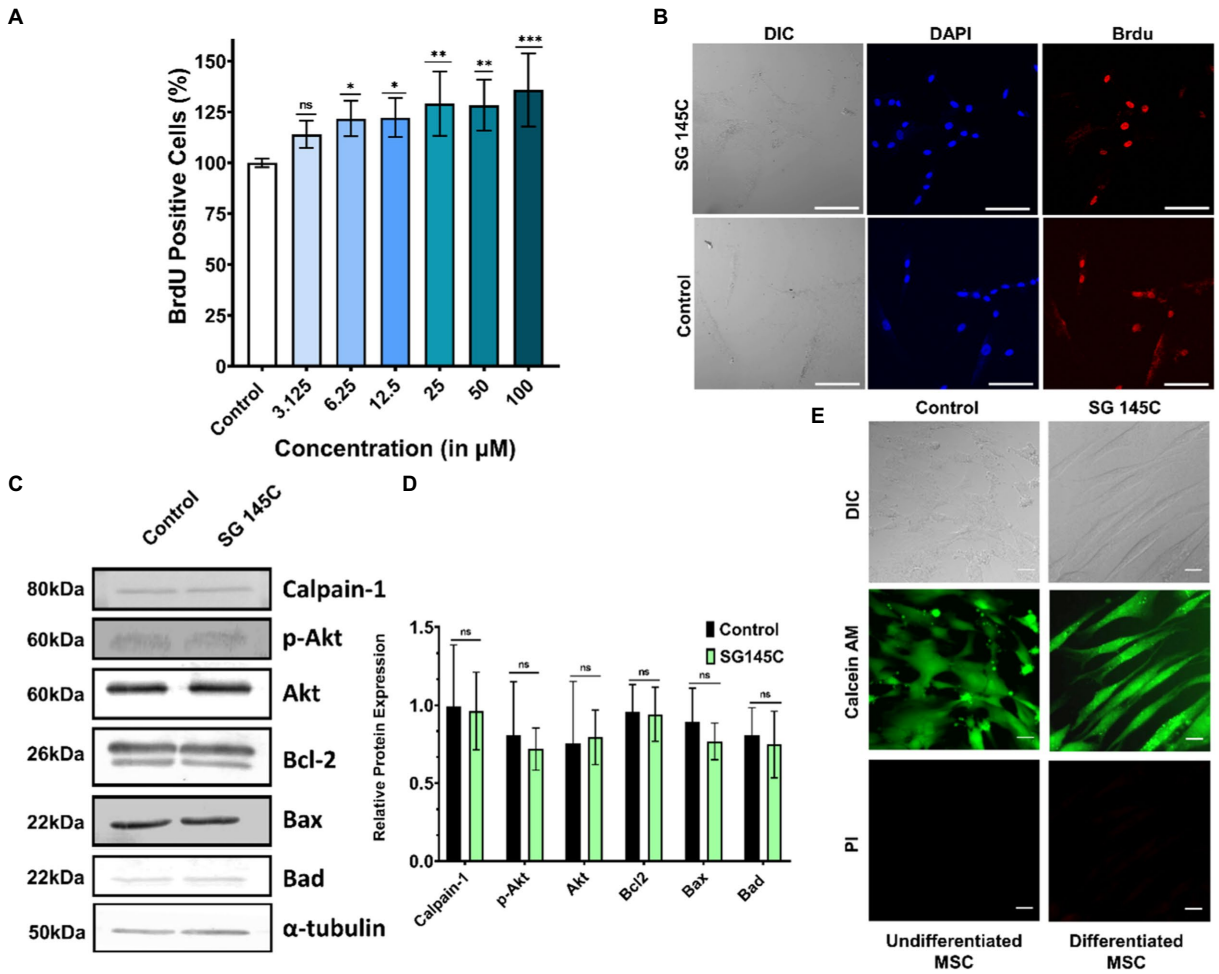
Cell proliferation and survival. **(A)** BrdU proliferation assay to assess the actively proliferating cells after the treatment of SG-145C in a dose-dependent manner. *n* > 3 in all data sets and significance was calculated using one-way Anova. **(B)** Immunocytochemistry to check the BrdU positive cells. Images were captured in 20X magnification where the scale bar corresponds to 70 μm. **(C)** Immunoblots of apoptotic and survival genes to check the homeostasis status of differentiated neurons followed by its **(D)** densitometric analysis. **(E)** Live-Dead cell assay using Calcein AM and PI. Images were captured in 40X magnification where the scale bar corresponds to 20 μm. *p < 0.05; **p < 0.01; ***p < 0.001.

Brdu Assay revealed there is no effect in the population of proliferating cells after the treatment with SG-145C for 24 h (Figure 5A) as compared to Control (undifferentiated MSC’s). ICC data also showed a significant number of proliferating cells identified by BrdU staining after the treatment which is comparable to that of control (Figure 5B). Cell homeostasis was verified using immunoblots of apoptotic markers Bad, Bax, Bcl2, and Calpain-1, which demonstrated no significant changes after 7 days of treatment. There was also no increase in alternate autophagy pathways as we observed unchanged Akt and P-Akt levels (Figures 5C,D). A live-dead assay was also performed to assess the cytocompatibility of SG-145C, which reveals that all the cells are viable and intact after the treatment (Figure 5E).

### Evaluation of MD simulation by DFT calculations

**Figure 6:**
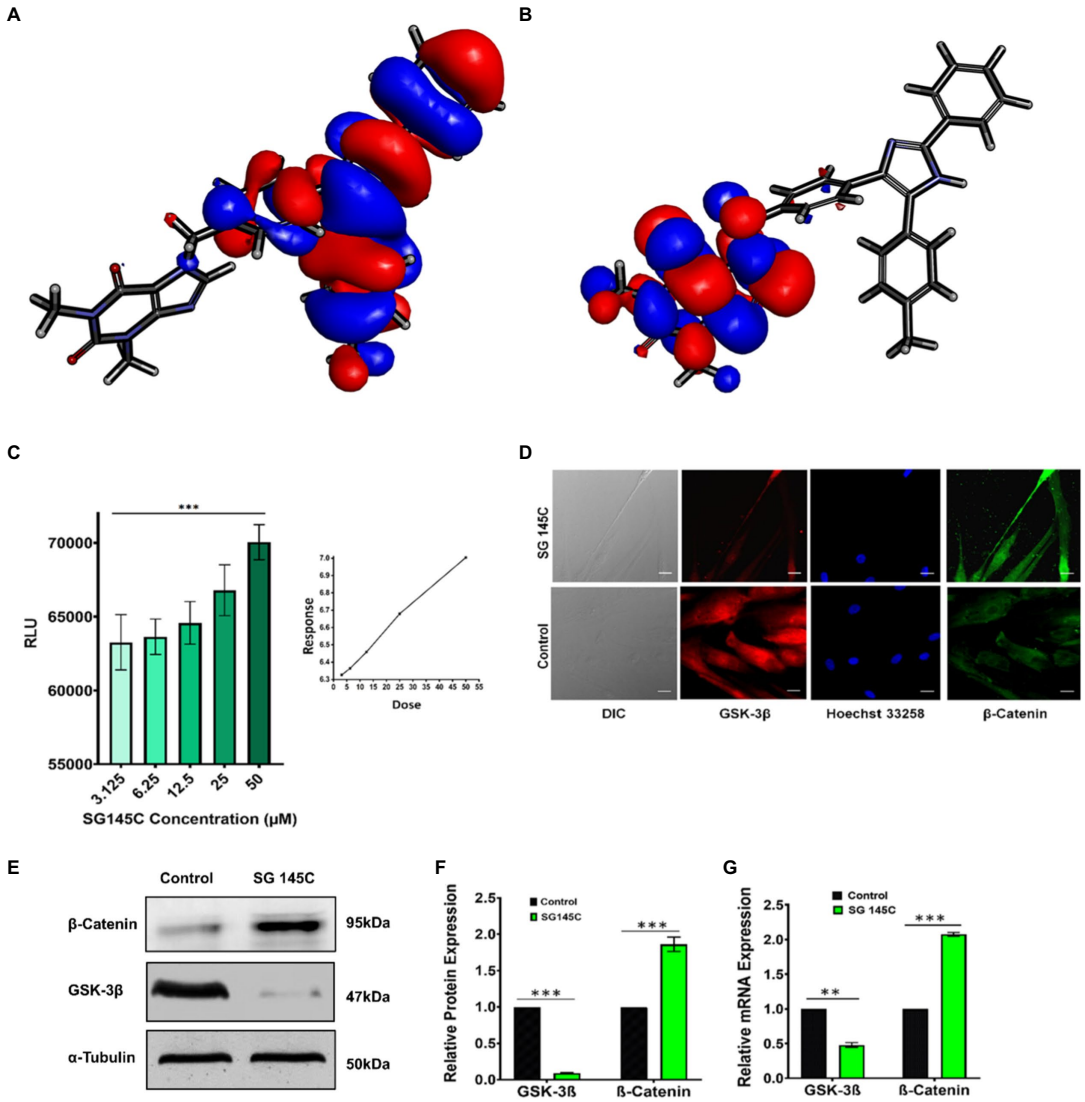
GSK-3β Inhibition by SG-145C results in increased β-Catenin levels. **(A,B)** HOMO-LUMO isosurface showing localization of orbitals. **(C)** Kinase-Glo luminescent assay to check the inhibition of GSK-3β revealed a direct dose-dependent inhibition. **(D)** Immunocytochemistry assay reveals the expression of GSK-3β (AF-488) and β-Catenin (AF-647) in differentiated neurons. Images were captured in 40× magnification where the scale bar corresponds to 20 μm. **(E,F)** Immunoblot and densitometric analysis and **(G)** qPCR to check the expression of two key regulators of Wnt Pathway, GSK-3β, and β-Catenin in response to SG-145C treatment. *n* = 3 for all sets of experiments and significance was calculated using student’s unpaired *t*-test. *p < 0.05; **p < 0.01; ***p < 0.001.

Interestingly, we were able to crystalize the lead compound SG-145C and the crystal structure was evaluated through a single crystal x-ray diffractometer (Supplementary Figure S23). The crystal structure of SG-145C was docked in a complex with GSK-3β and was exposed to CHARMM36 forcefield. After 20 ns of MD simulation, the structure was subjected to DFT calculation for understanding the HOMO-LUMO energy gap (Raya et al., 2011; Palomo et al., 2012). There are different reports of ligand-protein interaction at even quantum and electronic levels. The interaction is generally governed by the highest occupied molecular orbital (HOMO) and lowest unoccupied molecular orbital (LUMO) distribution (Matysiak, 2007). Generally, the key involvement is between the HOMO of the lead molecule and the LUMO of the receptor of interest and *vice-versa*. Also, stabilization of these interactions is through a lesser energy gap between the interacting orbitals and exactly the opposite in a drug molecule where the higher energy gap stabilizes the structure. The energy gap between HOMO-LUMO was calculated (Table 2). The DFT of the ligand-protein complex was performed through a 200 ns simulation in CHARMM36 forcefield to understand the final localization of the orbitals. The result of the MD simulation depicts that the ligand complex has lesser potential energy (−202,522 kcal/mol) than the receptor alone (−202,475 kcal/mol) and similar root means square deviation (RMSD) to that of the lone protein (Supplementary Figure S24). Further, DFT results show HOMO orbitals to be localized in the aromatic portion and HOMO to be located in the theophylline moiety of the molecule (Figures 6A,B). The above result demonstrates that the theophylline ring is an epicenter for electrophilic attack from amino acids of protein, on the other hand, the aromatic rings are acting as a nucleophilic center, which confirms the results of the docking study.

**Table 2:** DFT calculation of SG-145C.

COMPOUND	HOMO (eV)	LUMO (eV)	HLG (eV)
SG-145C	0.18852458	0.04655856	0.14196602

### Inhibition of GSK-3β and underlying mechanism of transdifferentiation

While we have established that indeed SG-145C is capable of transdifferentiating MSCs to neuronal cells, how it does so was our next question. It has already been stated above that the design rationale behind the formation of this imidazole-based library is the inhibition of GSK-3β, which is crucial for neuronal differentiation. Bioinformatics data has already been provided reflecting that SG-145C shows the best binding score at the GSK-3β pocket. It is known that GSK-3β is involved in different developmental events including neurogenesis, neuronal migration, survival, and differentiation (Marcus et al., 1998; Li et al., 2009; Luo, 2012). β isoform of GSK-3 is accountable for neurofibrillary tangle formation and Tau hyperphosphorylation, so molecules showing GSK-3β antagonistic property could be an optimistic approach for the treatment of Alzheimer’s disease (Bhat et al., 2004). A high throughput luminescent assay based on the Kinase Glo was performed. The system has been developed by Andrea et al., for screening against GSK-3β (Baki et al., 2007). This assay was performed to check the antagonist activity of SG-145C on GSK-3β. The luminescence signal is inversely correlated to the kinase activity. We found that, with the increasing dose of SG-145C, the luminescence signal increased parallelly, thus indicating the concentration-dependent activity of SG-145C on the inhibition of GSK-3β (Figure 6C). Western blot and qPCR revealed the reduction in GSK-3β along with an increase in β-catenin levels upon treatment of SG-145C for 7 days (Figures 6E–G). Immunocytochemistry data for the same conveyed that there was a reduction in GSK-3β along with an elevation in β-Catenin (Figure 6D). The activation of GSK-3β is regulated by phosphorylation at its different site so to check its reduced activity we checked the protein level of inactive GSK-3β (ser9) and found it to be significantly higher post-treatment with SG-145C for 7 days in comparison to undifferentiated MSCs. Total GSK-3β is reducing but the inactive form is increasing significantly in transdifferentiated MSCs (Supplementary Figure S27).

### The downstream pathway involved in the transdifferentiation process

**Figure 7:**
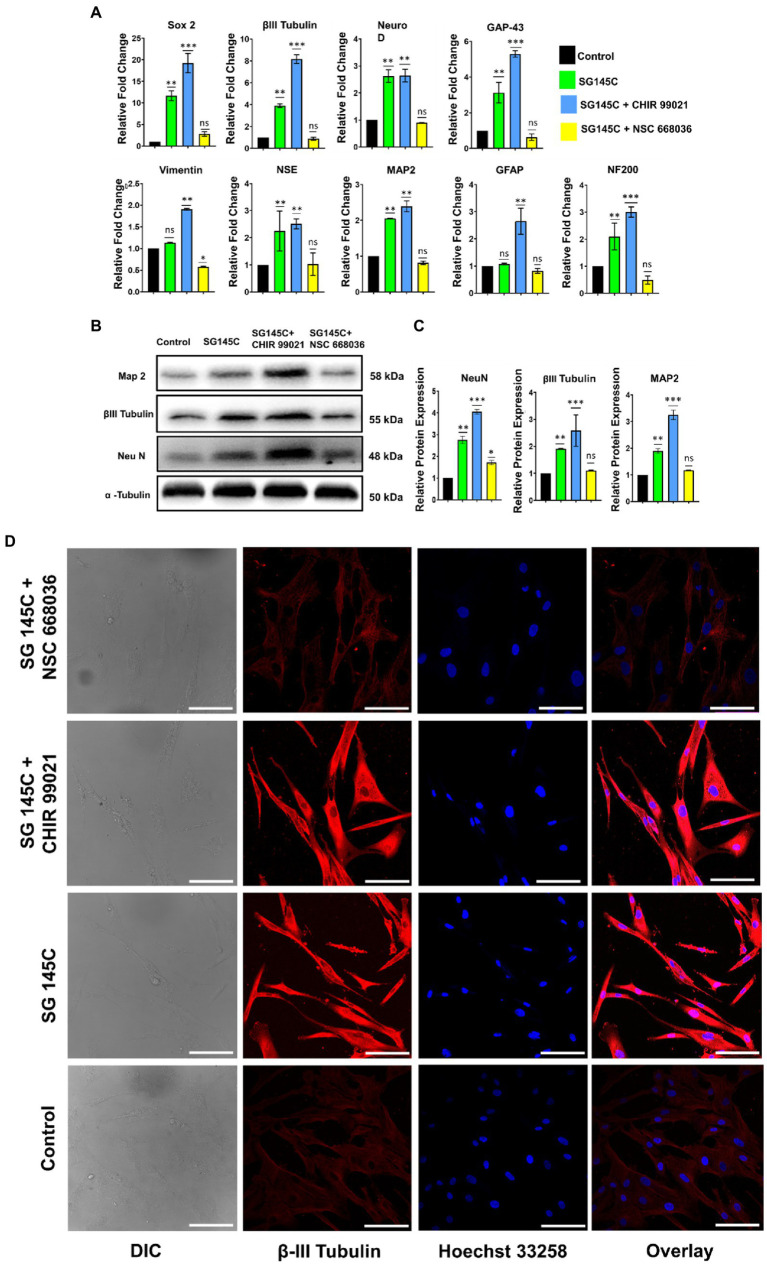
SG-145C induced trans-differentiation proceeds via the Wnt pathway. **(A)** qPCR and **(B,C)** immunoblot analysis comparing the expression of neuron-specific genes in response to SG-145C in combination with Wnt pathway inhibitor NSC668036 and commercially available GSK-3β inhibitor CHIR99021. **(D)** ICC staining was performed to compare the level of trans-differentiation in response to the mentioned inhibitors in combination with SG-145C. β-III Tubulin (AF-488) was used as neuronal marker proteins for visualization. Images were captured in 20X magnification where the scale bar corresponds to 70 µm. *n* = 3 for all sets of experiments and significance was calculated using one-way Anova *p < 0.05, **p < 0.01, ***p < 0.001.

To confirm the downstream pathway involved in the trans-differentiation process taking place through our molecule SG-145C and its effect was assessed on identified signaling pathways responsible for neurogenesis. Wnt signaling is known to promote neuronal differentiation from mesenchymal stem cells. GSK-3β and β-Catenin are the key regulators of the Wnt pathway, which is known to be a critical regulator of the transdifferentiation process (Clevers, 2006). MSCs were incubated with 5 μM of SG-145C with the Wnt Inhibitor NSC668036 (25 μϺ) for 7 days (Shan et al., 2005) and we observed a reduction in expression of the neuronal genes such as β-III Tubulin, Map-2, Neu N (Figures 7B,C). Parallelly RT-PCR data revealed that the expression of neuronal genes analyzed previously such as β-III Tubulin, Map-2, Neu N, Gap 43, Sox2, NF200, and NSE was reduced on blocking the Wnt pathway (Figure 7A). Immunocytochemistry data also revealed the reduced expression of neural-specific genes such as β-III Tubulin (Figure 7D). Since GSK-3β inhibition leads to the activation of Wnt signaling, therefore the collaborative effect of known GSK-3β Inhibitor CHIR99021 and our small molecule was also checked. Immunoblot analysis revealed increased levels of neuronal proteins β-III Tubulin, Map-2, Neu N (Figures 7B,C) while the expression of the β-III Tubulin, Map-2, Neu N, Gap 43, Sox2, NF200, and NSE genes increased significantly as seen *via* RT-PCR in comparison to SG-145C treatment only (Figure 7A). ICC data also reflected the increased expression of the neural gene β-III Tubulin (Figure 7D), corroborating the above RT-PCR and Immunoblot data.

### Electrophysiologically and spontaneously active neurons

The functionality of transdifferentiated hMSCs using our molecule SG-145C was checked using the patch clamp technique. Single-cell current clamp configuration was formed. Electrical stimulation was applied to generate a potential difference in the cells to be measured. Upon episodic stimulation of 50 ms spikes were generated. The transdifferentiated neurons generated action potentials which confirmed their functionality (Supplementary Figure S29). Spontaneous generation electrical impulses were seen for 3 min time without any interruption.

## Discussion

Neural development includes various complex processes such as cell proliferation, differentiation, and death. Any damage in the structural and functional integrity of neurons leads to progressive deterioration of the nervous system resulting in fatal neurodegenerative diseases. Neurons have no regenerative capacity therefore conventional therapies for neurological disorders lead to very poor outcomes. For the regeneration of damaged neural tissues, stem cell-based therapy has shown some potential in this field. MSCs may serve as a replacement for obtaining neural cells and are therefore being explored as an alternative in the therapy of neurological diseases. Wnt signaling is already known for playing an important role in neurogenesis. In the canonical Wnt pathway, Wnt interacts with the transmembrane protein called frizzled protein and initiates the cascade that suppresses the phosphorylation of GSK-3β consequently reducing the proteasomal degradation of β-catenin (Andrzejewska et al., 2021).

This modulation increases the activity of transcription factors guiding neuronal transdifferentiation. But in absence of Wnt, the formation of the APC complex takes place thus degrading the β-catenin protein.

To stop this degradation mechanism, here we have meticulously designed an imidazole-based GSK-3β inhibitor. Along with inhibiting the checkpoint of GSK-3β, the goal was also to develop first of its kind single-molecule transdifferentiation initiator that could surpass the flaws of chemical cocktail use. Inhibiting GSK-3β can decrease the phosphorylation of β-Catenin, thus increasing its presence in the downstream processes. For this purpose, we chose imidazole as our core moiety, as several studies have shown a beneficial effect of imidazole against neurodegenerative diseases (Cornec et al., 2017).

The whole concept was abstracted tactically using a ligand-based 3D-pharmacophore drug design strategy mainly targeting GSK-3β. Screened molecules were put through docking to determine the binding energy. To check the toxicity profile, ADME screening was performed, pharmacokinetic profile often corresponds to the toxicity of novel leads and could become a drawback for the molecules in the clinical trials. Thus, early detection is always necessary for the confirmed success of the designs. ADME result and docking energy revealed SG-145C to be the best-fitted molecule along with nine other molecules. Further, we found the crystal structure of the lead molecule SG-145C. DFT calculation with the final confirmation of MD simulation showed us the HOMO-LUMO distribution within the molecule and its importance in ligand interaction. Next, all the 10 shortlisted molecules from docking studies were checked for cytocompatibility *via* MTT Assay. The molecules which were found to be non-cytotoxic in lower doses were chosen for biological studies as they showed adequacy for cell culture applications. The shortlisted 9 molecules were carried on to Immuno-cytochemistry (ICC) studies to validate their differentiation capabilities. It was observed that SG-145C showed the most significant elevation in the expression of the neuron-specific proteins, *β* III-tubulin and Map2 compared to that of the other 8 molecules. Therefore, using neuronal morphology and fluorescence intensity as the attributes of neural trans-differentiation, SG-145C was selected as the lead candidate for further validation studies. This validated our in-silico modeling prediction and we proceeded with SG-145C for understanding the mechanism guiding transdifferentiation.

**Figure 8:**
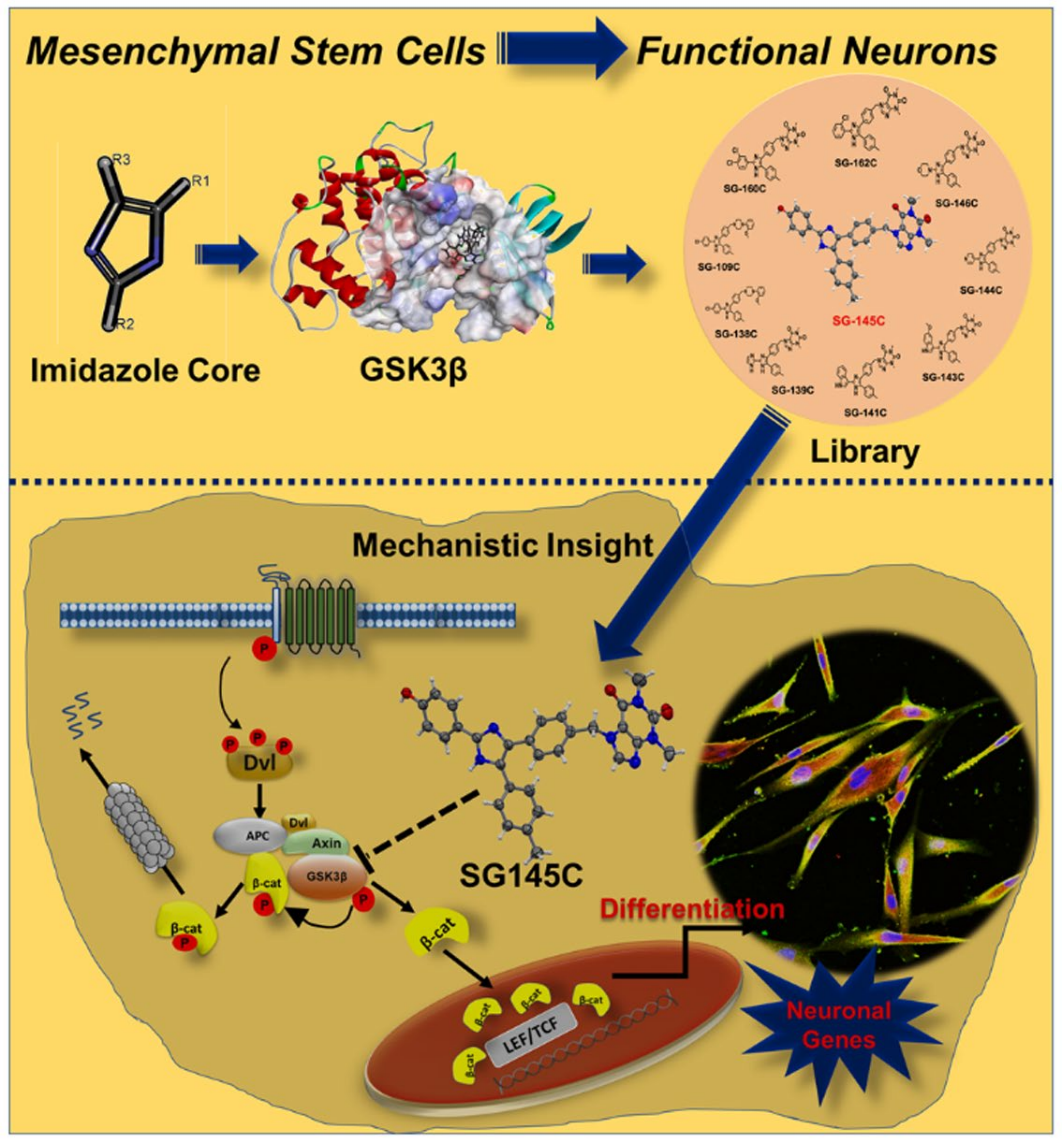
Design and development of Imidazole-based small molecule library which has the ability to transdifferentiate Human Mesenchymal Stem Cells into neurons by altering the components of canonical Wnt pathway that is inhibition of activation of GSK-3β. Because of the inhibition of GSK-3β, its activity of phosphorylating and degrading β-catenin is also inhibited. As a result of which β-catenin is stabilized, which allows it to translocate to the nucleus and transcribe the downstream neuronal genes after interacting with the TCF/LEF family of transcription factors.

The transdifferentiation was validated further using a combination of western blotting, RT-PCR, and FACS analysis to quantify the changes as a result of SG-145C treatment. Neuronal proteins were found to be significantly upregulated but astrocyte marker GFAP and MSC-specific vimentin showed no significant change. Thus, the specificity of transdifferentiation of MSCs to Neuronal cells was established. The gradual increase in the expression of neuronal proteins as a result of SG-145C reveals the time-dependent nature of the process. The proliferative nature of the cells was checked using BrdU assay while cell death markers showed no significant change over 7 days of treatment with SG-145C. Thus, the results were seen in a healthy population of cells under proliferating conditions. Kinase Glo. based luminescent assay revealed the inverse correlation of the result with GSK-3β activity, there is a significant decrease in GSK-3β activity with an increase in SG-145C concentration. Western blot and qPCR also revealed the reduction in GSK-3β along with an increase in β-catenin levels as a result of SG-145C treatment for 7 days. Moreover, the inactive form of GSK-3β, i.e., p-GSK-3β (Ser9) was observed to be significantly increased after the treatment of SG-145C for 7 days showing distinctly the role of SG-145C in the inhibition of GSK-3β. Therefore, because of the inhibition of GSK-3β, its activity of phosphorylating and degrading β-catenin is also inhibited. As a result of which β-catenin is stabilized, which allows it to translocate to the nucleus and transcribe the downstream neuronal genes after interacting with the TCF/LEF family of transcription factors (Wu et al., 2009) (Figure 8). With the advancement of the differentiation process the GSK-3β protein levels decreased along with an increase in β-catenin. Additionally, the downregulation of GSK-3β mRNA in response to SG-145C points toward a systemic shift to maintain the milieu to promote transdifferentiation. Wnt signaling is known for promoting neuronal differentiation from Mesenchymal Stem cells, so to understand the downstream pathway involved in this process, its effect was studied on the Wnt pathway since GSK-3β is a key regulator of the Wnt pathway. As shown in the results on blocking the Wnt pathway, expression of the neuron-specific genes was significantly reduced stating the involvement of this pathway in Neurogenesis. Since GSK-3β inhibition leads to activation of the Wnt pathway, so the synergistic effect of our molecule and known GSK-3β inhibitor (CHIR99021) was analyzed which showed the enhancement in the expression of neural genes. Thus, we can postulate that SG-145C promotes transdifferentiation *via* a reduction in GSK-3β levels which results in increased Wnt activity.

From our studies, we find that while SG-145C inhibits GSK-3β it also downregulates GSK-3β at both gene and protein levels to induce transdifferentiation. There are commercially available GSK-3β inhibitors but they aren’t capable of transdifferentiation on their own and that is why we understand that there are other factors at play here which contribute toward the observed phenotype. We find the continued downregulation of GSK-3β as one of the primary requirements and factors driving the transdifferentiation process. Understanding the mechanistic pathways to understand the applicability of SG-145C in *in vivo* conditions will be one of our future goals.

Many studies showing neuronal differentiation only analyse the expression pattern of neuronal proteins which cannot be satisfactory in terms of neuronal activity. Established neurons in culture have to be investigated in terms of functionality since the expression of neuronal proteins does not indicate their functional roles.

So, once the transdifferentiating ability of our molecule was established, the generated neurons were checked for functionality through the Patch clamp technique. We found that our transdifferentiated neurons are electrophysiologically active, as they generated the action potential. This proves their ability to be used as a tool for disease modeling in neuropathologies, and regenerative medicine and can also be used to understand the differentiation mechanism.

Considering all these exciting results, we showcased that our discovery reveals for the first time that neuronal transdifferentiation is possible using a single synthetic small molecule rather than using a chemical cocktail along with the growth factors. To the best of our knowledge, this is a first discovery, which has tremendous potential in the development of neuro-regenerative medicine and we envision that our work will be considered as a potential neurotherapeutic foresight.

## Data availability statement

The datasets presented in this study can be found in online repositories. The names of the repository/repositories and accession number(s) can be found in the article/Supplementary material.

## Ethics statement

The studies involving human participants were reviewed and approved by Stem Cell Ethics Committee, CSIR-Indian Institute of Chemical Biology, Kolkata, India. Written informed consent for participation was not required for this study in accordance with the national legislation and the institutional requirements.

## Author contributions

VG performed all the cell-based experiments and *in vitro* assays. TM performed the synthesis, purification, and characterization of the molecules. RR performed molecular docking, pharmacophoric studies, and DFT calculations. PKG performed the crystallization study. AJ and ShG helped to analyze the data. SG conceived the idea and supervised the project. SG, VG, and RR wrote the manuscript. All authors contributed to the article and approved the submitted version.

## Conflict of interest

The authors declare that the research was conducted in the absence of any commercial or financial relationships that could be construed as a potential conflict of interest.

## Publisher’s note

All claims expressed in this article are solely those of the authors and do not necessarily represent those of their affiliated organizations, or those of the publisher, the editors and the reviewers. Any product that may be evaluated in this article, or claim that may be made by its manufacturer, is not guaranteed or endorsed by the publisher.

## Abbreviations

hMSC: Human Mesenchymal Stem Cells
NPC: Neural Progenitor cells
HOMO: Highest Occupied Molecular Orbital
LUMO: lowest unoccupied molecular orbital
GSK-3β: Glycogen Synthase Kinase 3β
RT-PCR: reverse transcription-polymerase chain reaction
FACS: Fluorescence-activated Cell Sorting
ICC: Immunocytochemistry
SDS-PAGE: sodium dodecyl sulfate polyacrylamide gel electrophoresis
NSE: Neuron specific Enolase
NF200: Neurofilament 200
GFAP: Glial Fibrillary acidic protein
DFT: Density functional theory

## References

[ref1] Almudena F. M. María L. M. María Salomé S. P. José Manuel G. V. Jesús A. Félix H. (2013). Dual effects of increased glycogen synthase kinase-3β activity on adult neurogenesis. Hum. Mol. Genet. 22, 1300–1315. doi: 10.1093/hmg/dds533 23257288

[ref2] Andrzejewska A. Dabrowska S. Lukomska B. Janowski M. (2021). Mesenchymal stem cells for neurological disorders. Adv. Sci. 8:2002944. doi: 10.1002/advs.202002944 PMC8024997 33854883

[ref3] Baki A. Bielik A. Molnár L. Szendrei G. Keserü G. M. (2007). A high throughput luminescent assay for glycogen synthase kinase-3beta inhibitors. Assay Drug Dev. Technol. 5, 75–83. doi: 10.1089/adt.2006.029 17355201

[ref4] Bhat R. V. Budd Haeberlein S. L. Avila J. (2004). Glycogen synthase kinase 3: a drug target for CNS therapies. J. Neurochem. 89, 1313–1317. doi: 10.1111/j.1471-4159.2004.02422.x 15189333

[ref5] Bradley C. A. Peineau S. Taghibiglou C. Nicolas C. S. Whitcomb D. J. Bortolotto Z. A.. (2012). A pivotal role of GSK-3 in synaptic plasticity. Front. Mol. Neurosci. 5:13. doi: 10.3389/fnmol.2012.00013 22363262 PMC3279748

[ref6] Brooks B. R. Brooks C. L. Mackerell A. D. J. Nils-son L. Petrella R. J. Roux B.. (2009). CHARMM: the biomolecular simulation program. J. Comput. Chem. 30, 1545–1614. doi: 10.1002/jcc.21287 19444816 PMC2810661

[ref7] Chen Y. Jiang J. (2013). Decoding the phosphorylation code in hedgehog signal transduction. Cell Res. 23, 186–200. doi: 10.1038/cr.2013.10 23337587 PMC3567827

[ref8] Clay L. Caudron F. Denoth-Lippuner A. Boettcher B. Buvelot Frei S. Snapp E. L.. (2014). A sphingolipid-dependent diffusion barrier confines ER stress to the yeast mother cell. eLife 3:e01883. doi: 10.7554/eLife.01883 24843009 PMC4009826

[ref9] Clevers H. (2006). Wnt/Beta-catenin signaling in development and disease. Cells 127, 469–480. doi: 10.1016/j.cell.2006.10.018 17081971

[ref10] Cornec A.-S. Monti L. Kovalevich J. Makani V. James M. J. Vijayendran K. G.. (2017). Multitargeted Imidazoles: potential therapeutic leads for Alzheimer’s and other neurodegenerative diseases. J. Med. Chem. 60, 5120–5145. doi: 10.1021/acs.jmedchem.7b00475 28530811 PMC5483893

[ref11] Cortés-Medina L. V. Pasantes-Morales H. Aguilera-Castrejon A. Picones A. Lara-Figueroa C. O. Luis E.. (2019). Neuronal Transdifferentiation potential of human mesenchymal stem cells from neonatal and adult sources by a small molecule cocktail. Stem Cells Int.:7627148. doi: 10.1155/2019/7627148 31065279 PMC6466843

[ref12] Ding S. Schultz P. G. (2004). A role for chemistry in stem cell biology. Nat. Biotechnol. 22, 833–840. doi: 10.1038/nbt987 15229546

[ref13] Divya M. S. Roshin G. E. Divya T. S. Rasheed V. A. Santhoshkumar T. R. Elizabeth K. E.. (2012). Umbilical cord blood-derived mesenchymal stem cells consist of a unique population of progenitors co-expressing mesenchymal stem cell and neuronal markers capable of instantaneous neuronal differentiation. Stem Cell Res. Ther. 3:57. doi: 10.1186/scrt148 23253356 PMC3580487

[ref14] Doble B. W. Woodgett J. R. (2003). GSK-3: tricks of the trade for a multi-tasking kinase. J. Cell Sci. 116, 1175–1186. doi: 10.1242/jcs.00384 12615961 PMC3006448

[ref15] Emamian E. S. Hall D. Birnbaum M. J. Karayiorgou M. Gogos J. A. (2004). Convergent evidence for impaired AKT1-GSK3beta signaling in schizophrenia. Nat. Genet. 36, 131–137. doi: 10.1038/ng1296 14745448

[ref16] Gonçalves J. T. Schafer S. T. Gage F. H. (2016). Adult neuro-genesis in the hippocampus: from stem cells to behavior. Cells 167, 897–914. doi: 10.1016/j.cell.2016.10.021 27814520

[ref17] Harris V. K. Yan Q. J. Vyshkina T. Sahabi S. Liu X. Sadiq S. A. (2012). Clinical and pathological effects of intrathecal injection of mesenchymal stem cell derived neural progenitors in an experimental model of multiple sclerosis. J. Neurol. Sci. 313, 167–177. doi: 10.1016/j.jns.2011.08.036, PMID: 21962795

[ref18] Hur E.-M. Zhou F.-Q. (2010). GSK3 Signalling in neural development. Nat. Rev. Neurosci. 11, 539–551. doi: 10.1038/nrn2870 20648061 PMC3533361

[ref19] Jope R. S. Roh M.-S. (2006). Glycogen synthase Kinase-3 (GSK3) in psychiatric diseases and therapeutic interventions. Curr. Drug Targets 7, 1421–1434. doi: 10.2174/1389450110607011421 17100582 PMC1850891

[ref20] Jung K. H. Shin H. P. Lee S. Lim Y. J. Hwang S. H. Han H.. (2009). Effect of human umbilical cord blood-derived mesenchymal stem cells in a cirrhotic rat model. Liver Int. 29, 898–909. doi: 10.1111/j.1478-3231.2009.02031.x 19422480

[ref21] Kim G. H. Halder D. Park J. Namkung W. Shin I. (2014). Imidazole-based small molecules that promote neurogenesis in pluripotent cells. Angew. Chem. 35, 9425–9428. doi: 10.1002/anie.201404871 25044422

[ref22] Kimura T. Yamashita S. Nakao S. Park J.-M. Murayama M. Mizoroki T.. (2008). GSK-3beta is required for memory Reconsol-idation in adult brain. PLoS One 3:e3540. doi: 10.1371/journal.pone.0003540 18958152 PMC2568810

[ref23] Klein P. S. Melton D. A. (1996). A molecular mechanism for the effect of lithium on development. Proc. Natl. Acad. Sci. U. S. A. 93, 8455–8459. doi: 10.1073/pnas.93.16.8455 8710892 PMC38692

[ref24] Kruminis-Kaszkiel E. Osowski A. Bejer-Oleńska E. Dziekoński M. Wojtkiewicz J. (2020). Differentiation of human mesenchymal stem cells from Wharton’s jelly towards neural stem cells using a feasible and repeatable protocol. Cells 9:3. doi: 10.3390/cells9030739 PMC7140706 32192154

[ref25] Lauretti E. Dincer O. Praticò D. (2020). Glycogen synthase kinase-3 signaling in Alzheimer's disease. Biochim. Biophys. Acta Mol. Cell Res. 1867:118664. doi: 10.1016/j.bbamcr.2020.118664 32006534 PMC7047718

[ref26] Li X.-J. Zhang X. Johnson M. A. Wang Z.-B. Lavaute T. Zhang S.-C. (2009). Coordination of sonic hedgehog and Wnt signaling determines ventral and dorsal Telencephalic neuron types from human embryonic stem cells. Development 136, 4055–4063. doi: 10.1242/dev.036624 19906872 PMC2778748

[ref27] Lindvall O. Kokaia Z. (2010). Stem cells in human neurodegenerative disorders–time for clinical translation? J. Clin. Invest. 120, 29–40. doi: 10.1172/JCI40543, PMID: 20051634 PMC2798697

[ref28] Liu S. Li C. Xing Y. Tao F. (2014). Effect of transplantation of human embryonic stem cell-derived neural progenitor cells on adult neurogenesis in aged hippocampus. Am. J. Stem Cells 3, 21–26. 24660111 PMC3960754

[ref29] Llorens-Martín M. Jurado J. Hernández F. Avila J. (2014). GSK-3β, a pivotal kinase in Alzheimer disease. Front. Mol. Neurosci. 21:46. doi: 10.3389/fnmol.2014.00046 PMC4033045 24904272

[ref30] Luo J. (2012). The role of GSK3beta in the development of the central nervous system. Front. Biol. (Beijing) 7, 212–220. doi: 10.1007/s11515-012-1222-2 25688261 PMC4327837

[ref31] MacDonald B. T. Tamai K. He X. (2009). Wnt/Beta-catenin signaling: components, mechanisms, and diseases. Dev. Cell 17, 9–26. doi: 10.1016/j.devcel.2009.06.016 19619488 PMC2861485

[ref32] Marcus E. A. Kintner C. Harris W. (1998). The role of GSK3beta in regulating neuronal differentiation in Xenopus Laevis. Mol. Cell. Neurosci. 12, 269–280. doi: 10.1006/mcne.1998.0713 9828091

[ref33] Matysiak J. (2007). Evaluation of electronic, lipophilic and membrane affinity effects on Antiproliferative activity of 5-Substituted-2-(2,4-Dihydroxyphenyl)-1,3,4-Thiadiazoles against various human cancer cells. Eur. J. Med. Chem. 42, 940–947. doi: 10.1016/j.ejmech.2006.12.033 17320247

[ref34] McCubrey J. A. Rakus D. Gizak A. Steelman L. S. Abrams S. L. Lertpiriyapong K.. (2016). Effects of mutations in Wnt/β-catenin, hedgehog, Notch and PI3K pathways on GSK-3 activity-diverse effects on cell growth, metabolism and cancer. Biochim. Biophys. Acta 1863, 2942–2976. doi: 10.1016/j.bbamcr.2016.09.004 27612668

[ref35] Palomo V. Perez D. I. Perez C. Morales-Garcia J. A. Soteras I. AlonsoGil S.. (2012). 5-Imino-1,2,4-Thiadiazoles: first small molecules as substrate competitive inhibitors of glycogen synthase kinase 3. J. Med. Chem. 55, 1645–1661. doi: 10.1021/jm201463v 22257026

[ref36] Rafieemehr H. Kheirandish M. Soleimani M. (2016). Neural differentiation of human umbilical cord blood-derived mesenchymal stem cells. Avicenna J. Med. Biochem. 4, 5–29066. doi: 10.17795/ajmb-29066

[ref37] Raya A. Barrientos-Salcedo C. Rubio-Póo C. Soriano-Correa C. (2011). Electronic structure evaluation through Quan-tum chemical descriptors of 17β-Aminoestrogens with an anticoagulant effect. Eur. J. Med. Chem. 46, 2463–2468. doi: 10.1016/j.ejmech.2011.03.032 21481988

[ref38] Roy K. Ambure P. Kar S. (2018). How precise are our quantitative structure-activity relationship derived predictions for new query chemicals? ACS Omega 3, 11392–11406. doi: 10.1021/acsomega.8b01647 31459245 PMC6645132

[ref39] Sacchetti B. Funari A. Remoli C. Giannicola G. Kogler G. Liedtke S.. (2016). No identical “mesenchymal stem cells” at different times and sites: human committed progenitors of distinct origin and differentiation potential are incorporated as adventitial cells in microvessels. Stem Cell Rep. 6, 897–913. doi: 10.1016/j.stemcr.2016.05.011 PMC4912436 27304917

[ref40] Salado-Manzano C. Perpiña U. Straccia M. Molina-Ruiz F. J. Cozzi E. Rosser A. E.. (2020). Is the immunological response a bottleneck for cell therapy in neurodegenerative diseases? Front. Cell. Neurosci. 14:250. doi: 10.3389/fncel.2020.00250 32848630 PMC7433375

[ref41] Schön U. Antel J. Brückner R. Messinger J. Franke R. Gruska A. (1998). Synthesis, pharmacological characterization, and quantitative structure-activity relationship analyses of 3, 7,9,9-Tetraalkylbispidines: derivatives with specific bradycardic activity. J. Med. Chem. 41, 318–331. doi: 10.1021/jm970120q 9464363

[ref42] Shan J. Shi D.-L. Wang J. Zheng J. (2005). Identification of a specific inhibitor of the Dishevelled PDZ domain. Biochemistry 44, 15495–15503. doi: 10.1021/bi0512602 16300398

[ref43] Singh S. Srivastava A. Srivastava P. Dhuriya Y. K. Pandey A. Kumar D.. (2016). Advances in stem cell research-a ray of Hope in better diagnosis and prognosis in neurodegenerative diseases. Front. Mol. Biosci. 3:72. doi: 10.3389/fmolb.2016.00072, PMID: 27878120 PMC5099954

[ref44] Thompson R. Casali C. Chan C. (2019). Forskolin and IBMX induce neural Transdifferentiation of MSCs through downregulation of the NRSF. Sci. Rep. 9:2969. doi: 10.1038/s41598-019-39544-0 30814572 PMC6393535

[ref45] Urenjak J. Williams S. R. Gadian D. G. Noble M. (1992). Specific expression of N-Acetylaspartate in neurons, oligodendrocyte-Type-2 astrocyte progenitors, and immature oligodendrocytes in vitro. J. Neurochem. 59, 55–61. doi: 10.1111/j.1471-4159.1992.tb08875.x 1613513

[ref46] Večeřa J. Procházková J. Šumberová V. Pánská V. Paculová H. Lánová M. K.. (2020). Hypoxia/Hif1α prevents premature neuronal differentiation of neural stem cells through the activation of Hes1. Stem Cell Res. 45:101770. doi: 10.1016/j.scr.2020.101770 32276221

[ref47] Vignaux P. A. Minerali E. Foil D. H. Puhl A. C. Ekins S. (2020). Machine learning for discovery of GSK3β inhibitors. ACS Omega 5, 26551–26561. doi: 10.1021/acsomega.0c03302 33110983 PMC7581251

[ref48] Voeltz G. K. Prinz W. A. Shibata Y. Rist J. M. Rapoport T. A. (2006). A class of membrane proteins shaping the tubular endoplasmic reticulum. Cells 124, 573–586. doi: 10.1016/j.cell.2005.11.047 16469703

[ref49] Wu G. Huang H. Garcia Abreu J. He X. (2009). Inhibition of GSK3 phosphorylation of Beta-catenin via phosphorylated PPPSPXS motifs of Wnt Coreceptor LRP6. PLoS One 4:e4926. doi: 10.1371/journal.pone.0004926 19293931 PMC2654145

[ref50] Yang J. Yan Y. Ma C.-G. Kang T. Zhang N. Gran B.. (2012). Accelerated and enhanced effect of CCR5-transduced bone marrow neural stem cells on autoimmune encephalomyelitis. Acta Neuropathol. 124, 491–503. doi: 10.1007/s00401-012-0989-1 22526024 PMC3544339

[ref51] Zhang H.-X. Li Y. Wang X. Wang Y.-H. (2012). Probing the structural requirements of A-type Aurora kinase inhibitors using 3D-QSAR and molecular docking analysis. J. Mol. Model. 18, 1107–1122. doi: 10.1007/s00894-011-1042-3 21670994

