# Supplementary material for: Discovery of imidazole-based GSK-3β inhibitors for transdifferentiation of human mesenchymal stem cells to neurons: A potential single-molecule neurotherapeutic foresight
Source: Front Mol Neurosci. 2022 Dec 15;15:1002419. doi: 10.3389/fnmol.2022.1002419 (PMC9797524; doi:10.3389/fnmol.2022.1002419)

checkCIF/PLATON report

You have not supplied any structure factors. As a result the full set of tests cannot be run.

THIS REPORT IS FOR GUIDANCE ONLY. IF USED AS PART OF A REVIEW PROCEDURE FOR PUBLICATION, IT SHOULD NOT REPLACE THE EXPERTISE OF AN EXPERIENCED CRYSTALLOGRAPHIC REFEREE.

No syntax errors found.   CIF dictionary   Interpreting this report

Datablock: sg_145c_0m_a

```
Bond precision:   C-C = 0.0055 A          Wavelength=1.54184

Cell:             a=12.8005(7)     b=21.3557(12)      c=11.5984(6)
                  alpha=90         beta=96.507(3)     gamma=90
Temperature:      101 K
```

	Calculated	Reported
Volume	3150.2(3)	3150.2(3)
Space group	P 21/c	P 1 21/c 1
Hall group	-P 2ybc	-P 2ybc
Moiety formula	C30 H26 N6 O3, 2(C2 H3 N)	C30 H26 N6 O3, 2(C2 H3 N)
Sum formula	C34 H32 N8 O3	C34 H32 N8 O3
Mr	600.68	600.67
Dx,g cm-3	1.266	1.267
Z	4	4
Mu (mm-1)	0.682	0.682
F000	1264.0	1264.0
F000'	1267.75	
h,k,lmax	15,25,13	15,25,13
Nref	5375	5335
Tmin,Tmax	0.849,0.903	0.608,0.753
Tmin'	0.849	

```
Correction method= # Reported T Limits: Tmin=0.608 Tmax=0.753
AbsCorr = MULTI-SCAN

Data completeness= 0.993           Theta(max)= 65.098

R(reflections)= 0.0855( 3827)      wR2(reflections)= 0.2425( 5335)

S = 1.084                 Npar= 413
```

The following ALERTS were generated. Each ALERT has the format
       test-name_ALERT_alert-type_alert-level.
Click on the hyperlinks for more details of the test.

Alert level C

```
DIFMX02_ALERT_1_C  The maximum difference density is > 0.1*ZMAX*0.75
            The relevant atom site should be identified.
THETM01_ALERT_3_C  The value of sine(theta_max)/wavelength is less than 0.590
            Calculated sin(theta_max)/wavelength =    0.5883
PLAT097_ALERT_2_C Large Reported Max.  (Positive) Residual Density       0.69 eA-3
PLAT244_ALERT_4_C Low    'Solvent' Ueq as Compared to Neighbors of       C2_2 Check
PLAT244_ALERT_4_C Low    'Solvent' Ueq as Compared to Neighbors of       C2_3 Check
PLAT260_ALERT_2_C Large Average Ueq of Residue Including      N1_2      0.199 Check
PLAT260_ALERT_2_C Large Average Ueq of Residue Including      N1_3      0.108 Check
PLAT340_ALERT_3_C Low Bond Precision on  C-C Bonds ...............    0.00546 Ang.
```

Alert level G

```
PLAT007_ALERT_5_G Number of Unrefined Donor-H Atoms ..............          2 Report
PLAT012_ALERT_1_G No       _shelx_res_checksum Found in CIF ......     Please Check
PLAT072_ALERT_2_G SHELXL First  Parameter in WGHT  Unusually Large       0.14 Report
PLAT720_ALERT_4_G Number of Unusual/Non-Standard Labels ..........         77 Note
PLAT883_ALERT_1_G No Info/Value for _atom_sites_solution_primary .     Please Do !
```

   0 **ALERT level A** = Most likely a serious problem - resolve or explain
   0 **ALERT level B** = A potentially serious problem, consider carefully
   8 **ALERT level C** = Check. Ensure it is not caused by an omission or oversight
   5 **ALERT level G** = General information/check it is not something unexpected

```
   3 ALERT type 1 CIF construction/syntax error, inconsistent or missing data
   4 ALERT type 2 Indicator that the structure model may be wrong or deficient
   2 ALERT type 3 Indicator that the structure quality may be low
   3 ALERT type 4 Improvement, methodology, query or suggestion
   1 ALERT type 5 Informative message, check
```

It is advisable to attempt to resolve as many as possible of the alerts in all categories. Often the minor alerts point to easily fixed oversights, errors and omissions in your CIF or refinement strategy, so attention to these fine details can be worthwhile. In order to resolve some of the more serious problems it may be necessary to carry out additional measurements or structure refinements. However, the purpose of your study may justify the reported deviations and the more serious of these should normally be commented upon in the discussion or experimental section of a paper or in the "special_details" fields of the CIF. checkCIF was carefully designed to identify outliers and unusual parameters, but every test has its limitations and alerts that are not important in a particular case may appear. Conversely, the absence of alerts does not guarantee there are no aspects of the results needing attention. It is up to the individual to critically assess their own results and, if necessary, seek expert advice.

Publication of your CIF in IUCr journals

A basic structural check has been run on your CIF. These basic checks will be run on all CIFs submitted for publication in IUCr journals (*Acta Crystallographica*, *Journal of Applied Crystallography*, *Journal of Synchrotron Radiation*); however, if you intend to submit to *Acta Crystallographica Section C* or *E* or *IUCrData*, you should make sure that full publication checks are run on the final version of your CIF prior to submission.

Publication of your CIF in other journals

Please refer to the *Notes for Authors* of the relevant journal for any special instructions relating to CIF submission.

PLATON version of 13/07/2021; check.def file version of 13/07/2021

Datablock sg_145c_0m_a - ellipsoid plot

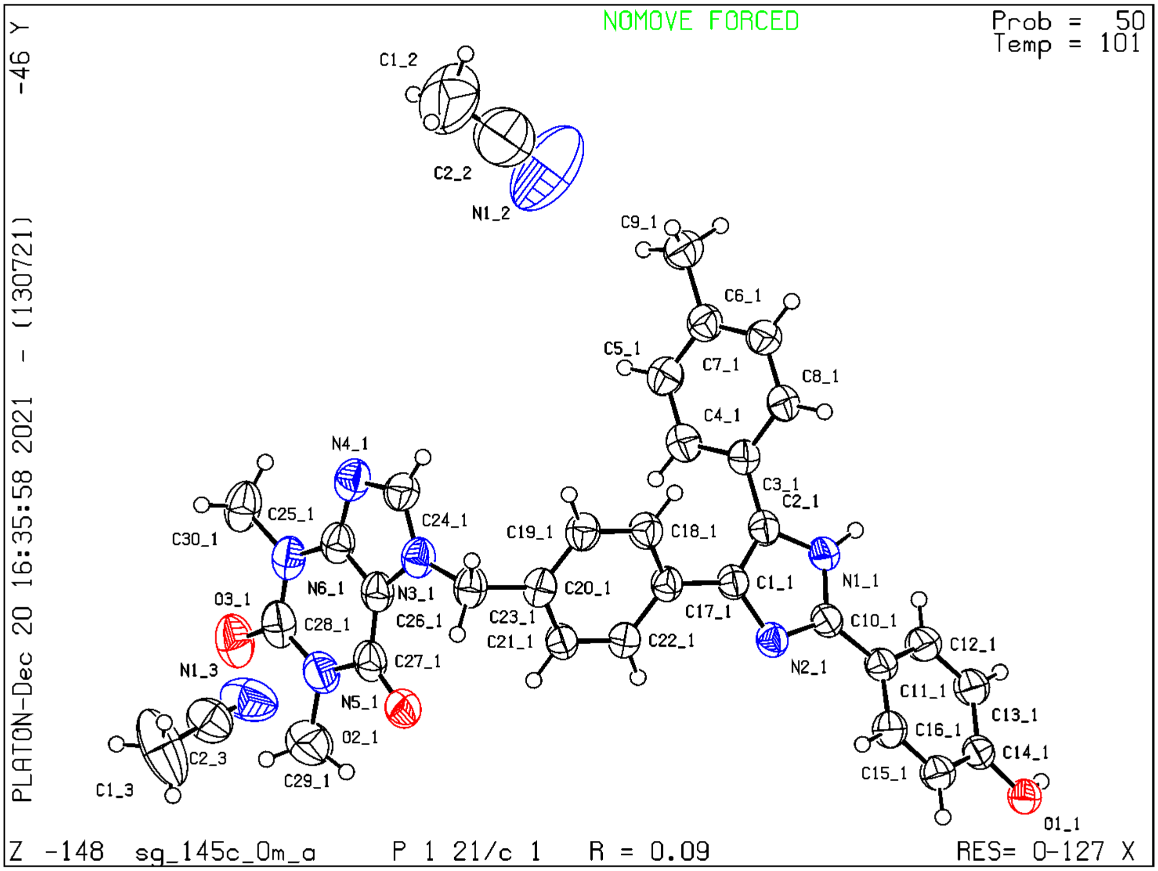

Supplement: Supplementary file 1 [file Data_Sheet_1.PDF]
