# Supplementary material for: Discovery of imidazole-based GSK-3β inhibitors for transdifferentiation of human mesenchymal stem cells to neurons: A potential single-molecule neurotherapeutic foresight
Source: Front Mol Neurosci. 2022 Dec 15;15:1002419. doi: 10.3389/fnmol.2022.1002419 (PMC9797524; doi:10.3389/fnmol.2022.1002419)
Supplement: Supplementary file 2 [file Data_Sheet_2.pdf]

<u>**Electronic Supplementary Information**</u>

Discovery of Imidazole-based GSK-3β Inhibitors for Transdifferentiation of Human Mesenchymal Stem Cells to Neurons: A Potential single molecule Neurotherapeutic Foresight

Varsha Gupta,[1] Tanushree Mahata,[1] Rajsekhar Roy,[2] Prabir Kumar Gharai,[1] Aniket Jana,[3] Shubham Garg,[2] Surajit Ghosh*[1,2,3]

1. Organic and Medicinal Chemistry and Structural Biology and Bioinformatics Division, CSIR-Indian Institute ofChemical Biology, 4, Raja S. C. Mullick Road, Jadavpur, Kolkata-700 032, WB, India. Fax: +91-33-2473- 5197/0284; Tel: +91-33-2499-5872

2. Department of Bioscience & Bioengineering, Indian Institute of Technology Jodhpur, NH 65, Surpura BypassRoad, Karwar, Rajasthan 342037, India, Phone: +91-291-280-1212

3. Smart Healthcare, Interdisciplinary Research Platform, Indian Institute of Technology Jodhpur, NH 62, Surpura Bypass Road, Karwar, Rajasthan 342037, India, Phone: +91-291-280-1212

Experimental Procedure

Method A:

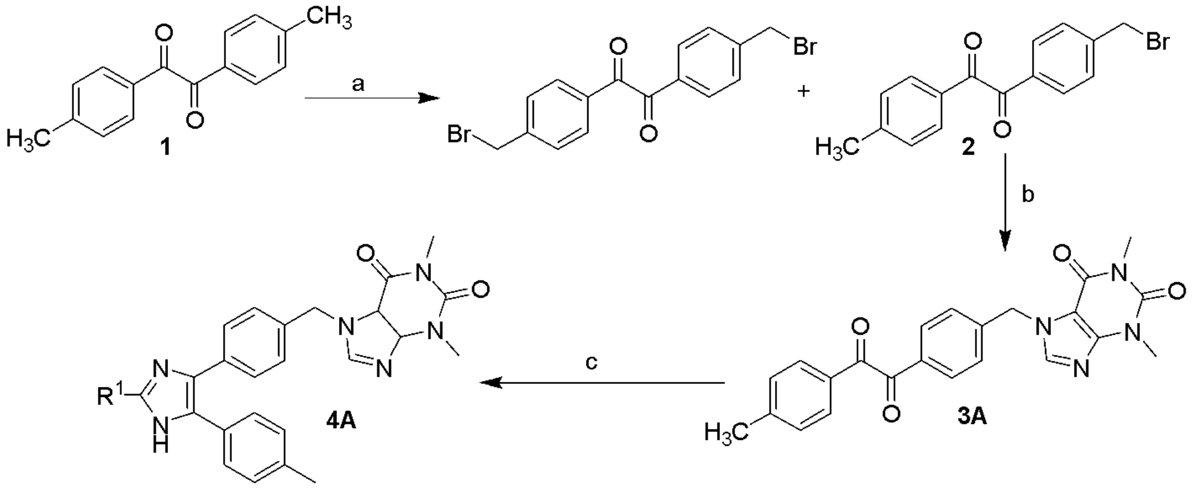

(a) NaBr, H_2O_2, H_2SO_4, $CHCl_3$; (b) Theophylline, K_2CO_3, DMF; (c) R_1CHO, NH_4OAc, AcOH, Reflux

Scheme S1. General scheme of cyclization with compound 3A

Synthesis of compound 2: 4, 4′-Dimethylbenzil (2.38 g; 10 mmol) and Sodium bromide (1.13 g; 11 mmol) were taken and added HPLC chloroform (40 ml) in a two neck round bottom flask set with a condenser. Then 30% H_2O_2 (1.70 mL; 15 mmol) and H_2SO_4 (1 mL; 15 mmol) were added and left it for stirring under illumination of 100 W bulb. The mixture faded its colour from red to light yellow within 2.5h. After that the reaction mixture was washed with 40% sodium bisulphite. Evaporation of the solvent gave a crude mixture as an oil and the crude product was purified through column chromatography with ethyl acetate-hexane solvent mixture (1%) and characterised by ^1H NMR spectroscopy [1].

Synthesis of compound 3A: Theophylline (1.43 mmol; 257 mg) and K_2CO_3 (4.29 mmol; 594.17mg) were dissolved in DMF (8 mL), and then compound 2 (1.57 mmol; 500 mg) was added portion wise. The reaction mixture was stirred for 16h at room temperature and quenched by the addition of water, extracted with ethyl acetate (3 × 20 mL). The combined organic solution was washed with brine, dried over anhydrous Na_2SO_4, filtered, and concentrated under reduced pressure. The crude was purified by column chromatography using 30% ethyl acetate- hexane solvent mixture to afford 3A asa white solid [2].

Synthesis of compound 4A: Aldehyde (0.159 mmol), ammonium acetate (1.59 mmol; 122.5 mg) and compound 3A (0.159 mmol; 66.2 mg) was suspended in acetic acid (5 ml), and then the suspension was refluxed at 100^{0}C. After stirring for 4 h, the reaction mixture was diluted with ethyl acetate and washed with saturated $NaHCO_3$ and brine. The organic layer was concentrated *in vacuo.*

The crude product was purified by flash column chromatography with ethyl acetate-hexane solvent mixture[3].

Method B:

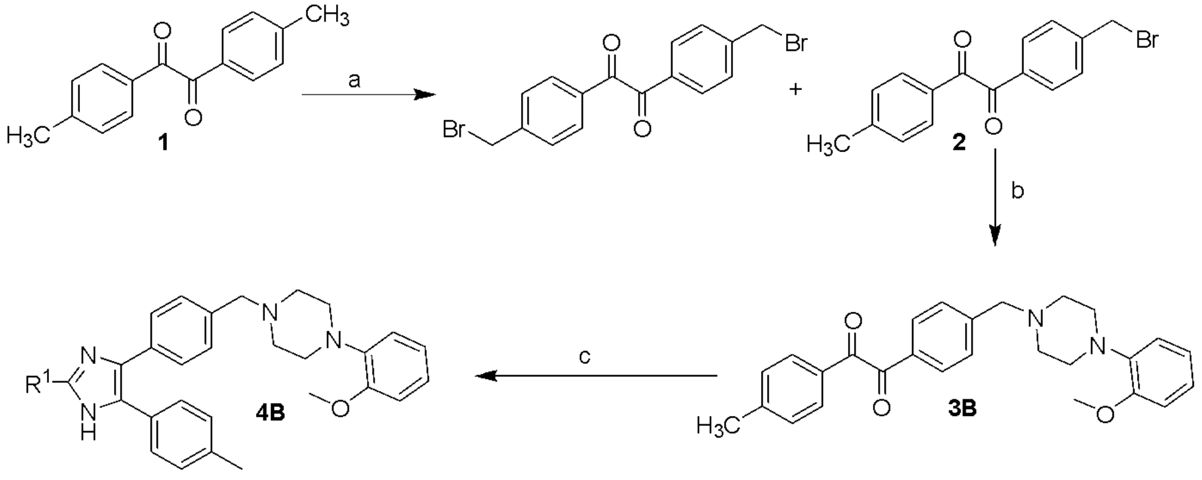

(a) NaBr, H_2O_2, H_2SO_4, $CHCl_3$; (b) 1-(2-methoxyphenyl)piperazine, K_2CO_3, DMF; (c) R_1CHO, NH_4OAc, AcOH, Reflux

Scheme S2. General scheme of cyclization with compound 3B

Synthesis of compound 2: Same as method A.

Synthesis of compound 3B: 1-(2-methoxyphenyl)piperazine (1.43 mmol; 274.93 mg) and K_2CO_3 (4.29 mmol;594.17 mg) were dissolved in DMF (8 mL), and then compound 2 (1.57 mmol; 500 mg) was added portion wise. The reaction mixture was stirred for 16h at room temperature and quenched by the addition of water, extracted with ethyl acetate (3 × 20 mL). The combined organic solution was washed with brine, dried over anhydrous Na_2SO_4, filtered, and concentrated under reduced pressure. The crude was purified by column chromatography using 30% ethyl acetate- hexanesolvent mixture to afford 3B as a white solid[2].

Synthesis of compound 4B: Aldehyde (0.159 mmol), ammonium acetate (1.59 mmol; 122.5 mg) and compound 3B (0.159 mmol; 63.5 mg) was suspended in acetic acid (5 ml), and then the suspension was refluxed at 100^{0}C. After stirring for 4 h, the reaction mixture was diluted with ethyl acetate and washed with saturated $NaHCO_3$ and brine. The organic layer was concentrated *in vacuo*. The crude product was purified by flash column chromatography with ethyl acetate –hexane solvent mixture [3].

Characterization of the molecules

1. **7-(4-(2-(4-chlorophenyl)-5-(p-tolyl)-1H-imidazol-4-yl) benzyl)-1, 3-dimethyl-3,7-dihydro-1H-purine-2,6-dione (SG-109C):** SG-109C was prepared from 4-chlorobenzaldehyde and compound 3A following method A. **(80 mg; 93.2%)**

   ^{1}H NMR: (300 MHz, DMSO-d6) δ ppm 2.43 (3H, s) 3.37 (3H, s) 3.58 (3H, s) 5.56 (2H, s) 7.25 (2H, d, *J*=7.91 Hz) 7.39 (2H, d, *J*= 7.91Hz) 7.64 (1 H, s) 7.88 (2H, d, *J*=8.29 Hz) 7.93(2H, d, J= 8.29Hz). ^{13}C NMR: (75 MHz, DMSO-d6) δ ppm 154.81, 151.40, 148.80, 144.71, 142.91, 133.14, 129.41, 127.18, 106.27, 49.15, 29.86, 27.95.

   ESI-MS Calculated for [$C_{30}H_{25}ClN_6O_2$] 536.1728. Found 536.2376 [M]

2. **1-(4-(2-(4-chlorophenyl)-5-(p-tolyl)-1H-imidazol-4-yl)benzyl)-4-(2-methoxyphenyl)piperazine (SG-138C) :** SG-138C was prepared from 4-chlorobenzaldehyde and compound 3B following method B.

   ^{1}H NMR (400 MHz, CHLOROFORM-*d*) δ ppm 2.29 - 2.34 (8 H, m) 2.63 - 2.73 (8 H, m) 3.00 - 3.11 (8 H, m) 3.41 - 3.45 (2 H, m) 3.59 - 3.62 (4 H, m) 3.79 - 3.87 (8 H, m) 6.83 - 6.93 (8 H, m) 6.96 - 7.03 (3 H, m) 7.09 - 7.13 (5 H, m) 7.22 - 7.40 (17 H, m) 7.44 - 7.51 (5 H, m) 7.80 - 7.88 (5 H, m)
   ^{13}C NMR (100 MHz, CHLOROFORM-*d*) δ ppm 152.30, 141.17, 137.46, 134.52, 129.90, 129.53, 129.42, 129.06, 128.54, 127.88, 127.80, 126.67, 123.21, 121.11, 118.44, 111.21, 62.46, 55.41, 52.92, 50.31, 29.15, 21.37

   ESI-MS Calculated for [$C_{34}H_{33}ClN_{4O}$] 548.2343. Found 547.2521 [M – H^+]

3. **1, 3-dimethyl-7-(4-(5-(p-tolyl)-1H,1'H-[2,2'-biimidazol]-4-yl)benzyl)-3,7-dihydro-1H-purine-2,6-dione (SG-139C) :** SG-139C was prepared from 1H-imidazole-2-carbaldehyde and compound 3A following method A.**(70 mg; 89%)**

   ^{1}H NMR (300 MHz, CHLOROFORM-*d*) δ ppm 2.31 (10 H, s) 3.38 (9 H, s) 3.48 (13 H, s) 3.57 (9 H, s) 5.44 (16 H, s) 6.84 (5 H, s) 7.06 (5 H, d, *J*=8.05 Hz) 7.11 - 7.31 (12 H, m) 7.42 (5 H, d, *J*=8.05 Hz) 7.53 (3 H, s)
   ^{13}C NMR (100 MHz, CHLOROFORM-*d*) δ ppm 155.35, 151.70, 148.84, 140.97, 138.78, 137.72, 134.14, 133.09, 129.35, 128.43, 128.10, 107.04, 50.00, 29.86, 28.07, 21.32.

ESI-MS Calculated for [$C_{27}H_{24}N_8O_2$] 492.2022. Found 492.2357[M], 493.2491 [M + H]$^+$

4. **7-(4-(2-(1H-indol-3-yl)-5-(p-tolyl)-1H-imidazol-4-yl)benzyl)-1,3-dimethyl-3,7-dihydro-1H-purine-2,6-dione (SG-141C)** : SG-141C was prepared from 1H-indole-3-carbaldehyde and compound 3A following method A. **(70 mg; 81.3%)**

^{1}H NMR (300 MHz, DMSO-d6) δ ppm 2.00 (6 H, s) 2.34 (5 H, s) 3.36 (5 H, s) 3.48 (6 H, s) 3.55 (5 H, s) 5.41 (4 H, s) 6.87 - 7.22 (9 H, m) 7.22 - 7.40 (8 H, m) 7.42 - 7.58 (4 H, m) 7.63 (2 H, br. s.) 8.00 (2 H, d, J=8.05 Hz) 9.27 (1 H, br. s.)
^{13}C NMR (100 MHz, DMSO-d6) δ ppm 154.95, 151.54, 148.89, 142.95, 136.72, 129.68, 128.03, 125.47, 124.24, 122.40, 121.88, 120.22, 112.11, 107.21, 106.41, 49.34, 29.95, 28.07, 21.28

ESI-MS Calculated for [C32H27N7O2] 541.2226. Found 541.2314[M], 542.2396 [M + H]+

5. **7-(4-(2-(5-methoxy-1H-indol-3-yl)-5-(p-tolyl)-1H-imidazol-4-yl)benzyl)-1,3-dimethyl-3,7-dihydro-1H-purine-2,6-dione (SG-143C) :** SG-143C was prepared from 5-methoxy-1H-indole-3-carbaldehyde and compound 3A following method A.(75 mg; 82.6%)

^{1}H NMR (300 MHz, DMSO-d_6) δ ppm 1.90 (1 H, s) 2.30 (1 H, s) 3.19 (1 H, s) 3.40 (2 H, s) 3.79 (90 H, br. s.) 5.46 (1 H, s) 7.35 (1 H, s) 7.92 (1 H, d, *J*=10.98 Hz)
^{13}C NMR (100 MHz, DMSO-d_6) δ ppm 172.83, 154.94, 154.36, 151.54, 148.86, 142.95, 131.86, 128.04, 125.97, 124.85, 112.76, 112.30, 106.93, 106.42, 103.68, 55.79, 29.96, 28.08, 21.27

ESI-MS Calculated for [$C_{33}H_{29}N_7O_3$] 571.2332. Found 572.4004 [M + H]$^+$

6. **1,3-dimethyl-7-(4-(2-(pyridin-4-yl)-5-(p-tolyl)-1H-imidazol-4-yl)benzyl)-3,7-dihydro-1H-purine-2,6-dione (SG-144C) :** SG-144C was prepared from isonicotinaldehyde and compound 3A following method A. **(75mg; 93.6%)**

^{1}H NMR (400 MHz, CHLOROFORM-*d*) δ ppm 2.22 - 2.26 (10 H, m) 3.24 - 3.30 (10 H, m) 3.38 - 3.39 (1 H, m) 3.45 - 3.51 (10 H, m) 5.36 - 5.40 (6 H, m) 6.98 - 7.03 (6 H, m) 7.10 - 7.15 (7 H, m) 7.19 - 7.26 (9 H, m) 7.45 - 7.53 (14 H, m) 7.79 - 7.82 (5 H, m) 8.39 - 8.45 (5 H, m) 8.59 - 8.63 (6 H, m)
^{13}C NMR (100 MHz, CHLOROFORM-*d*) δ ppm 155.34, 151.68, 150.24, 149.76, 148.89, 144.44, 143.45, 141.18, 137.68, 129.37, 128.36, 128.23, 128.11, 128.04, 128.00, 124.06, 119.58, 107.05, 50.47, 29.89, 28.07, 21.31

ESI-MS Calculated for [$C_{29}H_{25}N_7O_2$] 503.2070. Found 504.2822 [M + H]$^+$

7. **7-(4-(2-(4-hydroxyphenyl)-5-(p-tolyl)-1H-imidazol-4-yl)benzyl)-1,3-dimethyl-3,7-dihydro-1H-purine-2,6-dione (SG-145C) :** SG-145C was prepared from 4-hydroxybenzaldehyde and compound 3A following method A. **(76 mg; 92.1%)**

^{1}H NMR (300 MHz, DMSO-d_6) δ ppm 2.28 (3 H, br. s.) 3.02 - 3.03 (1 H, m) 3.18 (4 H, s) 3.39 (7 H, s) 5.45 (20 H, br. s.) 6.81 - 6.84 (20 H, m) 7.31 (2 H, d, *J*=7.68 Hz) 7.41 (1 H, br. s.) 7.83 (2 H, d, *J*=8.42 Hz)
^{13}C NMR (100 MHz, DMSO-d_6) δ ppm 158.1, 154.95, 151.57, 148.86, 146.60, 142.96, 129.57, 128.02, 127.42, 121.90, 115.94, 106.43, 56.65, 29.97, 28.08, 21.23.

ESI-MS Calculated for [$C_{30}H_{26}N_6O_3$] 518.2066. Found 518.3709.

8. 1,3-dimethyl-7-(4-(2-(4-morpholinophenyl)-5-(p-tolyl)-1H-imidazol-4-yl)benzyl)-3,7-dihydro-1H-purine-2,6-dione (SG-146C) : SG-146C was prepared from 4-morpholinobenzaldehyde and compound 3A following method A.**(80 mg; 85.6%)**

^{1}H NMR (300 MHz, CHLOROFORM-*d*) δ ppm 2.33 (10 H, s) 2.96 - 3.29 (10 H, m) 3.37 (16 H, s) 3.60 - 3.94 (11 H, m) 5.43 (5 H, s) 6.90 (5 H, d, *J*=8.78 Hz) 7.10 (5 H, s) 7.17 (5 H, s) 7.30 (14 H, d, *J*=7.68 Hz) 7.80 (8 H, d, *J*=8.42 Hz)
^{13}C NMR (100 MHz, CHLOROFORM-*d*) δ ppm 155.38, 151.72, 148.91, 143.60, 141.09, 129.40, 128.18, 128.00, 126.55, 115.21, 107.10, 100.00, 66.82, 48.67, 29.88, 28.10

ESI-MS Calculated for [$C_{34}H_{33}N_7O_3$] 587.2645. Found 587.3797 [M], 588.3823 $[M + H]^+$

9. 7-(4-(2-(3,4-dichlorophenyl)-5-(p-tolyl)-1H-imidazol-4-yl)benzyl)-1,3-dimethyl-3,7-dihydro-1H-purine-2,6-dione (SG-160C): SG-160C was prepared from 3,4 dichlorobenzaldehyde and compound 3A following method A. **(86 mg; 94.6%)**

^{1}H NMR (400 MHz, CHLOROFORM-d) δ ppm 2.30 (11 H, s) 3.30 - 3.33 (11 H, m) 3.44 - 3.46 (5 H, m) 3.54 - 3.56 (11 H, m) 5.43 (7 H, s) 7.04 - 7.07 (7 H, m) 7.12 - 7.16 (7 H, m) 7.23 - 7.28 (11 H, m) 7.40 - 7.48 (10 H, m) 7.55 - 7.57 (3 H, m) 7.69 - 7.72 (4 H, m) 7.92 (3 H, d, J=2.07 Hz)

^{13}C NMR (400 MHz, DMSO-d6) δ ppm 155.47, 151.68, 149.08, 143.90, 141.25, 133.07,132.59, 130.87, 129.84, 129.48, 128.20, 127.98, 127.87, 127.03, 124.49, 107.09, 50.17,29.93, 28.12, 21.33.

ESI-MS Calculated for [C 30 H 24 Cl 2 N 6 O 2] 570.1338. found 570.2178 [M], 571.2214 [M + H] +

10. 7-(4-(2-(2-chlorophenyl)-5-(p-tolyl)-1H-imidazol-4-yl)benzyl)-1,3-dimethyl-3,7-dihydro-1H-purine-2,6-dione (SG-162C): SG-162C was prepared from 2-chlorobenzaldehyde and compound 3A following method A. **(79 mg; 92.12%)**

^{1}H NMR: (400 MHz, CHLOROFORM-*d*) δ ppm 2.37 (3H, S) 3.39 (3H, S) 3.56 (3H, S) 5.48 (2H, S) 7.16 (2H, d, J= 8Hz) 7.24 (3H, d, J= 8Hz) 7.29-7.40 (4H, m) 7.42 (1H, s) 7.54-7.62 (2H, bs) 8.39 (1H, d, 8Hz)
^{13}C NMR: (100 MHz, CHLOROFORM-*d*) δ ppm 155.19, 151.58, 148.73, 143.14, 140.79, 130.78, 130.34, 129.71, 129.56, 129.48, 128.15, 128.05, 127.79, 127.38, 106.94, 50.01, 29.68, 27.91, 21.20.

ESI-MS Calculated for [$C_{30}H_{25}ClN_6O_2$] 536.1728. Found 537.1802 [M + H]$^+$

Table S1-8: Library of designed 162 molecules

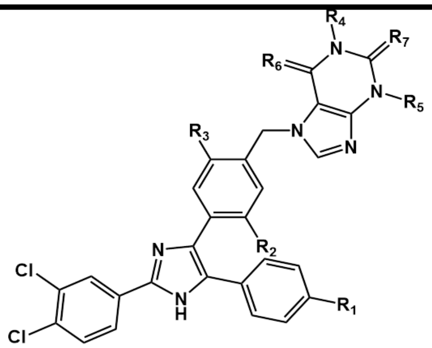

Sl no.	R1	R2	R3	R4	R5	R6	R7
SG-1C	-CCH$_3$	H	H	-CH$_3$	-CH$_3$	O	O
SG-2C	-C$_2$H$_5$	H	H	-CH$_3$	-CH$_3$	O	O
SG-3C	H	H	H	-CH$_3$	-CH$_3$	O	O
SG-4C	-CH$_3$	Cl	H	-CH$_3$	-CH$_3$	O	O
SG-5C	-CH$_3$	Br	H	-CH$_3$	-CH$_3$	O	O
SG-6C	-CH$_3$	F	H	-CH$_3$	-CH$_3$	O	O
SG-7C	-CH$_3$	H	Cl	-CH$_3$	-CH$_3$	O	O
SG-8C	-CH$_3$	H	F	-CH$_3$	-CH$_3$	O	O
SG-9C	-CH$_3$	H	Br	-CH$_3$	-CH$_3$	O	O
SG-10C	-CH$_3$	H	H	H	-CH$_3$	O	O
SG-11C	-CH$_3$	H	Br	-CH$_3$	H	O	O
SG-12C	-CH$_3$	H	Br	H	H	O	O
SG-13C	-CH$_3$	H	Br	-CH$_3$	H	Cl$_2$	O
SG-14C	-CH$_3$	H	Br	-CH$_3$	H	O	Cl$_2$

Table: S1

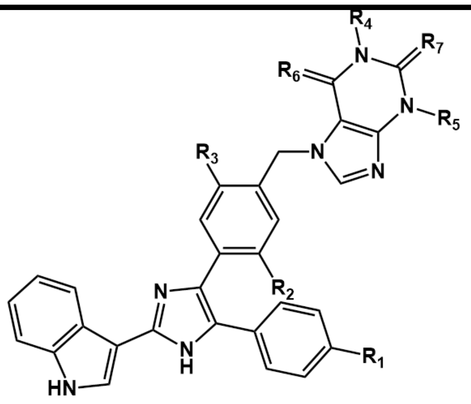

Sl no.	R1	R2	R3	R4	R5	R6	R7
SG-15C	$-CCH_3$	H	H	$-CH_3$	$-CH_3$	O	O
SG-16C	$-C_2H_5$	H	H	$-CH_3$	$-CH_3$	O	O
SG-17C	H	H	H	$-CH_3$	$-CH_3$	O	O
SG-18C	$-CH_3$	Cl	H	$-CH_3$	$-CH_3$	O	O
SG-19C	$-CH_3$	Br	H	$-CH_3$	$-CH_3$	O	O
SG-20C	$-CH_3$	F	H	$-CH_3$	$-CH_3$	O	O
SG-21C	$-CH_3$	H	Cl	$-CH_3$	$-CH_3$	O	O
SG-22C	$-CH_3$	H	F	$-CH_3$	$-CH_3$	O	O
SG-23C	$-CH_3$	H	Br	$-CH_3$	$-CH_3$	O	O
SG-24C	$-CH_3$	H	H	H	$-CH_3$	O	O
SG-25C	$-CH_3$	H	Br	$-CH_3$	H	O	O
SG-26C	$-CH_3$	H	Br	H	H	O	O
SG-27C	$-CH_3$	H	Br	$-CH_3$	H	Cl_2	O
SG-28C	$-CH_3$	H	Br	$-CH_3$	H	O	Cl_2
SG-29C	$-CH_3$	H	Br	$-CH_3$	H	O	$(CH_3)_2$
SG-30C	$-CH_3$	H	Br	$-CH_3$	H	$(CH_3)_2$	Cl_2

Table: S2

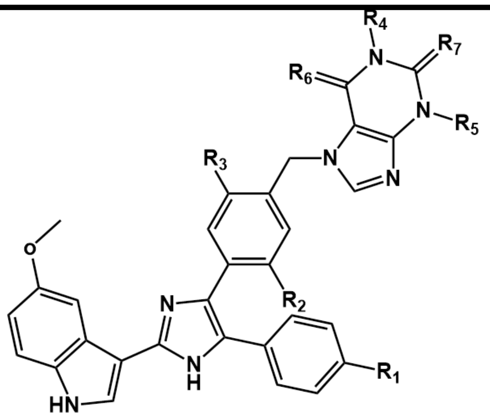

Sl no.	R1	R2	R3	R4	R5	R6	R7
SG-31C	$-CCH_3$	H	H	$-CH_3$	$-CH_3$	O	O
SG-32C	$-C_2H_5$	H	H	$-CH_3$	$-CH_3$	O	O
SG-33C	H	H	H	$-CH_3$	$-CH_3$	O	O
SG-34C	$-CH_3$	Cl	H	$-CH_3$	$-CH_3$	O	O
SG-35C	$-CH_3$	Br	H	$-CH_3$	$-CH_3$	O	O
SG-36C	$-CH_3$	F	H	$-CH_3$	$-CH_3$	O	O
SG-37C	$-CH_3$	H	Cl	$-CH_3$	$-CH_3$	O	O
SG-38C	$-CH_3$	H	F	$-CH_3$	$-CH_3$	O	O
SG-39C	$-CH_3$	H	Br	$-CH_3$	$-CH_3$	O	O
SG-40C	$-CH_3$	H	H	H	$-CH_3$	O	O
SG-41C	$-CH_3$	H	Br	$-CH_3$	H	O	O
SG-42C	$-CH_3$	H	Br	H	H	O	O
SG-43C	$-CH_3$	H	Br	$-CH_3$	H	Cl_2	O
SG-44C	$-CH_3$	H	Br	$-CH_3$	H	O	Cl_2
SG-45C	$-CH_3$	H	Br	$-CH_3$	H	O	$(CH_3)_2$
SG-46C	$-CH_3$	H	Br	$-CH_3$	H	$(CH_3)_2$	Cl_2

Table: S3

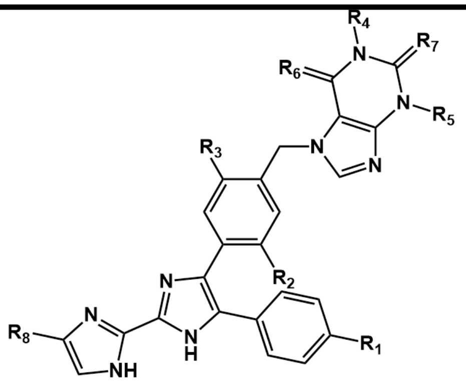

Sl no.	R1	R2	R3	R4	R5	R6	R7	R8
SG-47C	$-CCH_3$	H	H	$-CH_3$	$-CH_3$	O	O	H
SG-48C	$-C_2H_5$	H	H	$-CH_3$	$-CH_3$	O	O	H
SG-49C	H	H	H	$-CH_3$	$-CH_3$	O	O	H
SG-50C	$-CH_3$	Cl	H	$-CH_3$	$-CH_3$	O	O	H
SG-51C	$-CH_3$	Br	H	$-CH_3$	$-CH_3$	O	O	H
SG-52C	$-CH_3$	F	H	$-CH_3$	$-CH_3$	O	O	H
SG-53C	$-CH_3$	H	Cl	$-CH_3$	$-CH_3$	O	O	H
SG-54C	$-CH_3$	H	F	$-CH_3$	$-CH_3$	O	O	H
SG-56C	$-CH_3$	H	Br	$-CH_3$	$-CH_3$	O	O	H
SG-57C	$-CH_3$	H	H	H	$-CH_3$	O	O	H
SG-58C	$-CH_3$	H	Br	$-CH_3$	H	O	O	H
SG-59C	$-CH_3$	H	Br	H	H	O	O	H
SG-60C	$-CH_3$	H	Br	$-CH_3$	H	Cl_2	O	H
SG-61C	$-CH_3$	H	Br	$-CH_3$	H	O	Cl_2	H
SG-62C	$-CH_3$	H	Br	$-CH_3$	H	O	$(CH_3)_2$	H
SG-63C	$-CH_3$	H	Br	$-CH_3$	H	$(CH_3)_2$	O	H
SG-64C	$-CH_3$	H	Br	$-CH_3$	H	$(CH_3)_2$	O	-Cl
SG-65C	$-CH_3$	H	Br	$-CH_3$	H	$(CH_3)_2$	O	-OH

Table: S4

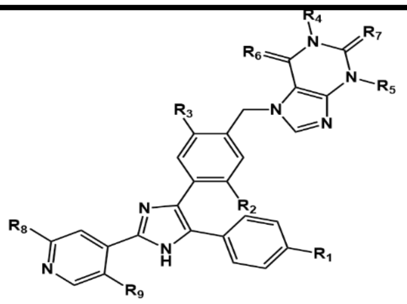

Sl no.	R1	R2	R3	R4	R5	R6	R7	R8	R9
SG-66C	-CCH_3	H	H	-CH_3	-CH_3	O	O	H	H
SG-67C	-C_2H_5	H	H	-CH_3	-CH_3	O	O	H	H
SG-68C	H	H	H	-CH_3	-CH_3	O	O	H	H
SG-69C	-CH_3	Cl	H	-CH_3	-CH_3	O	O	H	H
SG-70C	-CH_3	Br	H	-CH_3	-CH_3	O	O	H	H
SG-71C	-CH_3	F	H	-CH_3	-CH_3	O	O	H	H
SG-72C	-CH_3	H	Cl	-CH_3	-CH_3	O	O	H	H
SG-73C	-CH_3	H	F	-CH_3	-CH_3	O	O	H	H
SG-74C	-CH_3	H	Br	-CH_3	-CH_3	O	O	H	H
SG-75C	-CH_3	H	H	H	-CH_3	O	O	H	H
SG-76C	-CH_3	H	H	-CH_3	H	O	O	H	H
SG-77C	-CH_3	H	H	H	H	O	O	H	H
SG-78C	-CH_3	H	H	-CH_3	H	Cl_2	O	H	H
SG-79C	-CH_3	H	H	-CH_3	H	O	Cl_2	H	H
SG-80C	-CH_3	H	H	-CH_3	H	O	$(CH_3)_2$	H	H
SG-81C	-CH_3	H	H	-CH_3	H	$(CH_3)_2$	O	H	H
SG-82C	-CH_3	H	H	-CH_3	H	$(CH_3)_2$	O	Cl	H
SG-83C	-CH_3	H	H	-CH_3	H	$(CH_3)_2$	O	-OH	H
SG-84C	-CH_3	H	H	-CH_3	H	$(CH_3)_2$	O	F	H
SG-85C	-CH_3	H	H	-CH_3	H	$(CH_3)_2$	O	Br	H
SG-86C	-CH_3	H	H	-CH_3	H	$(CH_3)_2$	O	I	H
SG-87C	-CH_3	H	H	-CH_3	H	$(CH_3)_2$	O	--CH_3	H
SG-88C	-CH_3	H	H	-CH_3	H	$(CH_3)_2$	O	H	Cl
SG-89C	-CH_3	H	H	-CH_3	H	$(CH_3)_2$	O	H	-OH
SG-90C	-CH_3	H	H	-CH_3	H	$(CH_3)_2$	O	H	F
SG-110C	-CH_3	H	H	-CH_3	H	$(CH_3)_2$	O	H	Br
SG-111C	-CH_3	H	H	-CH_3	H	$(CH_3)_2$	O	H	I
SG-113C	-CH_3	H	H	-CH_3	H	$(CH_3)_2$	O	H	-CH_3

Table: S5

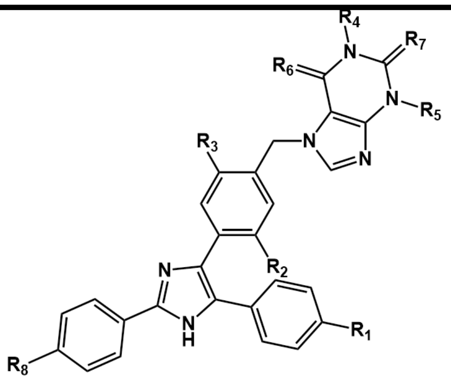

Sl no.	R1	R2	R3	R4	R5	R6	R7	R8
SG-91C	$-CCH_3$	H	H	$-CH_3$	$-CH_3$	O	O	H
SG-92C	$-C_2H_5$	H	H	$-CH_3$	$-CH_3$	O	O	H
SG-93C	H	H	H	$-CH_3$	$-CH_3$	O	O	H
SG-94C	$-CH_3$	Cl	H	$-CH_3$	$-CH_3$	O	O	H
SG-95C	$-CH_3$	Br	H	$-CH_3$	$-CH_3$	O	O	H
SG-96C	$-CH_3$	F	H	$-CH_3$	$-CH_3$	O	O	H
SG-97C	$-CH_3$	H	Cl	$-CH_3$	$-CH_3$	O	O	H
SG-98C	$-CH_3$	H	F	$-CH_3$	$-CH_3$	O	O	H
SG-99C	$-CH_3$	H	Br	$-CH_3$	$-CH_3$	O	O	H
SG-100C	$-CH_3$	H	H	H	$-CH_3$	O	O	H
SG-101C	$-CH_3$	H	Br	$-CH_3$	H	O	O	H
SG-102C	$-CH_3$	H	Br	H	H	O	O	H
SG-103C	$-CH_3$	H	Br	$-CH_3$	H	Cl_2	O	H
SG-104C	$-CH_3$	H	Br	$-CH_3$	H	O	Cl_2	H
SG-105C	$-CH_3$	H	Br	$-CH_3$	H	O	$(CH_3)_2$	H
SG-106C	$-CH_3$	H	Br	$-CH_3$	H	$(CH_3)_2$	O	H
SG-107C	$-CH_3$	H	Br	$-CH_3$	H	$(CH_3)_2$	O	-Cl
SG-108C	$-CH_3$	H	H	$-CH_3$	$-CH_3$	O	O	F
SG-109C	$-CH_3$	H	H	$-CH_3$	$-CH_3$	O	O	Cl

Table: S6

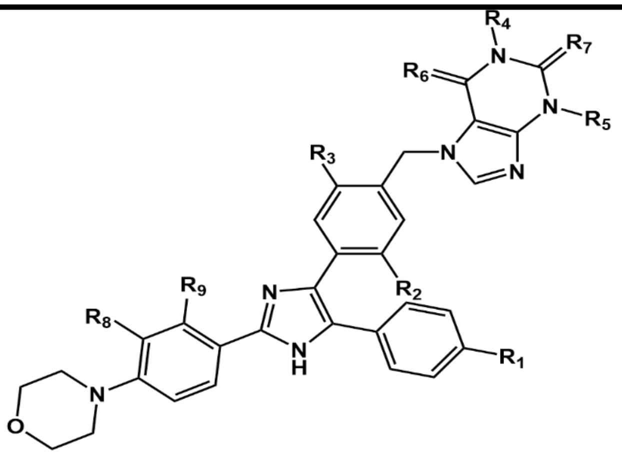

Sl no.	R1	R2	R3	R4	R5	R6	R7	R8	R9
SG-114C	$-CCH_3$	H	H	$-CH_3$	$-CH_3$	O	O	H	H
SG-115C	$-C_2H_5$	H	H	$-CH_3$	$-CH_3$	O	O	H	H
SG-116C	H	H	H	$-CH_3$	$-CH_3$	O	O	H	H
SG-117C	$-CH_3$	Cl	H	$-CH_3$	$-CH_3$	O	O	H	H
SG-118C	$-CH_3$	Br	H	$-CH_3$	$-CH_3$	O	O	H	H
SG-119C	$-CH_3$	F	H	$-CH_3$	$-CH_3$	O	O	H	H
SG-121C	$-CH_3$	H	Cl	$-CH_3$	$-CH_3$	O	O	H	H
SG-122C	$-CH_3$	H	F	$-CH_3$	$-CH_3$	O	O	H	H
SG-123C	$-CH_3$	H	Br	$-CH_3$	$-CH_3$	O	O	H	H
SG-124C	$-CH_3$	H	H	H	$-CH_3$	O	O	H	H
SG-125C	$-CH_3$	H	H	$-CH_3$	H	O	O	H	H
SG-126C	$-CH_3$	H	H	H	H	O	O	H	H
SG-127C	$-CH_3$	H	H	$-CH_3$	H	Cl_2	O	H	H
SG-129C	$-CH_3$	H	H	$-CH_3$	H	O	Cl_2	H	H
SG-130C	$-CCH_3$	H	H	$-CH_3$	$-CH_3$	O	O	Cl	H
SG-131C	$-CCH_3$	H	H	$-CH_3$	$-CH_3$	O	O	H	Cl
SG-132C	$-CCH_3$	H	H	$-CH_3$	$-CH_3$	O	O	Br	H
SG-133C	$-CH_3$	H	H	$-CH_3$	$-CH_3$	O	O	H	Br
SG-134C	$-CH_3$	H	H	$-CH_3$	$-CH_3$	O	O	F	H
SG-135C	$-CH_3$	H	H	$-CH_3$	H	O	O	H	F

Table: S7

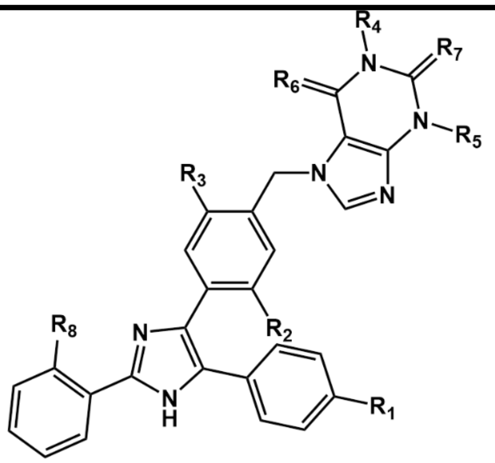

Sl no.	R1	R2	R3	R4	R5	R6	R7	R8
SG-136C	$-CCH_3$	H	H	$-CH_3$	$-CH_3$	O	O	H
SG-137C	$-C_2H_5$	H	H	$-CH_3$	$-CH_3$	O	O	H
SG-140C	H	H	H	$-CH_3$	$-CH_3$	O	O	H
SG-142C	$-CH_3$	Cl	H	$-CH_3$	$-CH_3$	O	O	H
SG-147C	$-CH_3$	Br	H	$-CH_3$	$-CH_3$	O	O	H
SG-148C	$-CH_3$	F	H	$-CH_3$	$-CH_3$	O	O	H
SG-149C	$-CH_3$	H	Cl	$-CH_3$	$-CH_3$	O	O	H
SG-150C	$-CH_3$	H	F	$-CH_3$	$-CH_3$	O	O	H
SG-151C	$-CH_3$	H	Br	$-CH_3$	$-CH_3$	O	O	H
SG-152C	$-CH_3$	H	H	H	$-CH_3$	O	O	H
SG-153C	$-CH_3$	H	Br	$-CH_3$	H	O	O	H
SG-154C	$-CH_3$	H	Br	H	H	O	O	H
SG-155C	$-CH_3$	H	Br	$-CH_3$	H	Cl_2	O	H
SG-156C	$-CH_3$	H	Br	$-CH_3$	H	O	Cl_2	H
SG-157C	$-CH_3$	H	Br	$-CH_3$	H	O	$(CH_3)_2$	H
SG-158C	$-CH_3$	H	Br	$-CH_3$	H	$(CH_3)_2$	O	H
SG-159C	$-CH_3$	H	Br	$-CH_3$	H	$(CH_3)_2$	O	-Cl
SG-161C	$-CH_3$	H	H	$-CH_3$	$-CH_3$	O	O	F

Table: S8

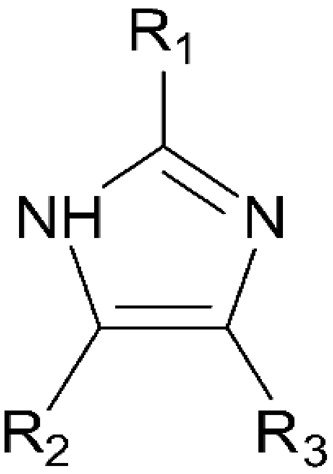

Imidazole Core and substitution positions

Sl No.	Compound	R_1 group	R_2 group	R_3 group
1	SG-109C	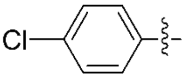	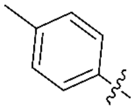	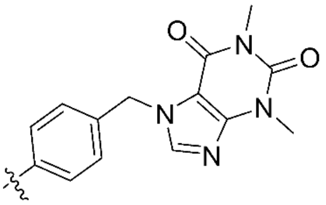
2	SG-138C	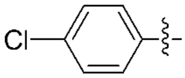	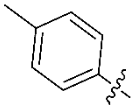	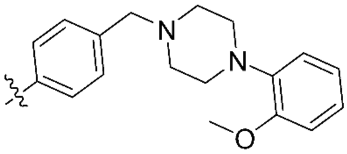
3	SG-139C	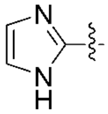	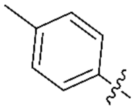	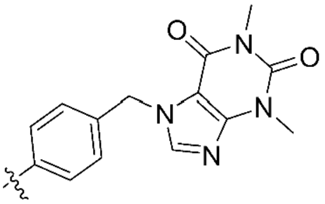
4	SG-141C	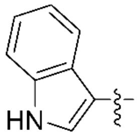	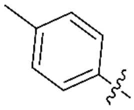	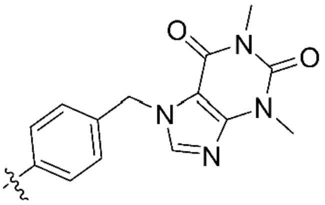
5	SG-143C	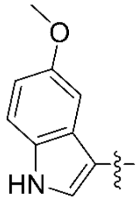	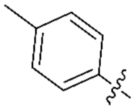	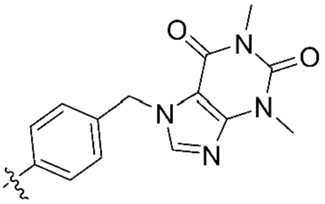
6	SG-144C	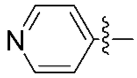	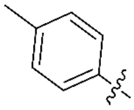	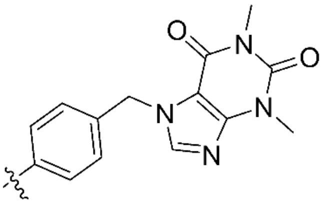

**7**	SG-145C	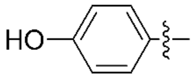	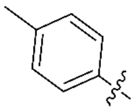	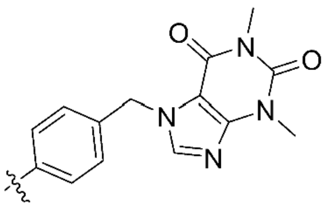
**8**	SG-146C	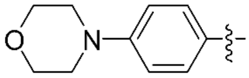	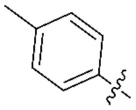	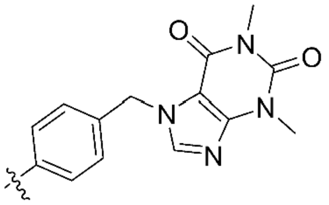
**9**	SG-160C	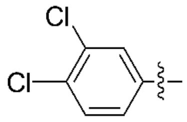	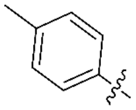	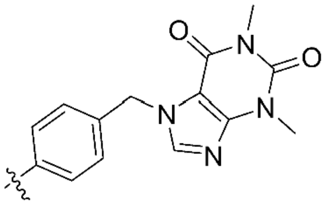
**10**	SG-162C	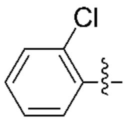	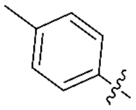	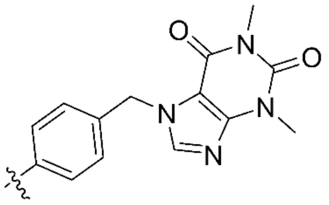

Table S9. Library of substituted biosimilars imidazole molecule

Table: S10
Crystal data and structure refinement for SG-145C

Identification code	SG_145C_0m_a
Empirical formula	$C_{34}H_{32}N_8O_3$
Formula weight	600.67
Temperature/K	101.0
Crystal system	monoclinic
Space group	$P2_1/c$
a/Å	12.8005(7)
b/Å	21.3557(12)
c/Å	11.5984(6)
α/°	90
β/°	96.507(3)
γ/°	90
Volume/Å^3	3150.2(3)
Z	4

ρ_{calc}g/cm^3	1.267
μ/mm^1	0.682
F(000)	1264.0
Crystal size/mm^3	0.24 × 0.22 × 0.15
Radiation	CuKα (λ = 1.54184)
2Θ range for data collection/°	6.95 to 130.196
Index ranges	-14 ≤ h ≤ 15, -25 ≤ k ≤ 24, -13 ≤ l ≤ 13
Reflections collected	46498
Independent reflections	5335 [R_{int} = 0.0964, R_{sigma} = 0.0703]
Data/restraints/parameters	5335/0/413
Goodness-of-fit on F^2	1.084
Final R indexes [I>=2σ (I)]	R_1 = 0.0855, wR_2 = 0.2220
Final R indexes [all data]	R_1 = 0.1100, wR_2 = 0.2425
Largest diff. peak/hole / e Å^{-3}	0.69/-0.39
CCDC	2132568

Figures

In Silico Data

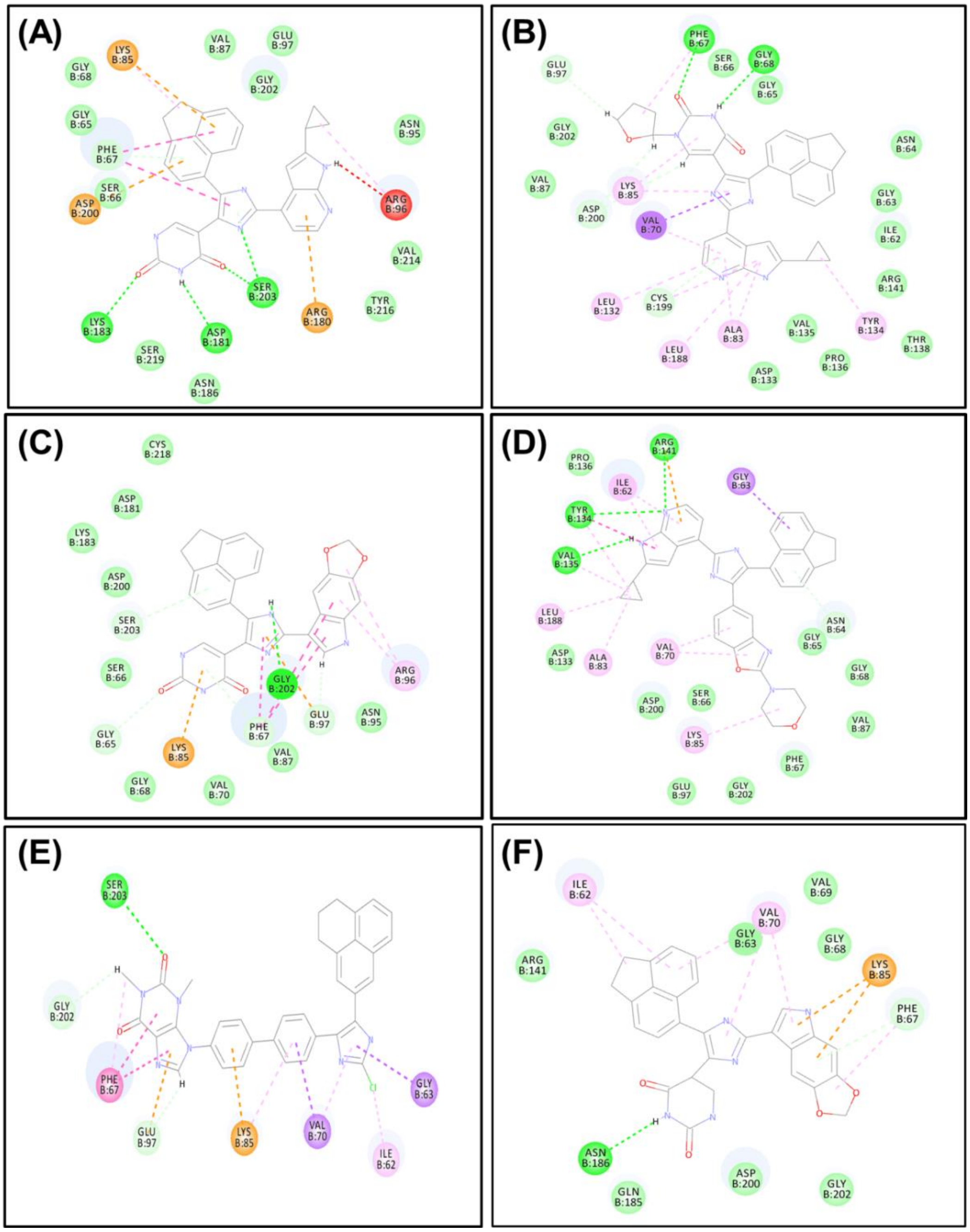

Fig. S1

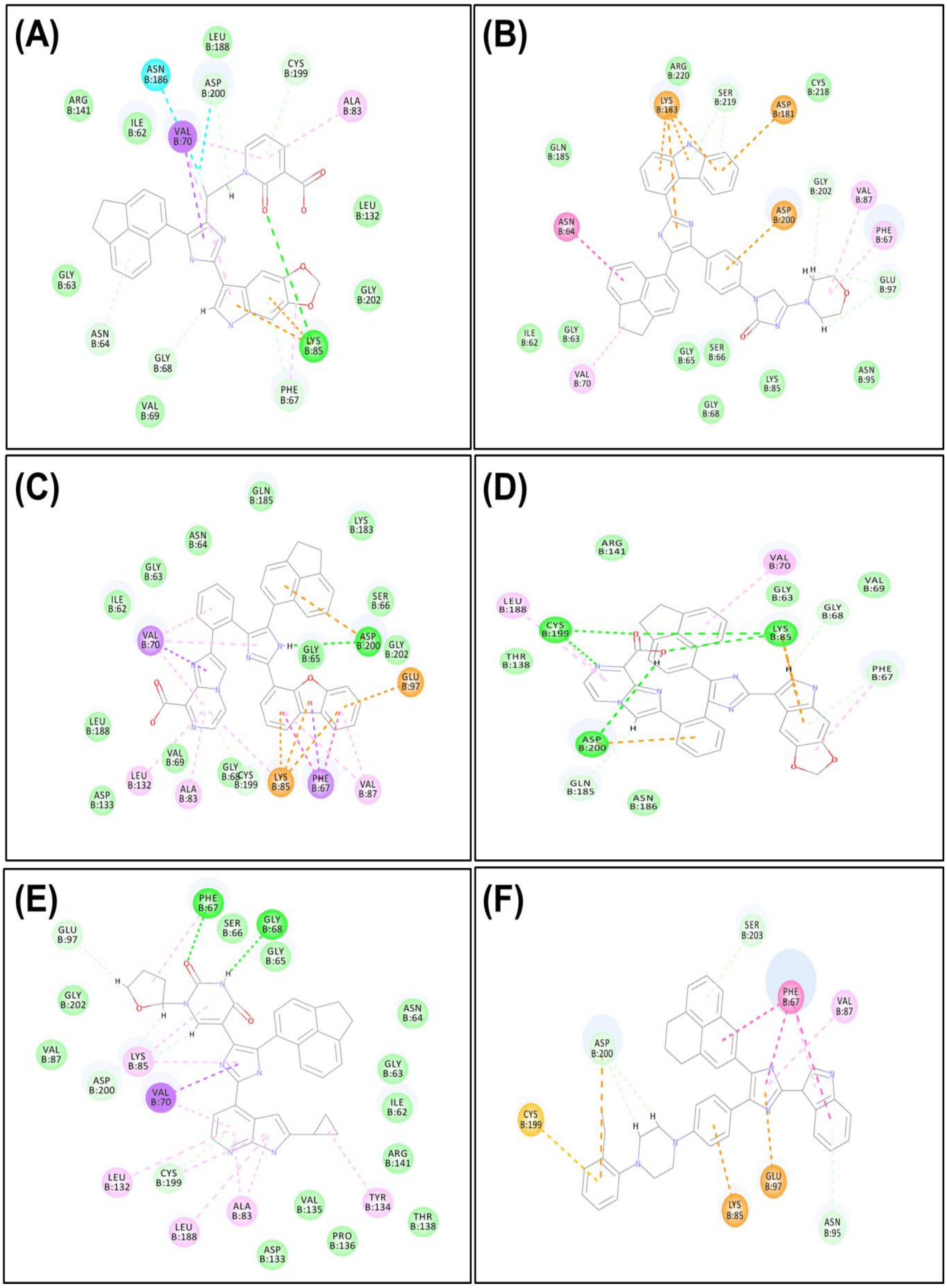

Fig. S2

Figure S1 & S2 showing different scaffold growth on imidazole core and their interaction with GSK-3β core.

Figure S3-S10 showing pharmacophore mapping of best 50molecules from 162 molecular library.

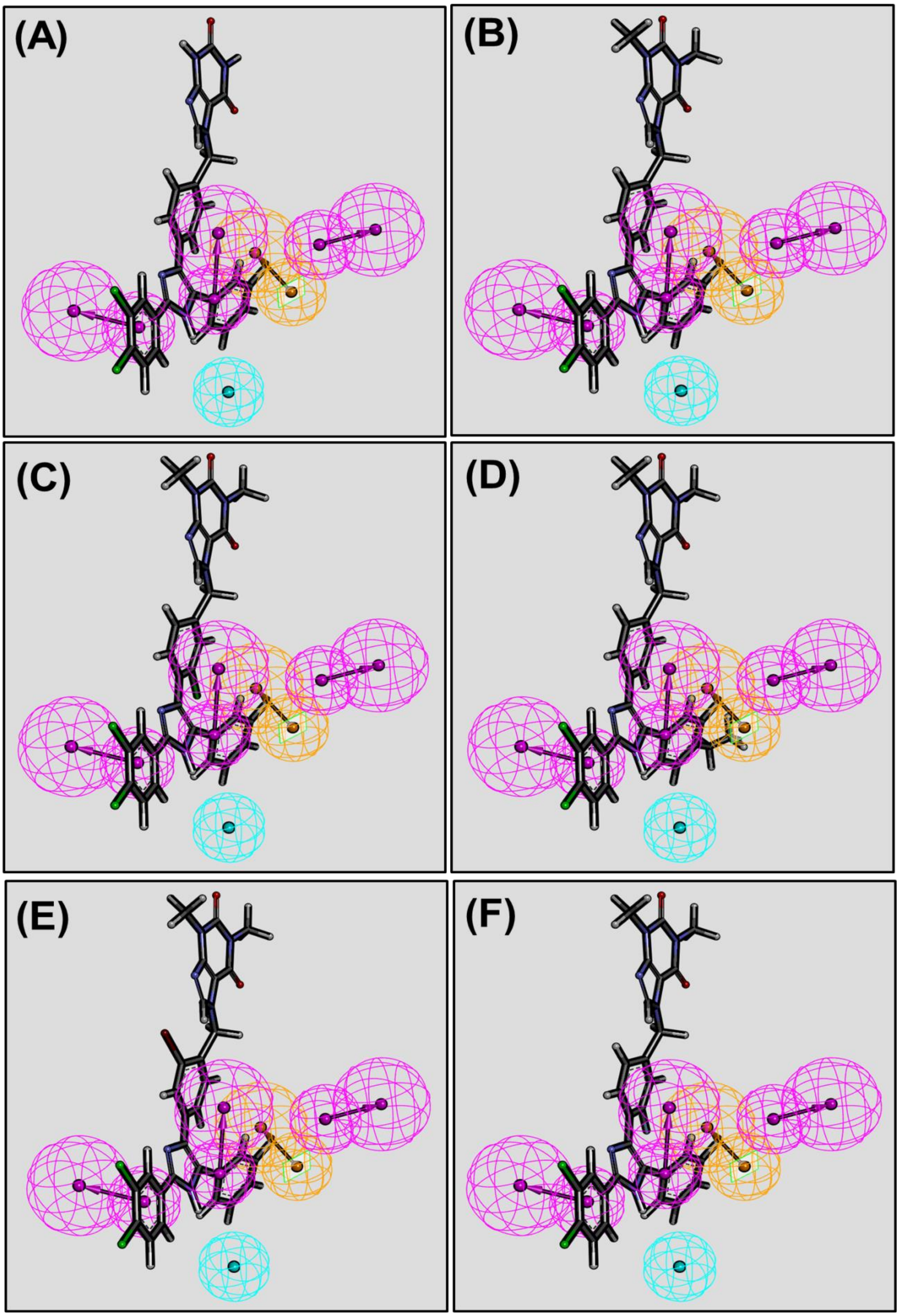

Fig. S3

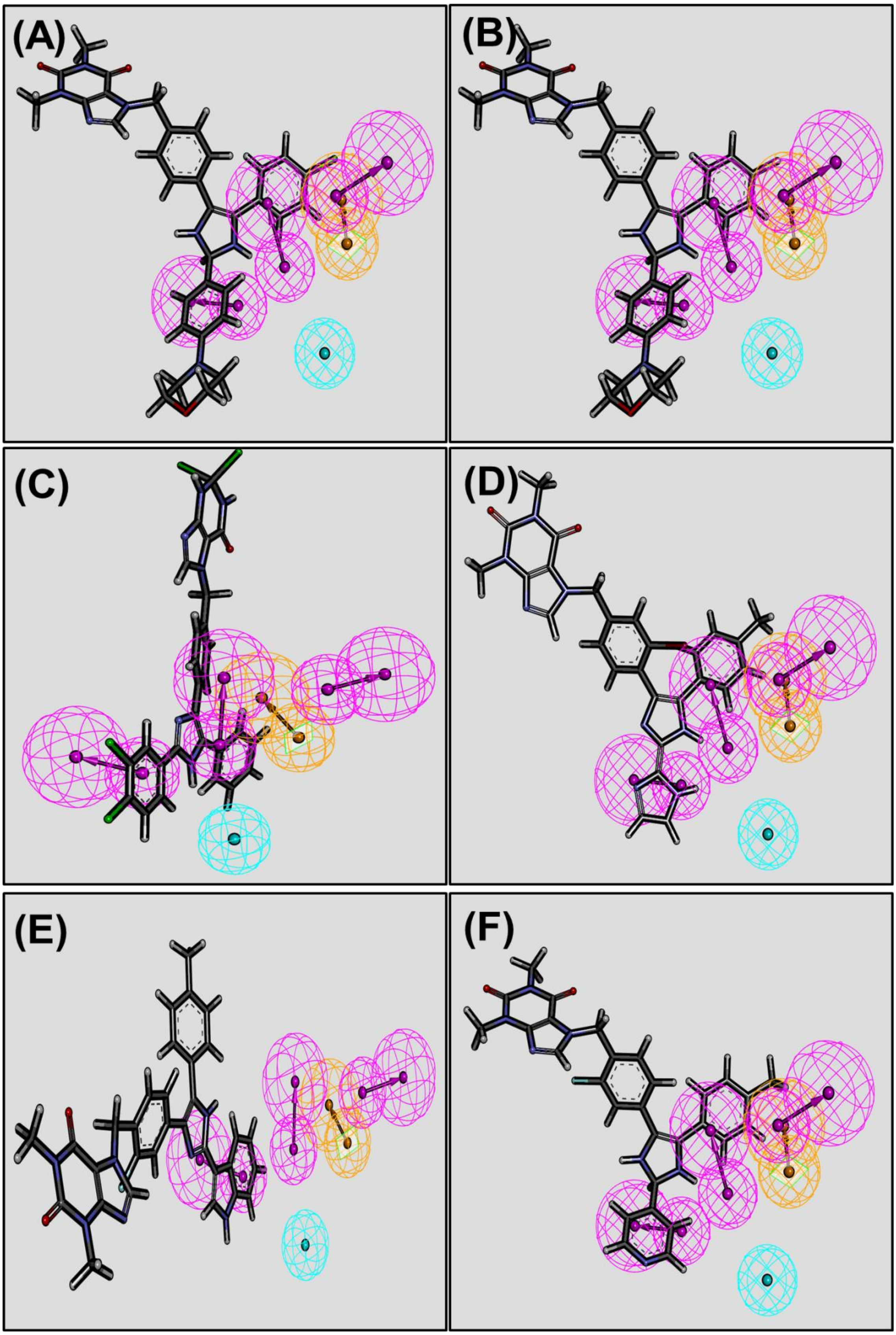

Fig. S4

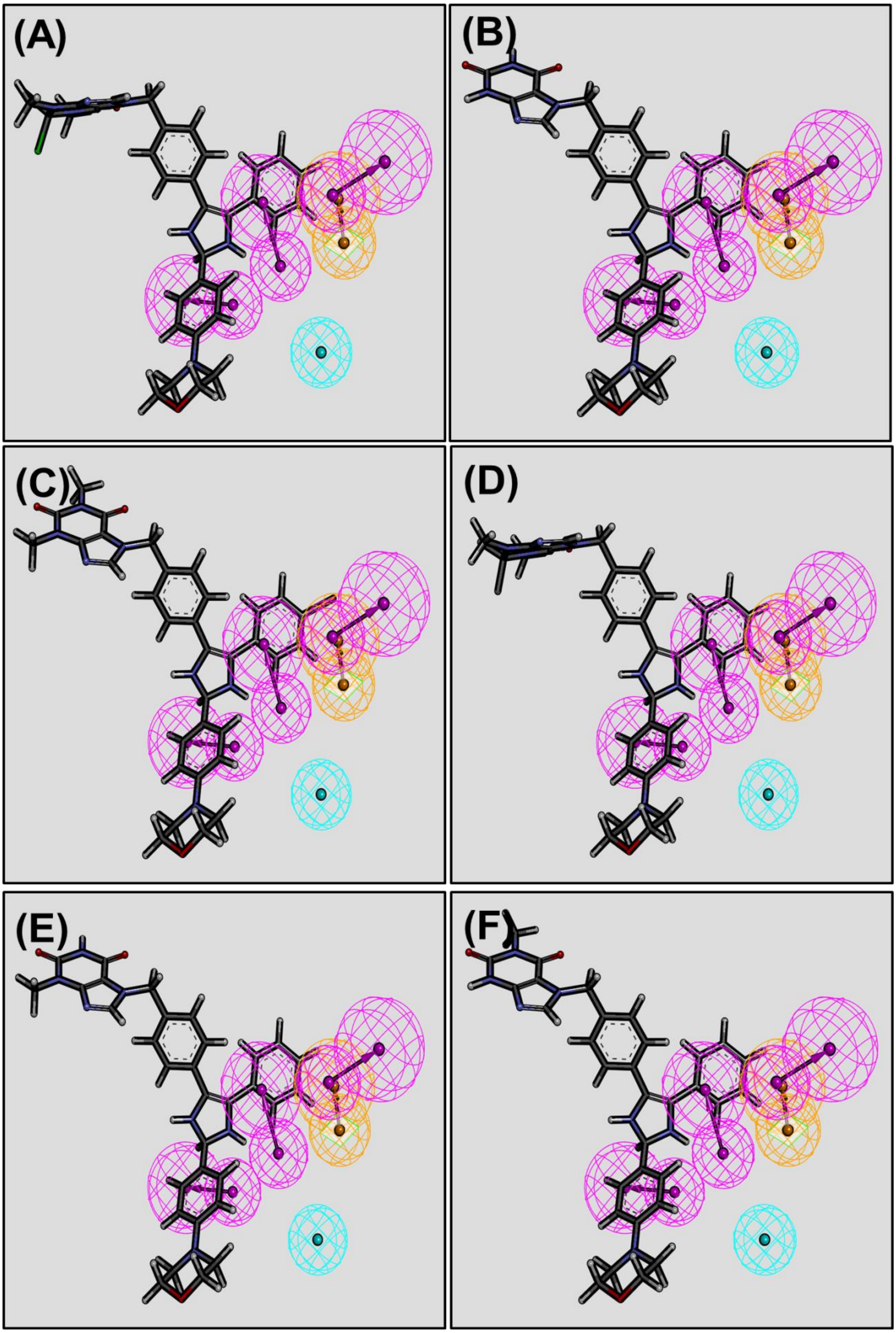

Fig. S5

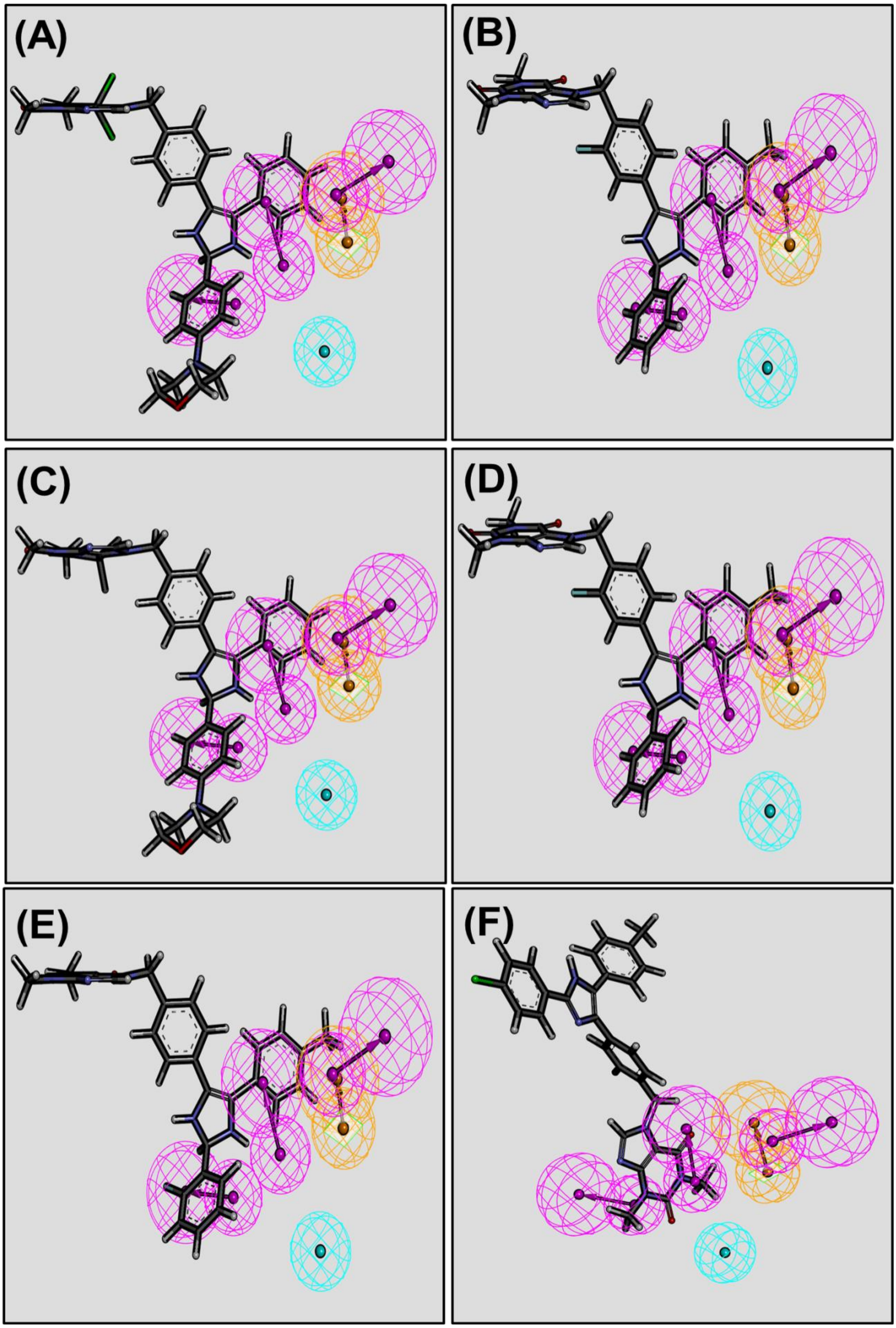

Fig. S6

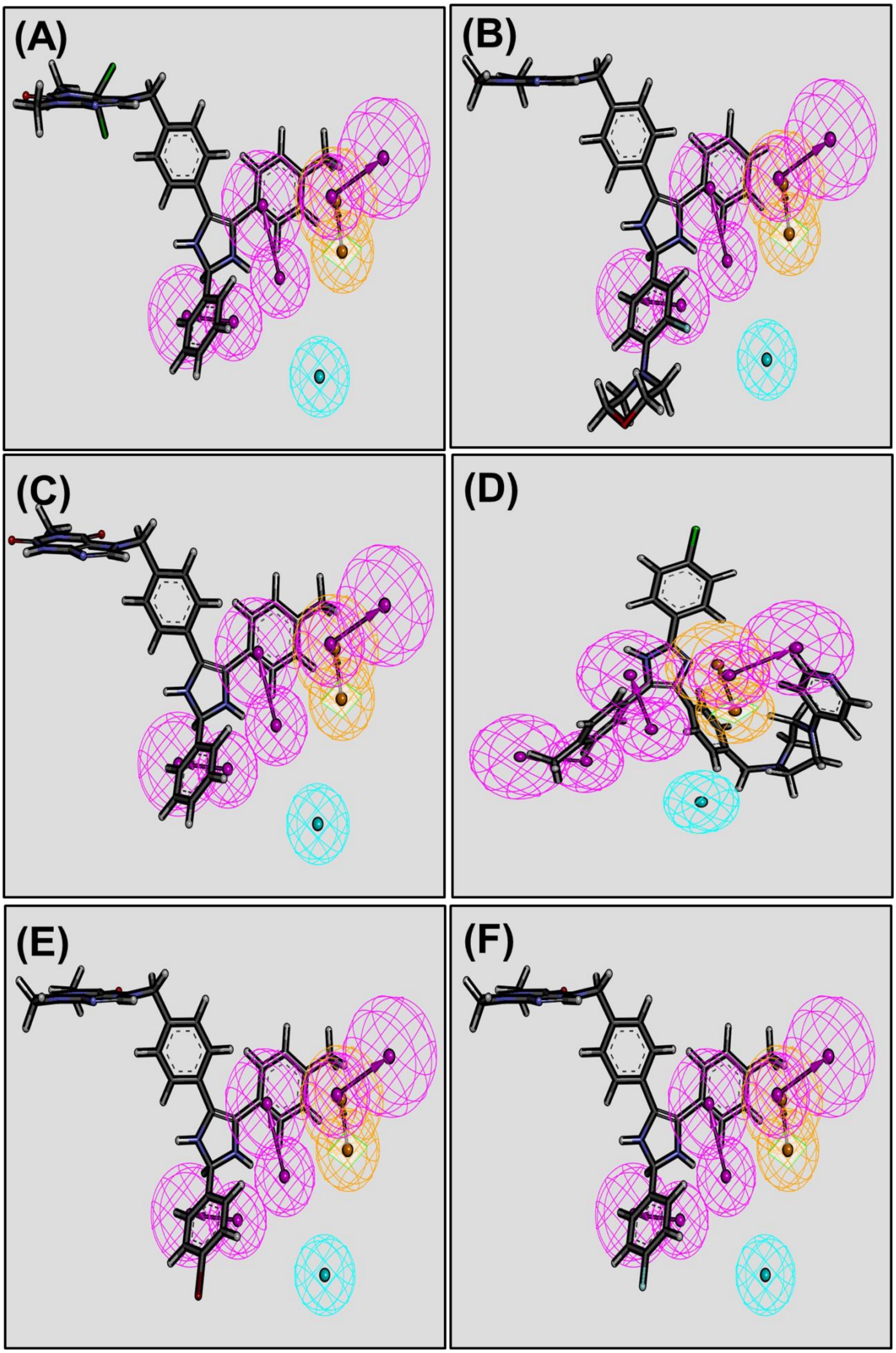

Fig. S7

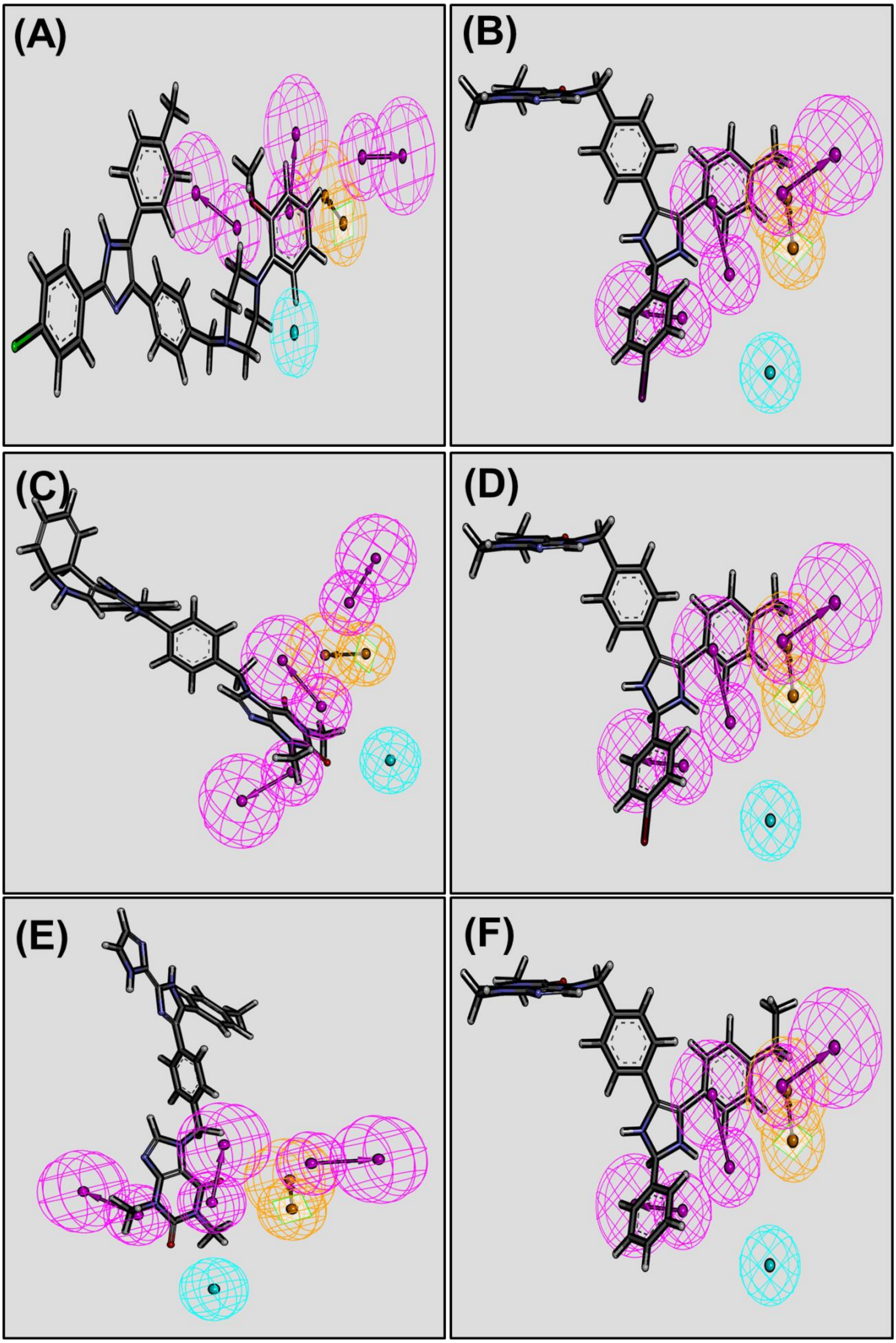

Fig. S8

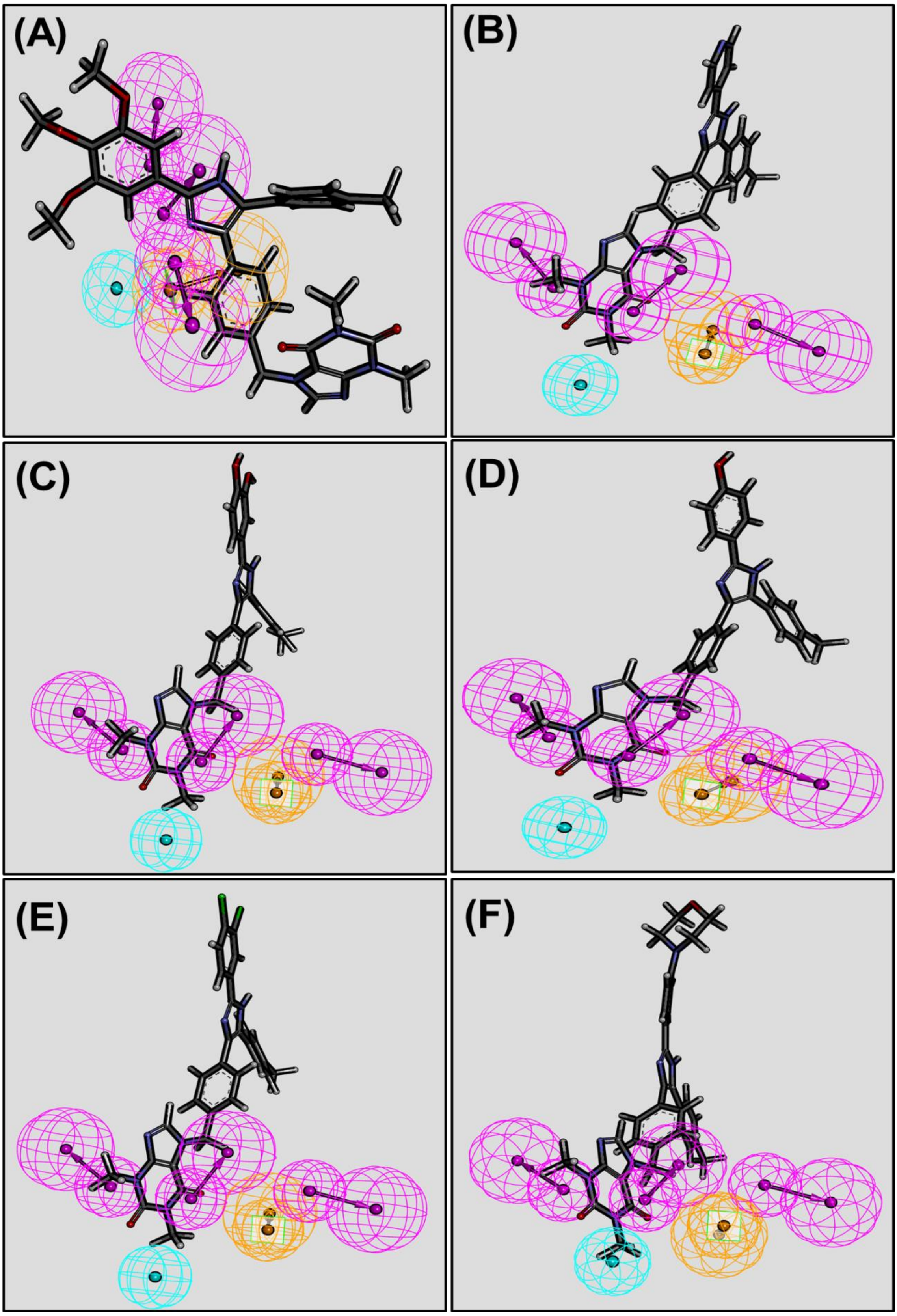

Fig. S9

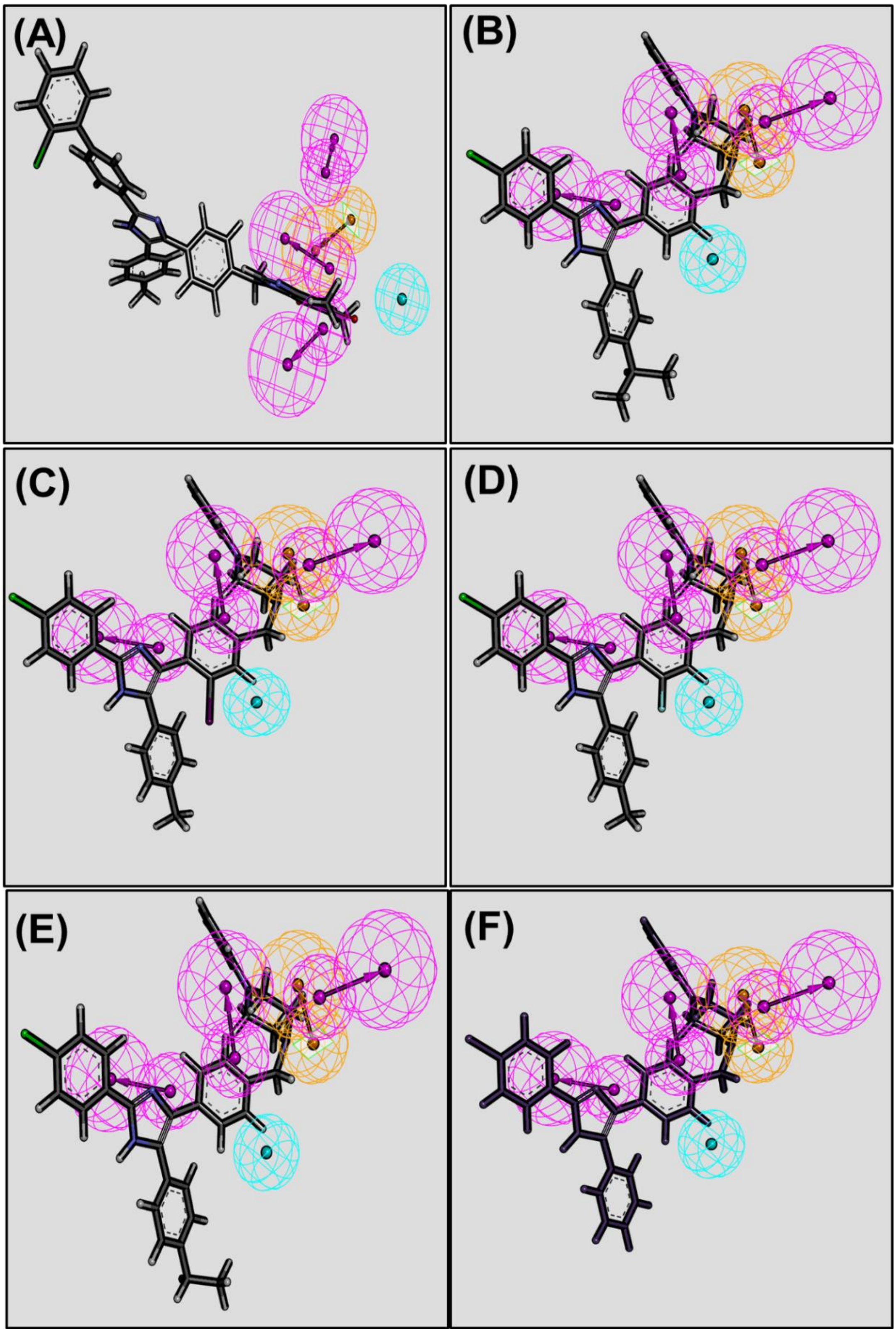

Fig. S10

Characterisation Data

(A)

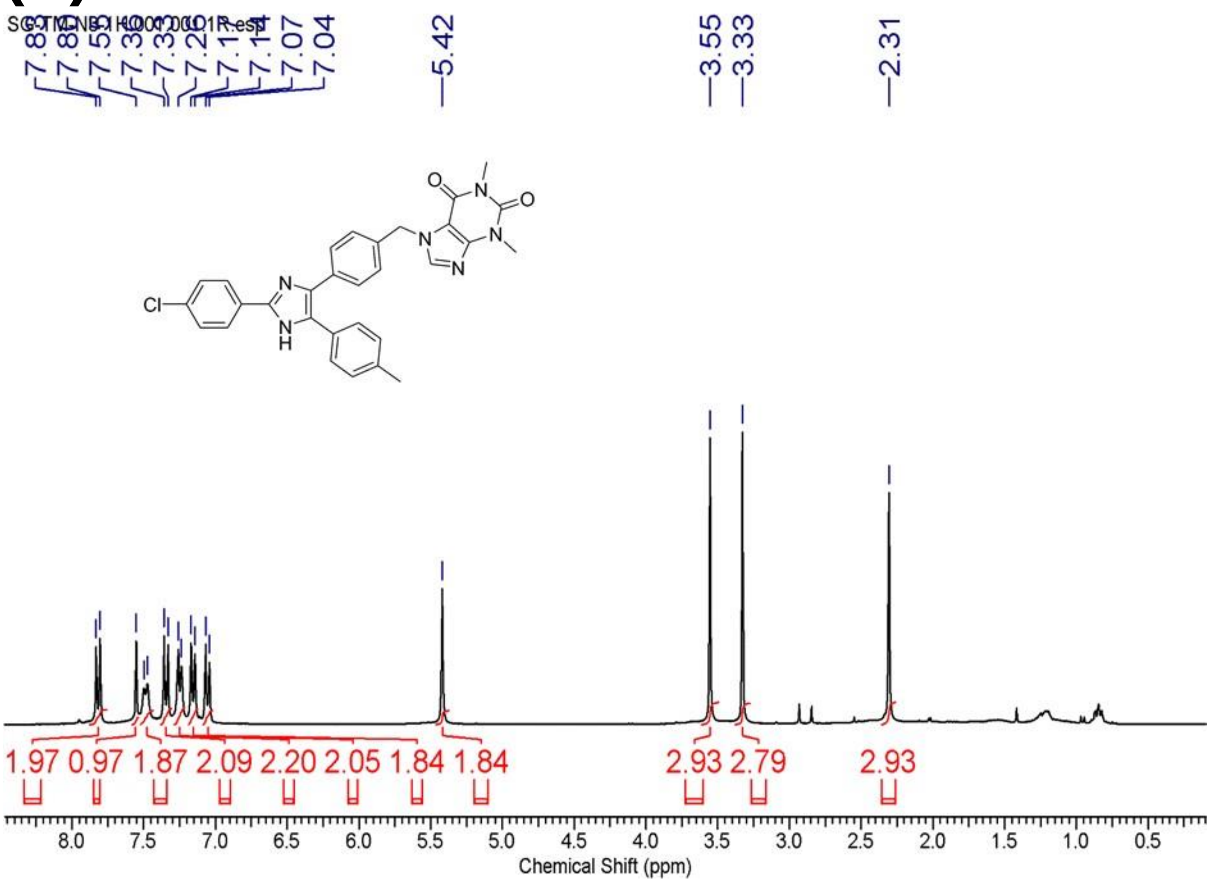

(B)

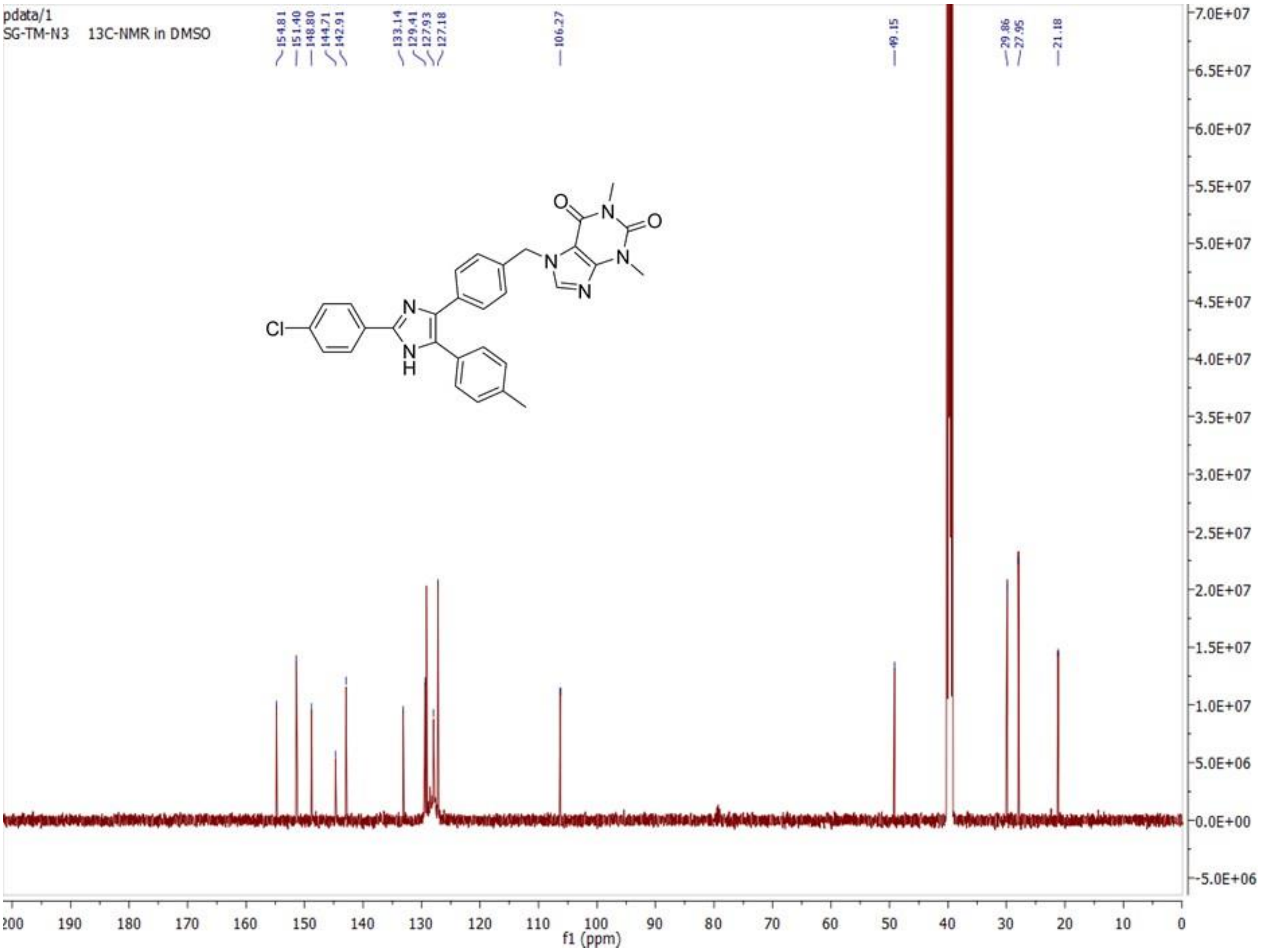

pdata/1
SG-TM-N3 13C-NMR in DMSO
154.81
151.40
148.80
144.71
142.91
133.14
129.41
127.93
127.18
106.27
49.15
29.86
27.95
21.18
Cl
N
NH
N
O
O
N
N
N
f1 (ppm)

(C)

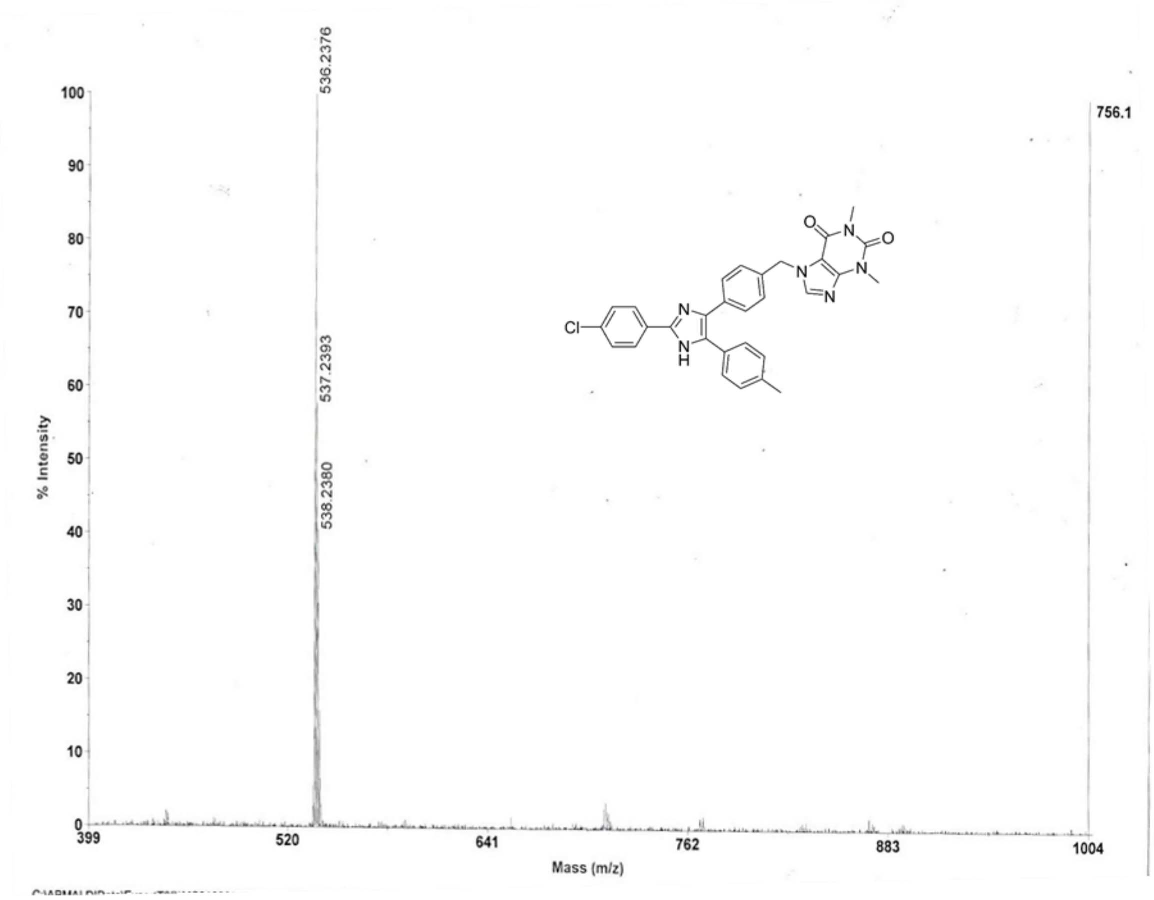

536.2376
756.1
537.2393
538.2380
% Intensity
Mass (m/z)
100
90
80
70
60
50
40
30
20
10
0
399
520
641
762
883
1004
Cl
N
NH
O

(D)

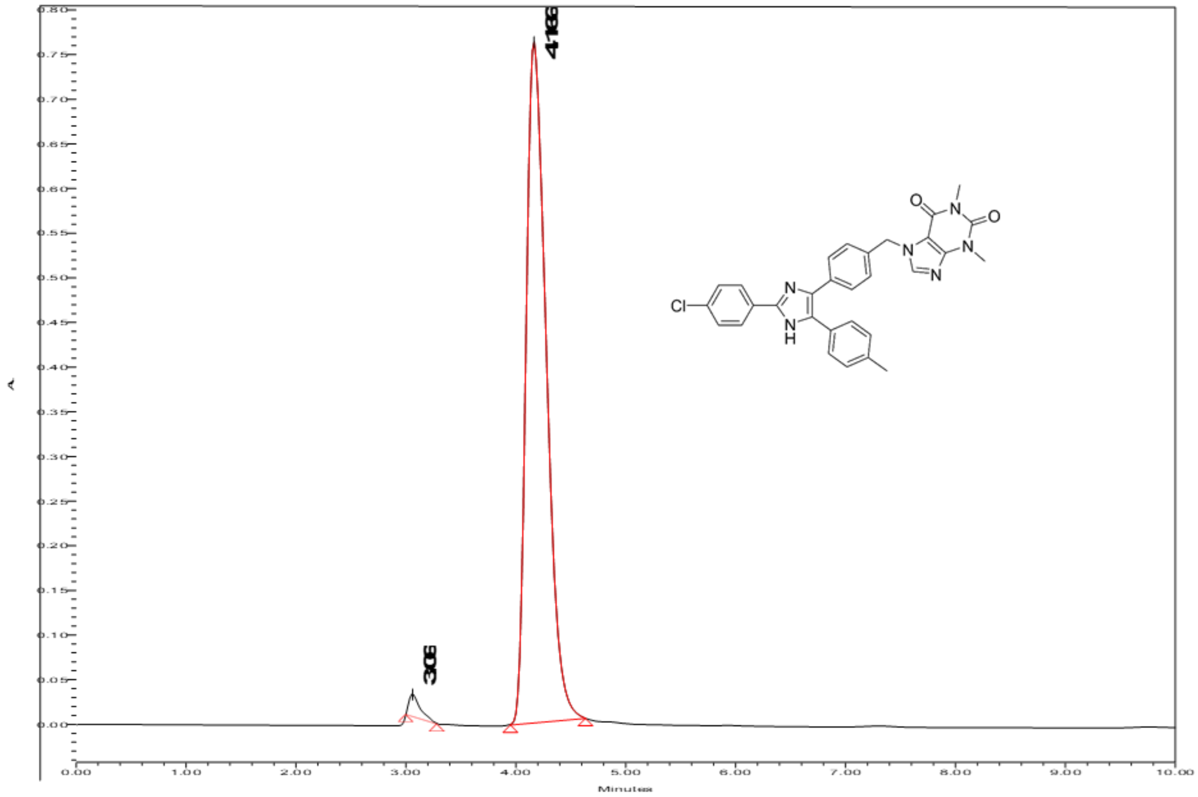

Peak#	Retention Time (min)	Area (µV*sec)	% Area
1	3.061	22791	3.21
2	4.166	687235	96.80

Fig S11. **(A)** ^{1}H NMR (300 MHz, DMSO-d6) spectra of SG-109C, **(B)** ^{13}C NMR (75 MHz, DMSO-d6) of SG-109C, **(C)** HRMS (EI+) Spectra of SG-109C.**(D)** HPLC chromatogram of SG-109C.

(A)

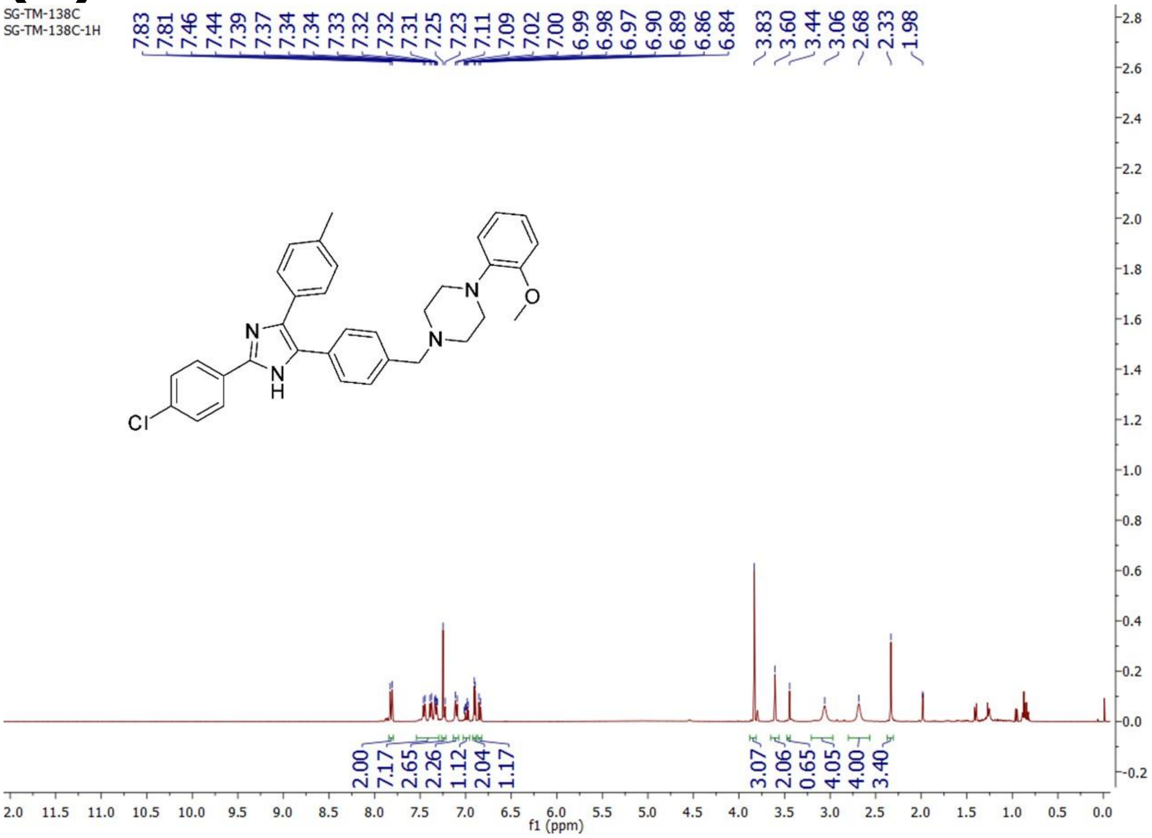

(B)

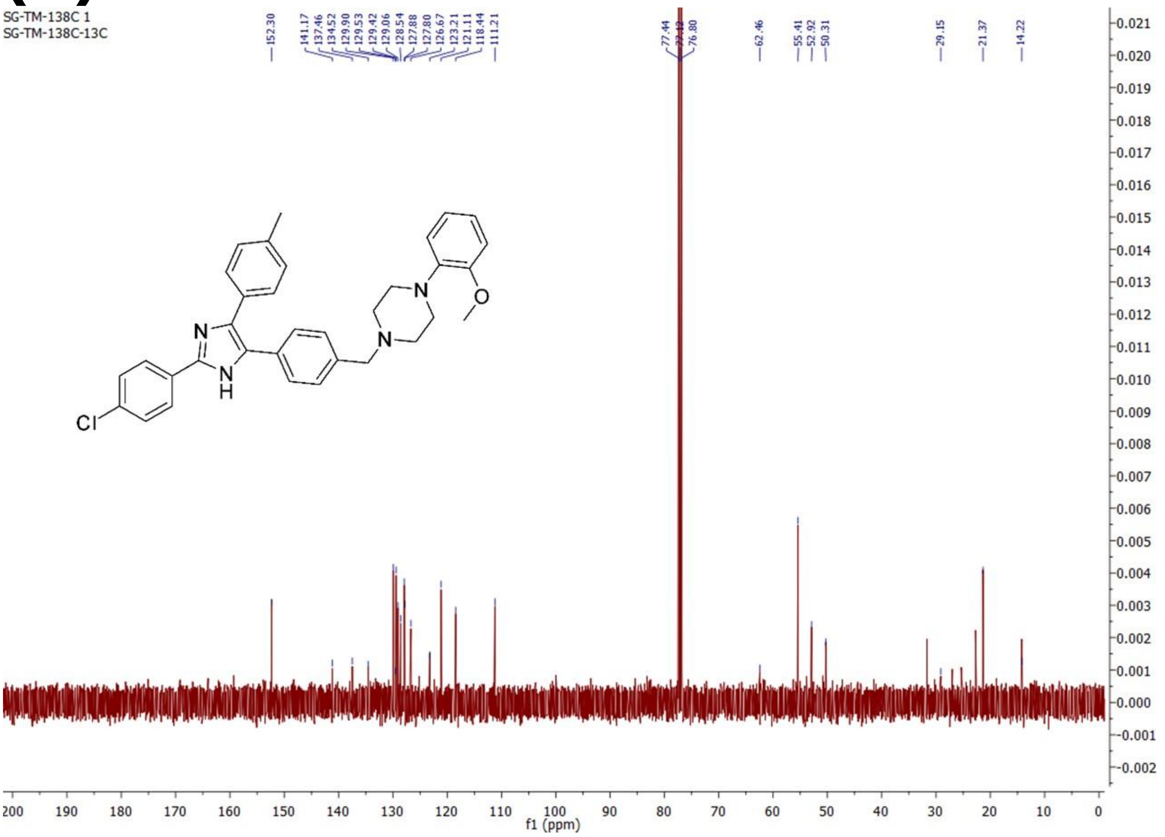
SG-TM-138C 1
SG-TM-138C-13C
152.30
141.17
137.46
134.52
129.90
129.53
129.42
129.06
128.54
127.88
127.80
126.67
123.21
121.11
118.44
111.21
77.44
77.12
76.80
62.46
55.41
52.92
50.31
29.15
21.37
14.22
N
N
H
Cl
N
N
O
f1 (ppm)

(C)

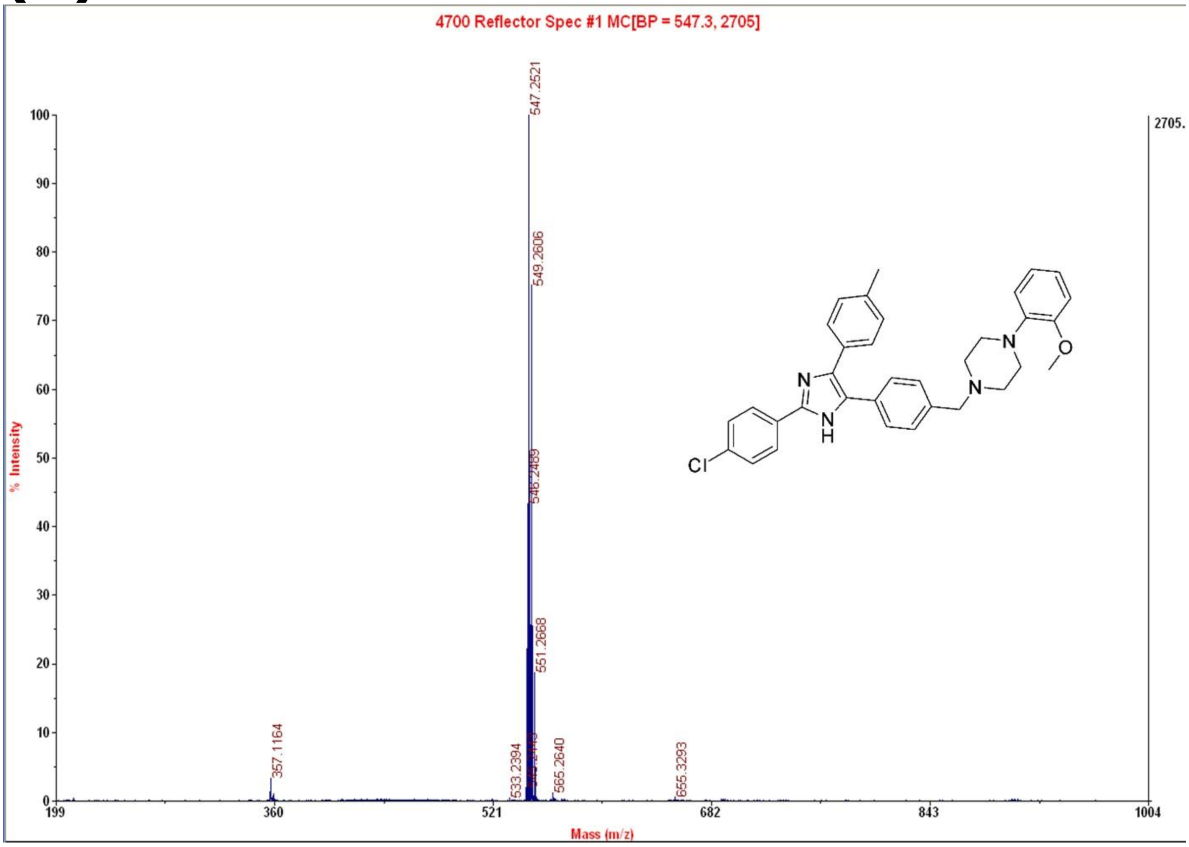

4700 Reflector Spec #1 MC[BP = 547.3, 2705]
547.2521
549.2606
548.2489
551.2668
357.1164
533.2394
565.2640
655.3293
2705.
% Intensity
Mass (m/z)
199
360
521
682
843
1004
100
90
80
70
60
50
40
30
20
10
0
N
H
Cl
O

(D)

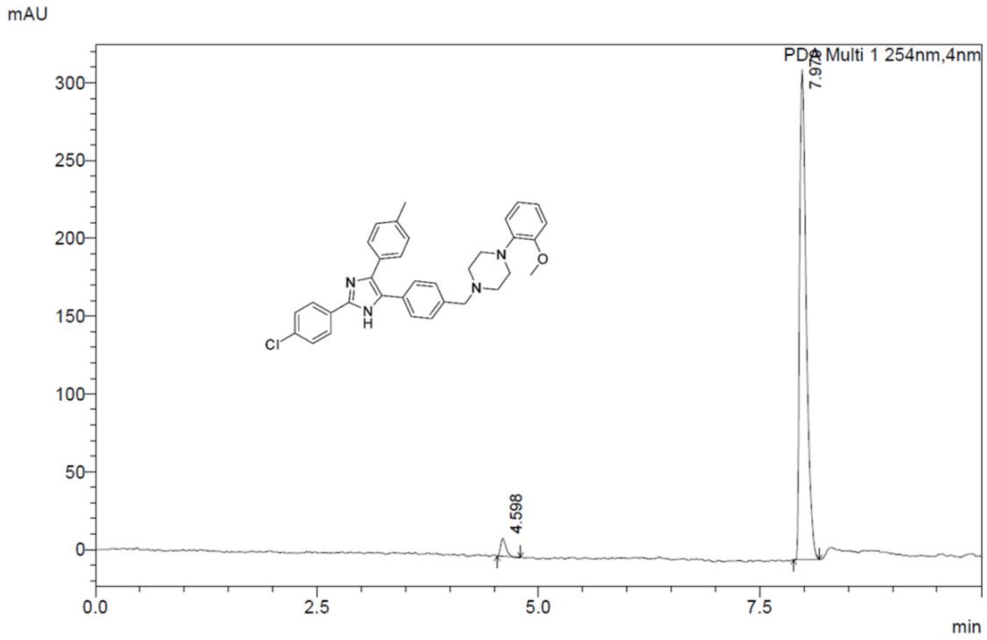

Peak#	Ret. Time	Area	Height	Area%
1	4.598	44827	11402	2.638
2	7.979	1654187	315153	97.362
Total		1699014	326555	100.000

Fig S12. **(A)** ^{1}H NMR (400 MHz, CHLOROFORM-*d*) spectra of SG-138C, **(B)** ^{13}C NMR (100MHz, CHLOROFORM-*d*) of SG-138C, **(C)** HRMS (EI-) Spectra of SG-138C. **(D)** HPLC chromatogram of SG-138C.

(A)

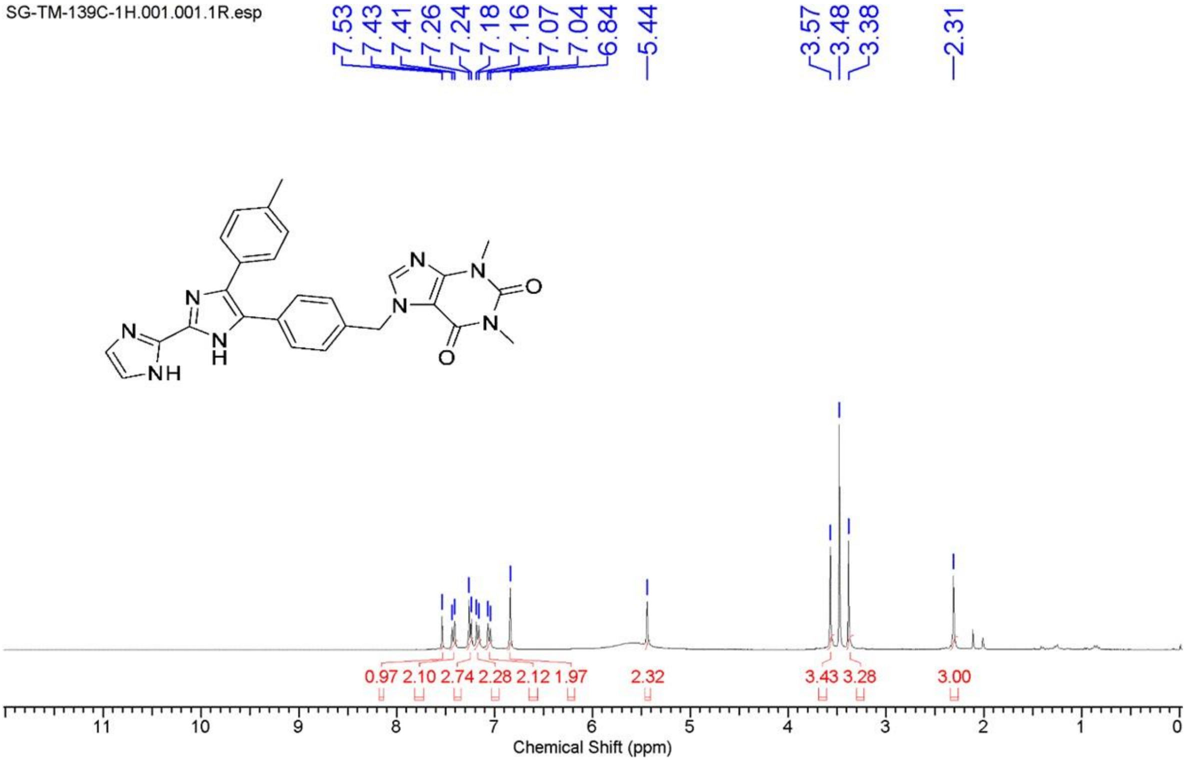
SG-TM-139C-1H.001.001.1R.esp
7.53 7.43 7.41 7.26 7.24 7.18 7.16 7.07 7.04 6.84
5.44
3.57 3.48 3.38
2.31
N
N
N
O
N
O
N
N
NH
N
H
0.97 2.10 2.74 2.28 2.12 1.97
2.32
3.43 3.28
3.00
11 10 9 8 7 6 5 4 3 2 1 0
Chemical Shift (ppm)

(B)

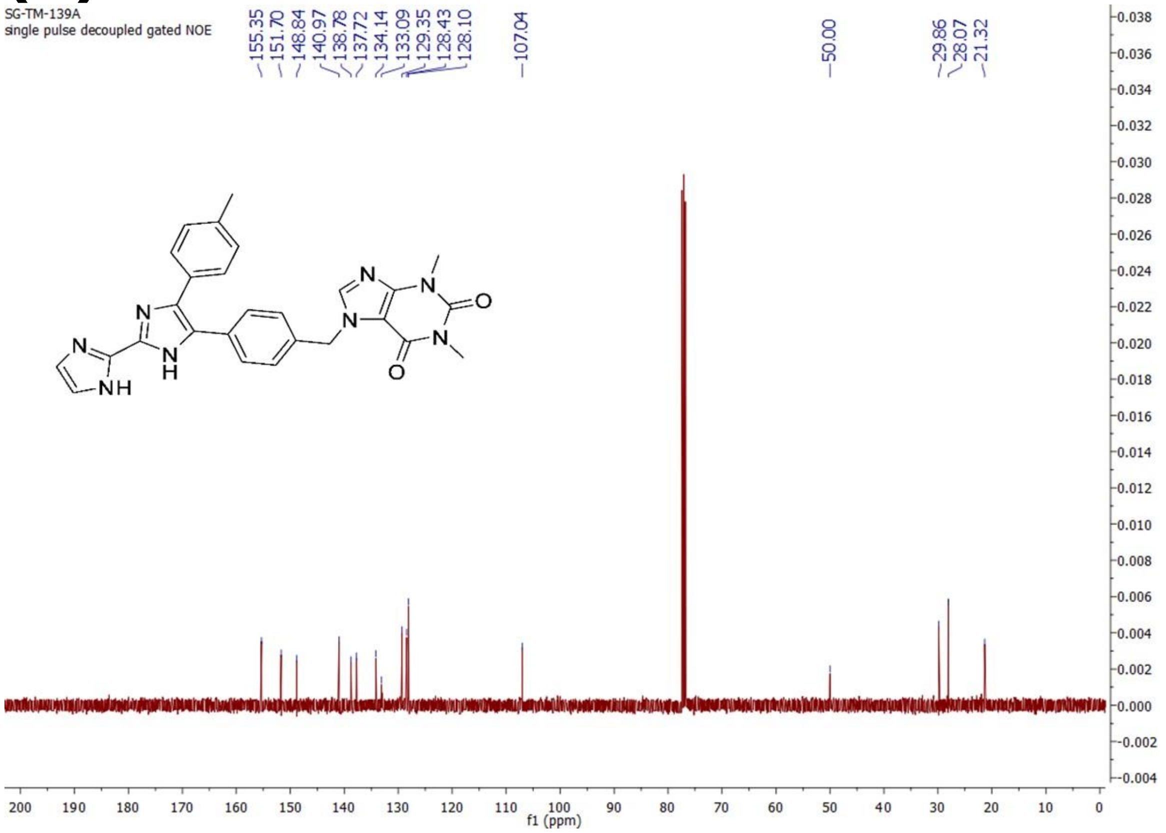
SG-TM-139A
single pulse decoupled gated NOE
155.35
151.70
148.84
140.97
138.78
137.72
134.14
133.09
129.35
128.43
128.10
107.04
50.00
29.86
28.07
21.32
f1 (ppm)

(C)

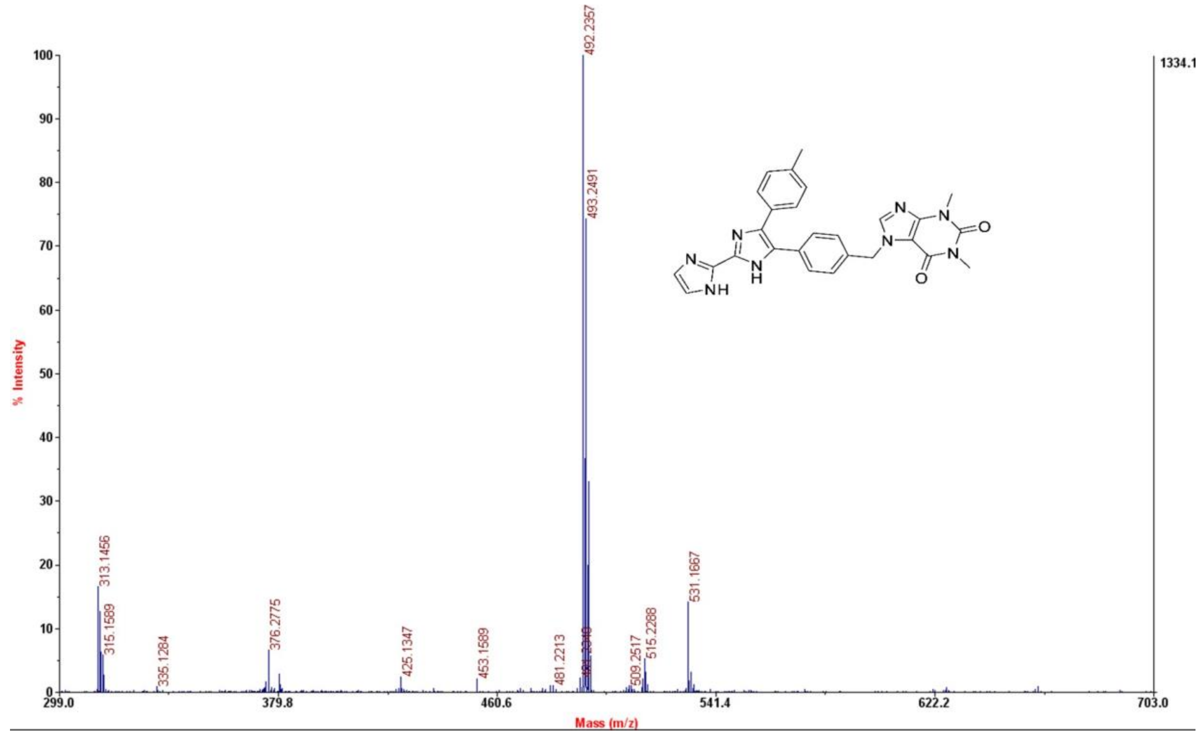

492.2357
493.2491
313.1456
315.1589
335.1284
376.2775
425.1347
453.1589
481.2213
509.2517
515.2288
531.1667
1334.1
% Intensity
Mass (m/z)
299.0
379.8
460.6
541.4
622.2
703.0

(D)

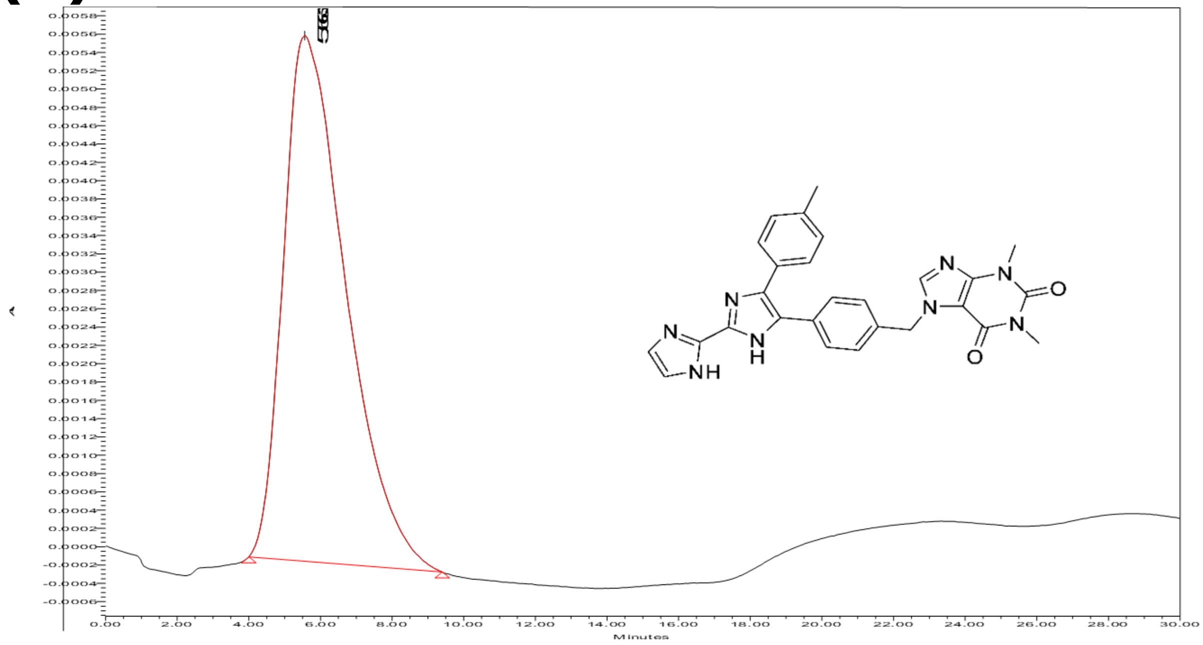

Peak#	Retention Time (min)	Area (µV*sec)	% Area
1	5.563	699227	100.00

Fig S13. (A) ^{1}H NMR (300 MHz, CHLOROFORM-*d*) spectra of SG-139C, **(B)** ^{13}C NMR (100MHz, CHLOROFORM-*d*) of SG-139C, **(C)** HRMS (EI+) Spectra of SG-139C. **(D)** HPLC chromatogram of SG-139C.

(A)

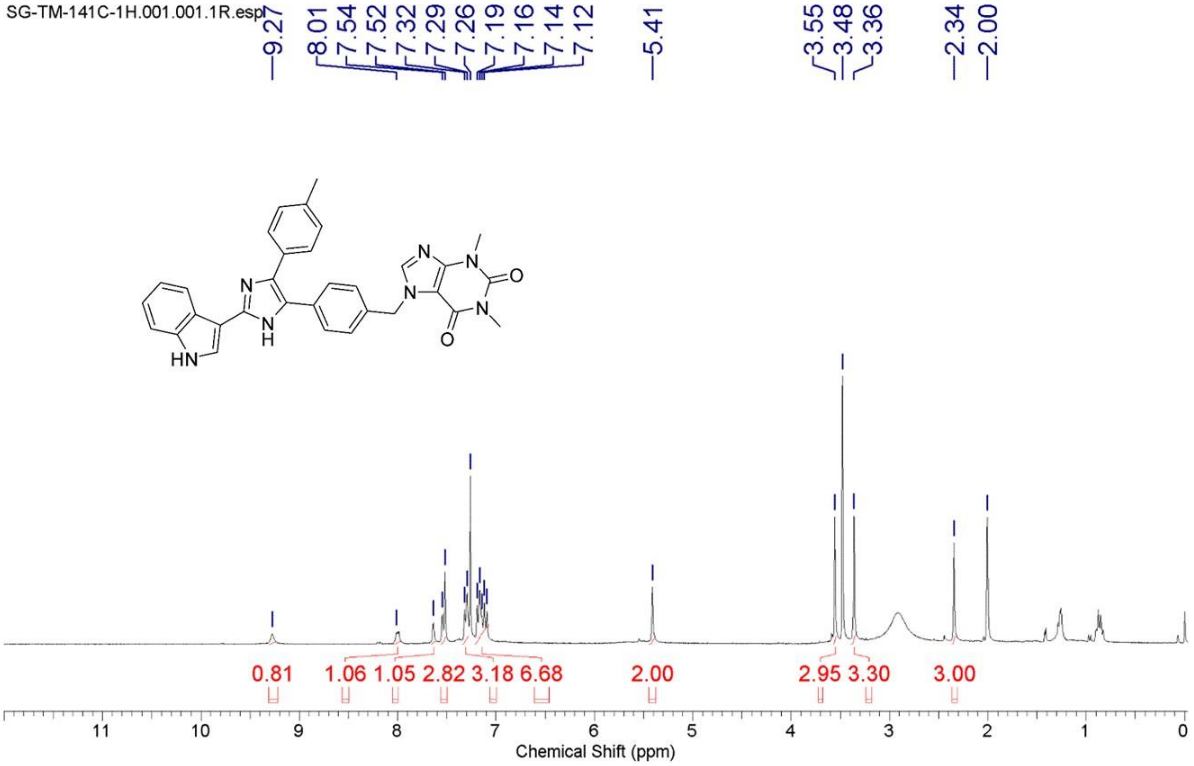
SG-TM-141C-1H.001.001.1R.esp
9.27
8.01
7.54
7.52
7.32
7.29
7.26
7.19
7.16
7.14
7.12
5.41
3.55
3.48
3.36
2.34
2.00
HN
N
H
N
N
N
N
O
O
0.81
1.06
1.05
2.82
3.18
6.68
2.00
2.95
3.30
3.00
11
10
9
8
7
6
5
4
3
2
1
0
Chemical Shift (ppm)

(B)

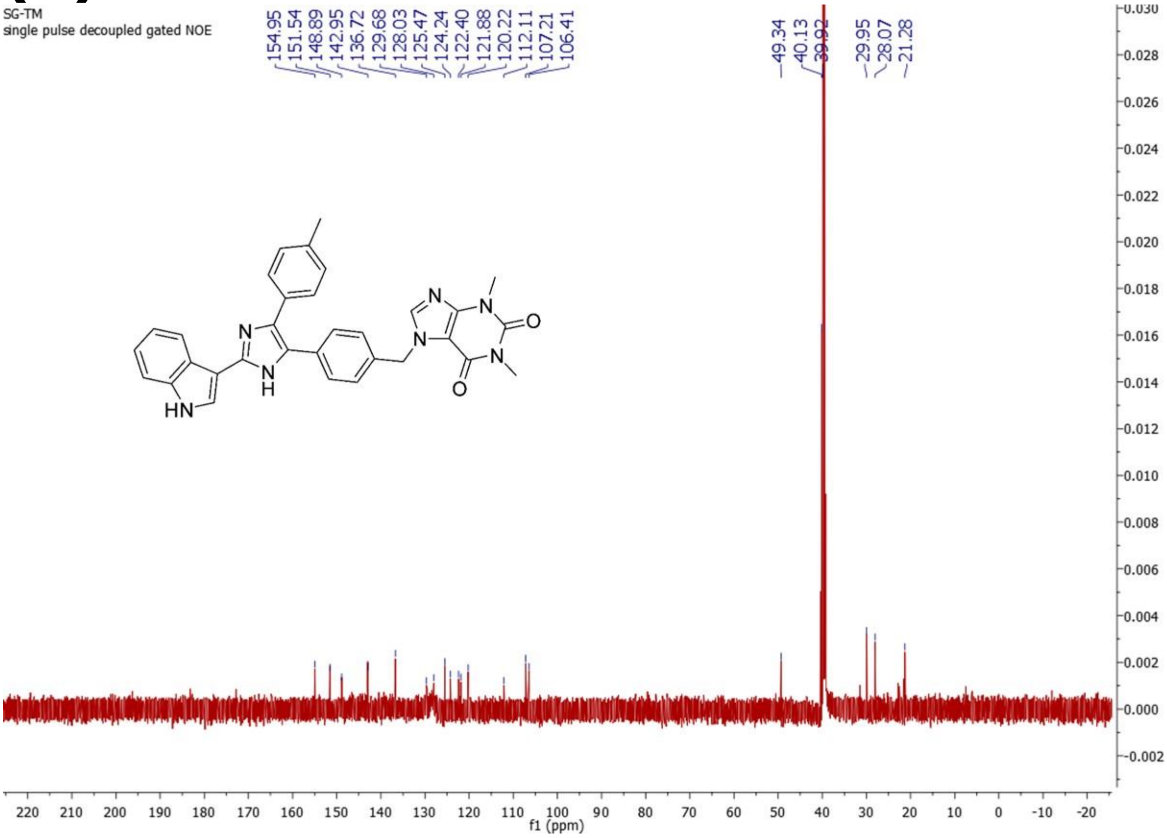
SG-TM
single pulse decoupled gated NOE
154.95
151.54
148.89
142.95
136.72
129.68
128.03
125.47
124.24
122.40
121.88
120.22
112.11
107.21
106.41
49.34
40.13
39.92
29.95
28.07
21.28
f1 (ppm)

(C)

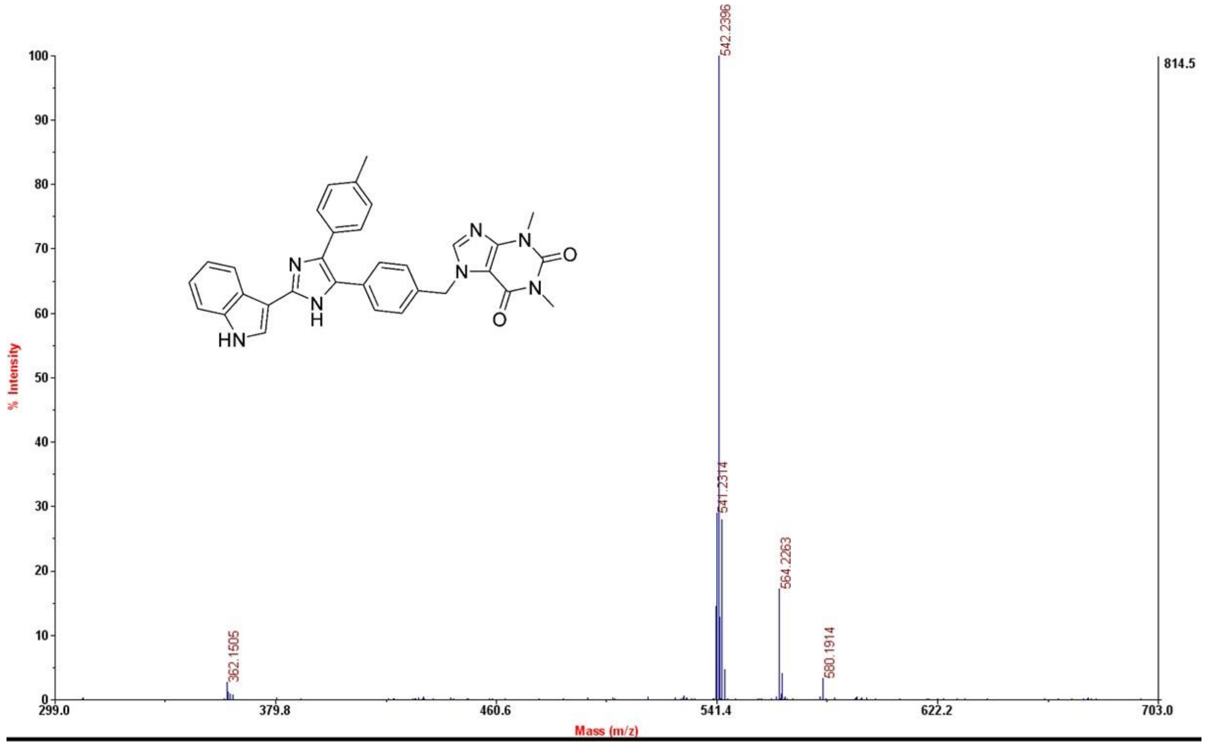
542.2396
541.2314
564.2263
580.1914
362.1505
814.5
% Intensity
Mass (m/z)
100
90
80
70
60
50
40
30
20
10
0
299.0
379.8
460.6
541.4
622.2
703.0
N
HN
N
H
N
N
N
N
O
O

(D)

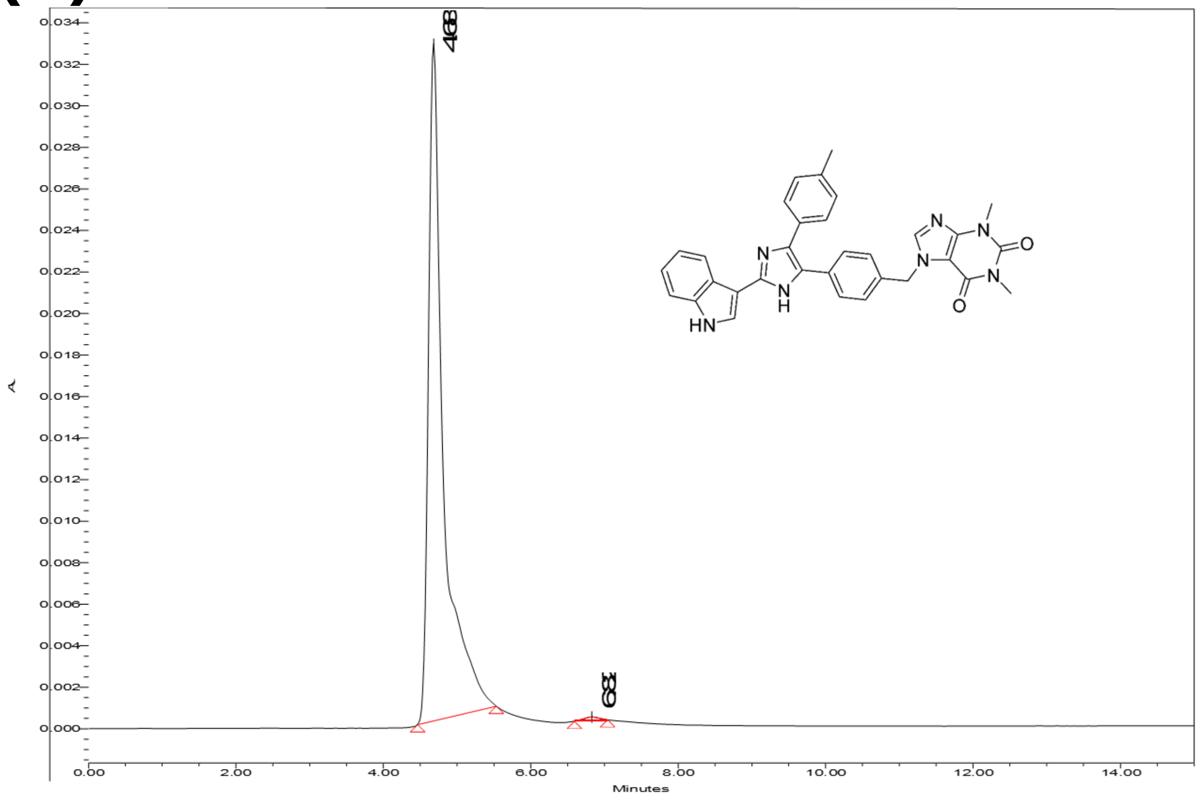

Peak#	Retention Time (min)	Area (µV*sec)	% Area
1	4.682	265788	99.02
2	6.829	2604	0.98

Fig S14. (A) ^{1}H NMR (300 MHz, DMSO-d6) spectra of SG-141C, **(B)** ^{13}C NMR (100MHz, DMSO-d6) of SG-141C, **(C)** HRMS (EI+) Spectra of SG-141C. **(D)** HPLC chromatogram of SG-141C.

(A)

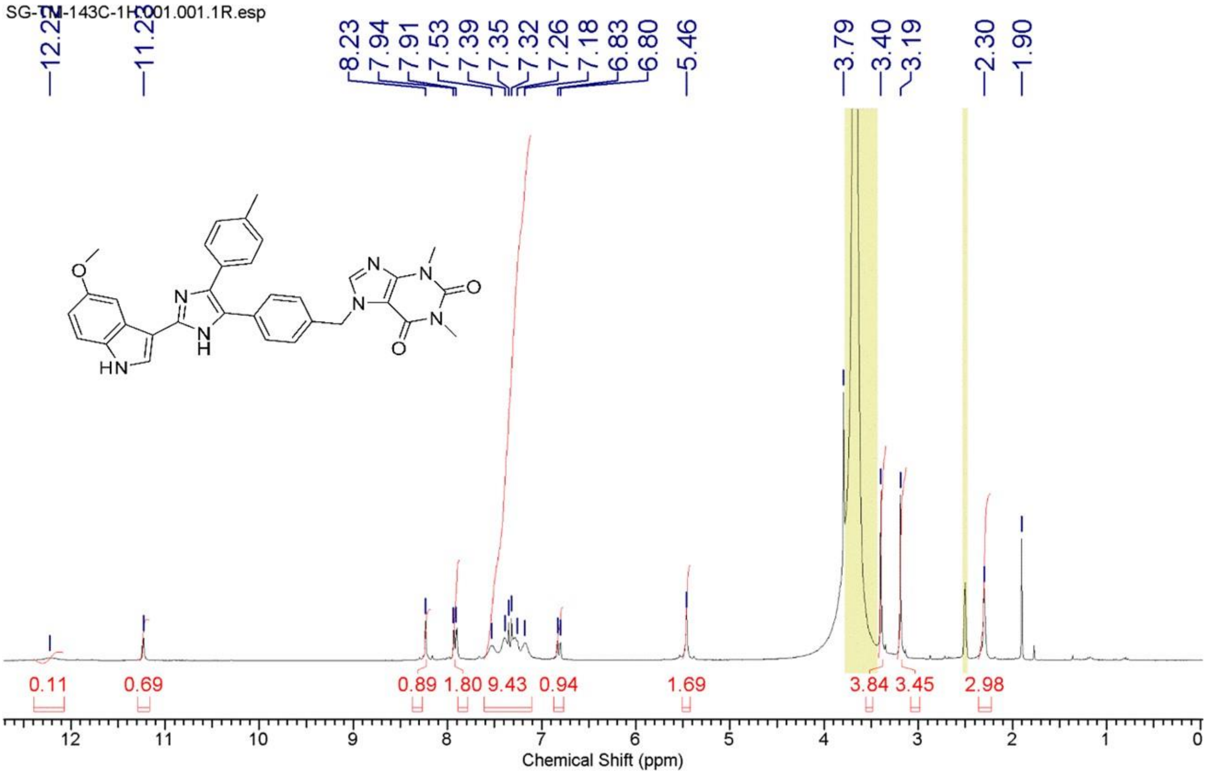

SG-TM-143C-1H.001.001.1R.esp
12.22
11.22
8.23
7.94
7.91
7.53
7.39
7.35
7.32
7.26
7.18
6.83
6.80
5.46
3.79
3.40
3.19
2.30
1.90
HN
N
H
N
O
0.11
0.69
0.89
1.80
9.43
0.94
1.69
3.84
3.45
2.98
Chemical Shift (ppm)

(B)

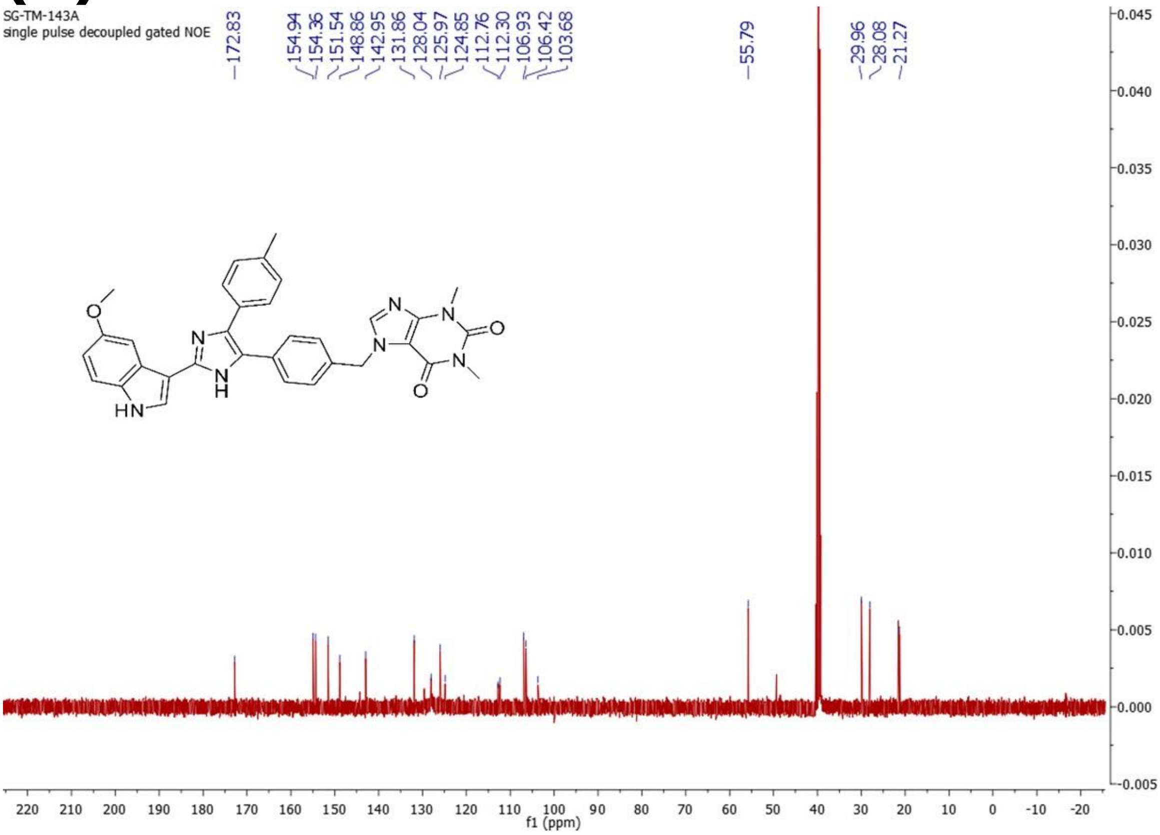

SG-TM-143A
single pulse decoupled gated NOE
172.83
154.94
154.36
151.54
148.86
142.95
131.86
128.04
125.97
124.85
112.76
112.30
106.93
106.42
103.68
55.79
29.96
28.08
21.27
f1 (ppm)

(C)

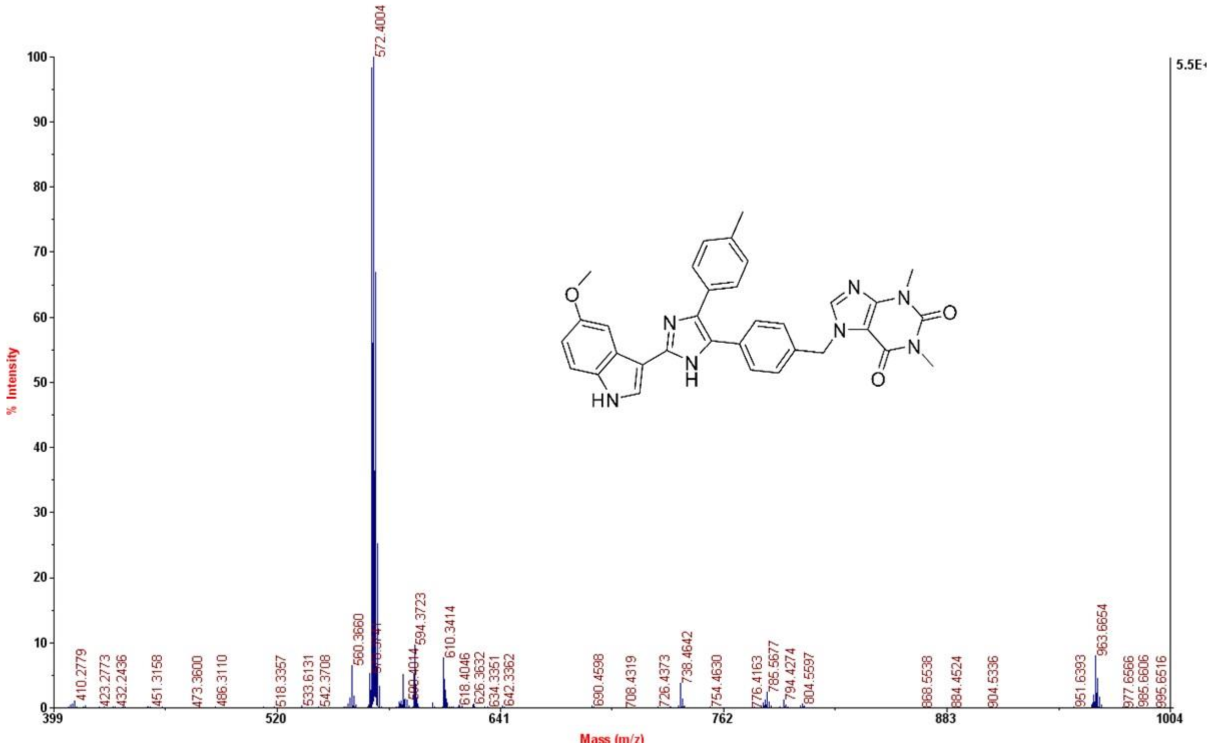

(D)

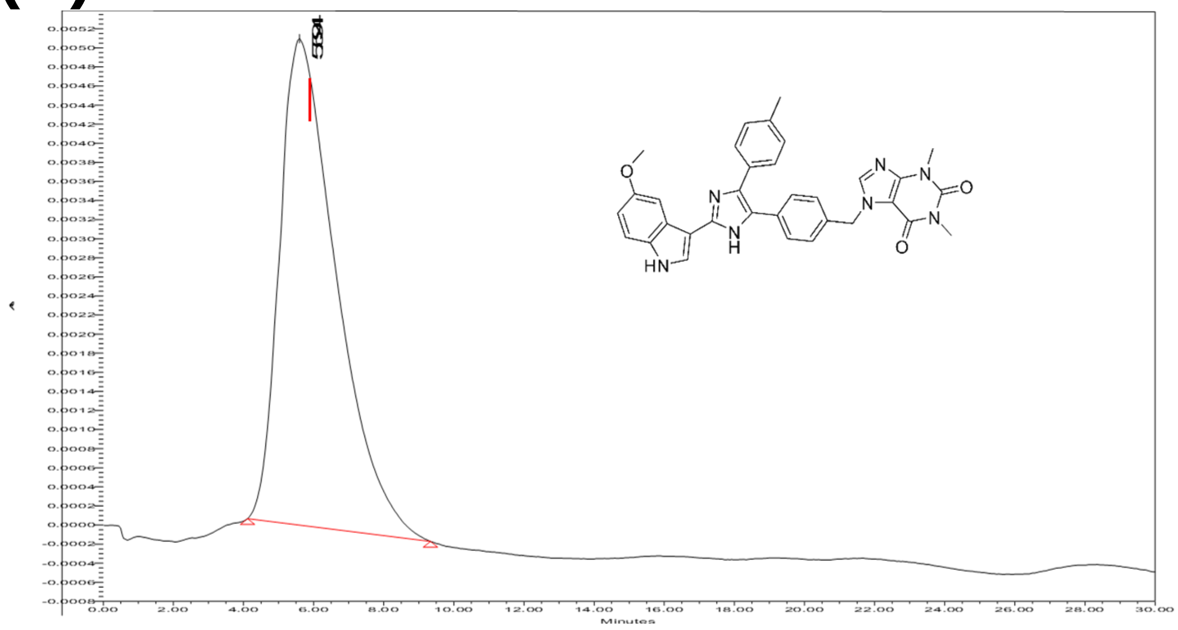

Peak#	Retention Time (min)	Area (µV*sec)	% Area
1	5.594	576497	100.00

Fig S15. **(A)** ^{1}H NMR (300 MHz, DMSO-d_6) spectra of SG-143C, **(B)** ^{13}C NMR (100MHz, DMSO-d_6) of SG-143C, **(C)** HRMS (EI+) Spectra of SG-143C. **(D)** HPLC chromatogram of SG-143C.

(A)

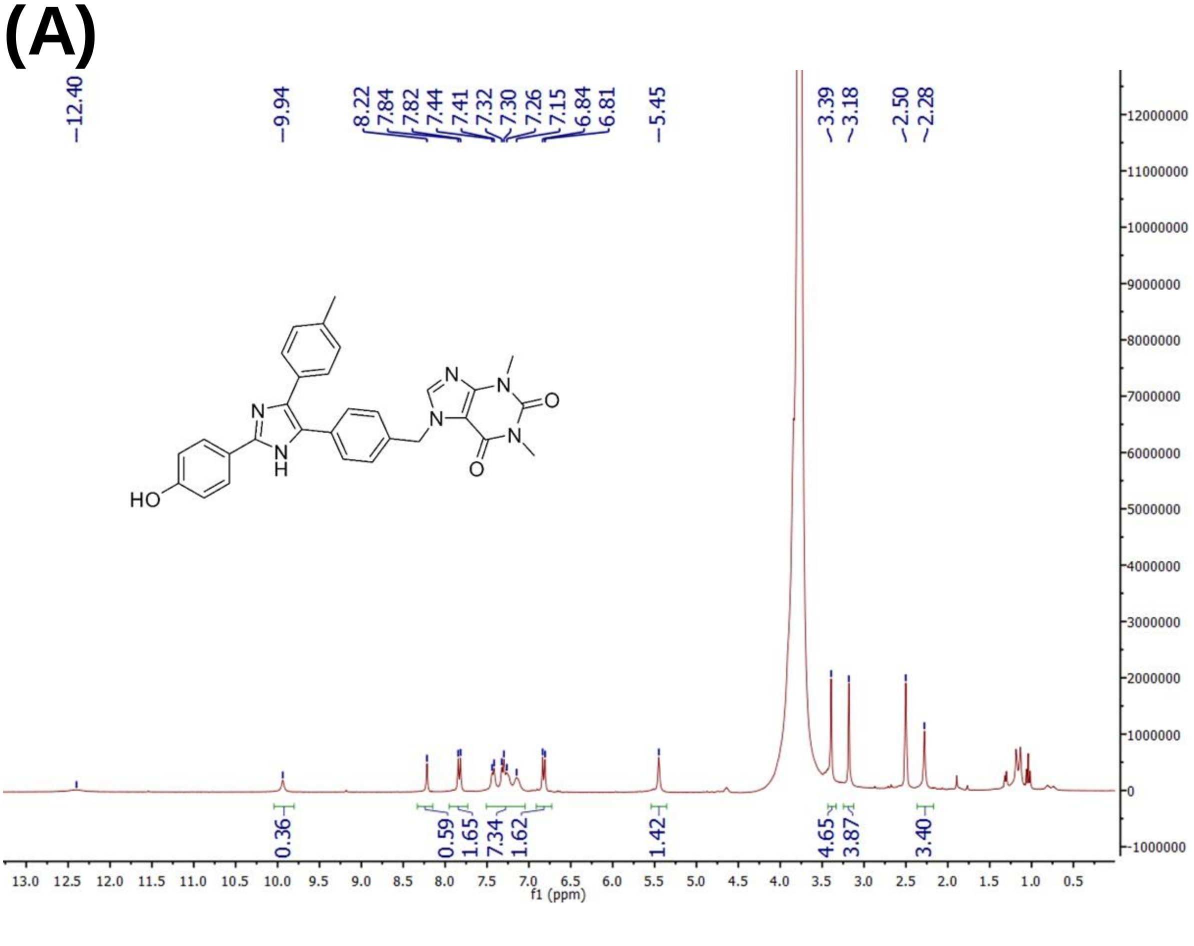

(B)

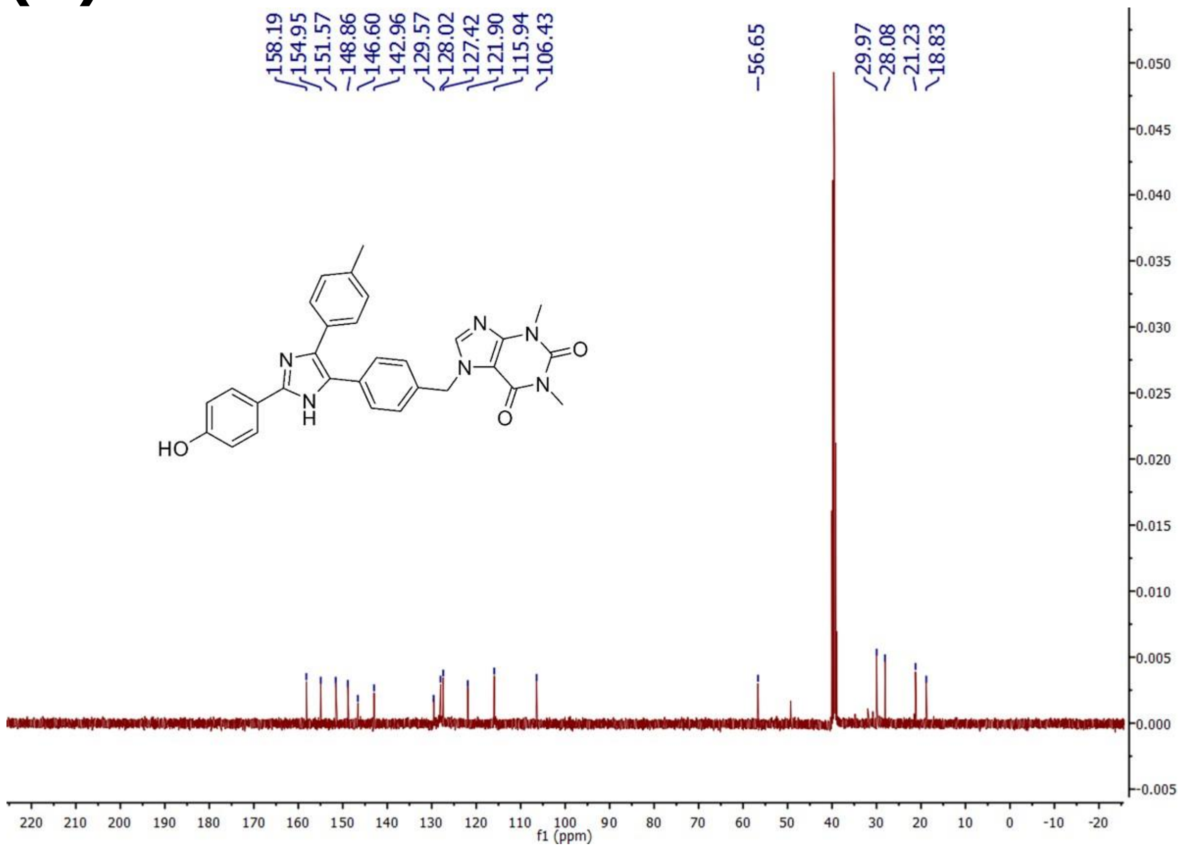

158.19
154.95
151.57
148.86
146.60
142.96
129.57
128.02
127.42
121.90
115.94
106.43
56.65
29.97
28.08
21.23
18.83
HO
N
H
N
N
N
N
O
O
f1 (ppm)

(C)

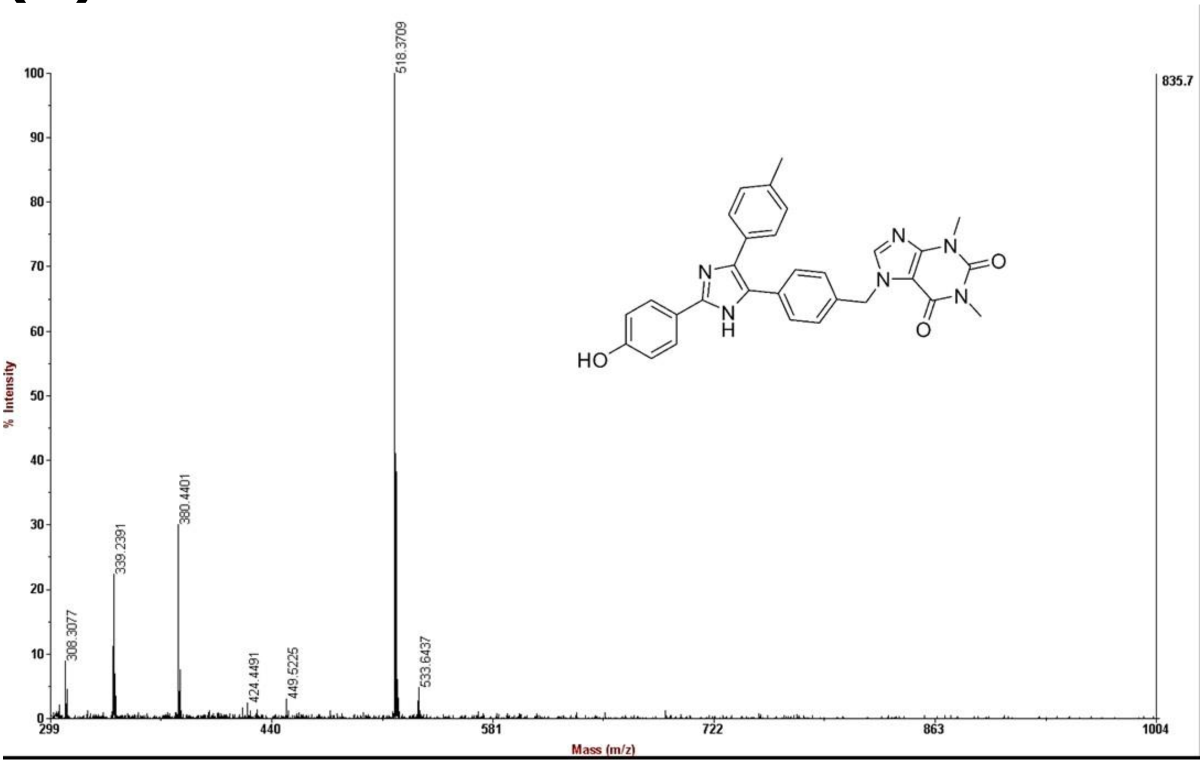
518.3709
835.7
100
90
80
70
60
50
40
30
20
10
0
% Intensity
380.4401
339.2391
308.3077
424.4491
449.5225
533.6437
299
440
581
722
863
1004
Mass (m/z)
HO
N
H
N
N
N
N
N
O
O

(D)

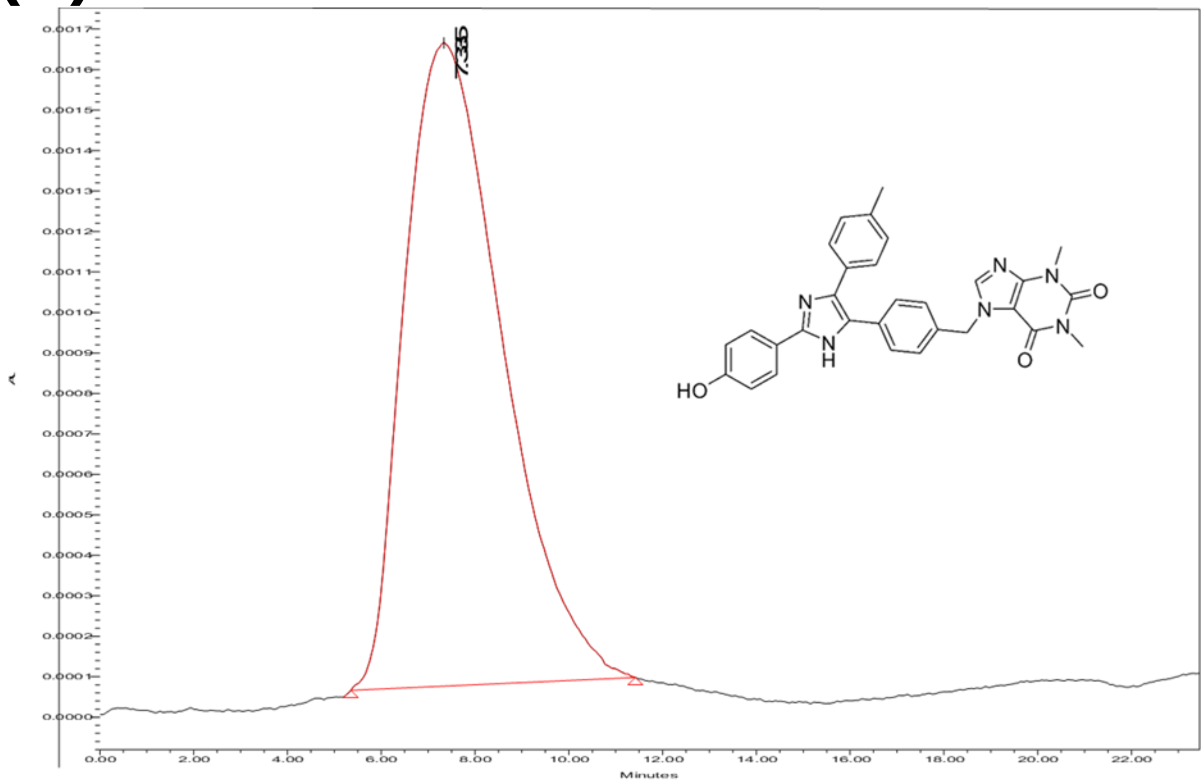

Peak#	Retention Time (min)	Area (µV*sec)	% Area
1	7.335	221886	100

Fig S16. (A) ^{1}H NMR (300 MHz, DMSO-d_6) spectra of SG-145C, **(B)** ^{13}C NMR (100MHz, DMSO-d_6) of SG-145C, **(C)** HRMS (EI+) Spectra of SG-145C. **(D)** HPLC chromatogram of SG-145C.

(A)

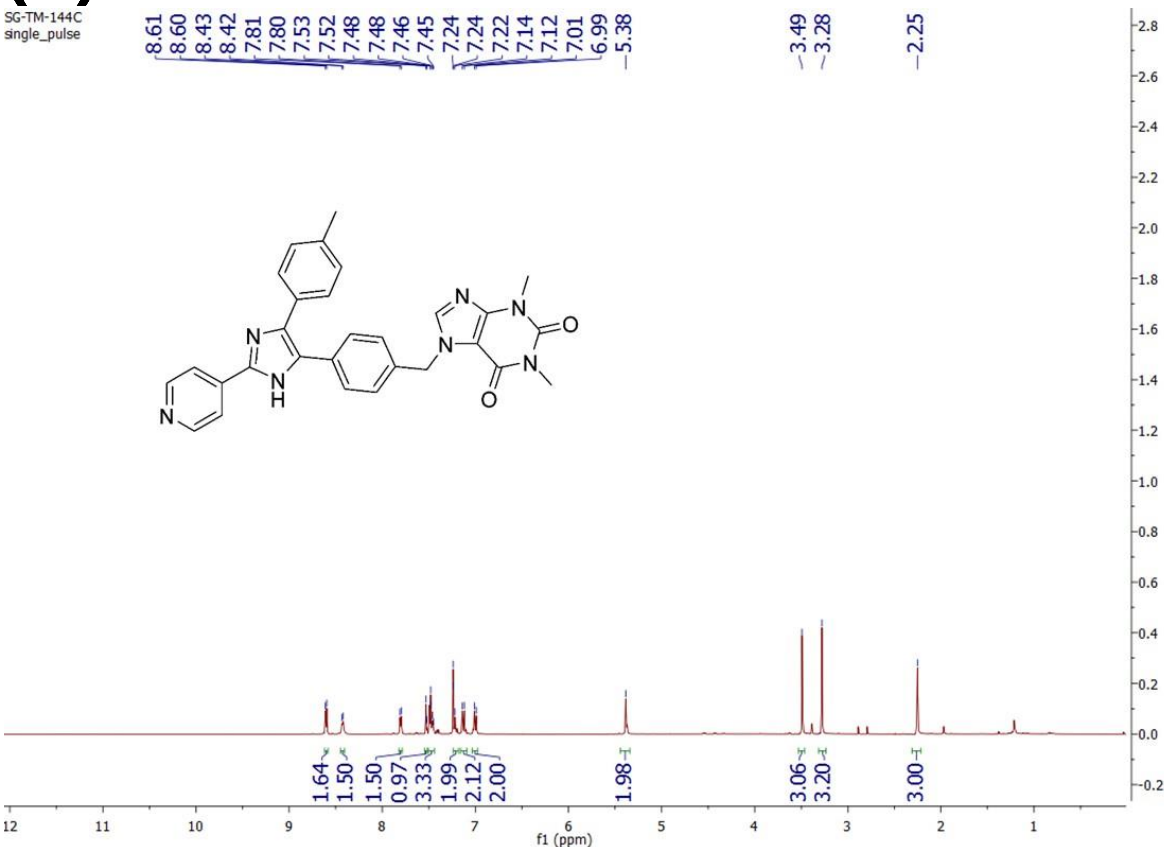
SG-TM-144C
single_pulse
8.61
8.60
8.43
8.42
7.81
7.80
7.53
7.52
7.48
7.48
7.46
7.45
7.24
7.24
7.22
7.14
7.12
7.01
6.99
5.38
3.49
3.28
2.25
1.64
1.50
1.50
0.97
3.33
1.99
2.12
2.00
1.98
3.06
3.20
3.00
f1 (ppm)

(B)

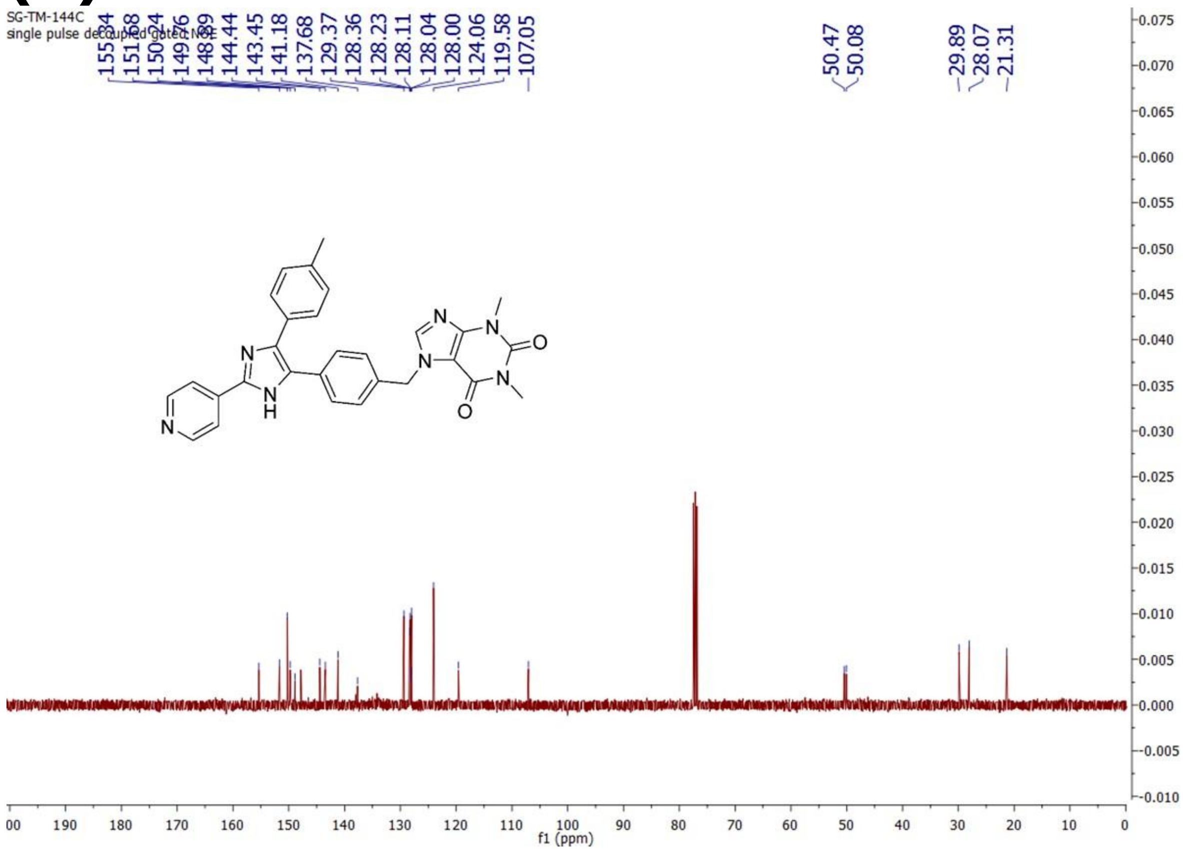
SG-TM-144C
single pulse decoupled gated NOE
155.34
151.68
150.24
149.76
148.89
144.44
143.45
141.18
137.68
129.37
128.36
128.23
128.11
128.04
128.00
124.06
119.58
107.05
50.47
50.08
29.89
28.07
21.31
f1 (ppm)

(C)

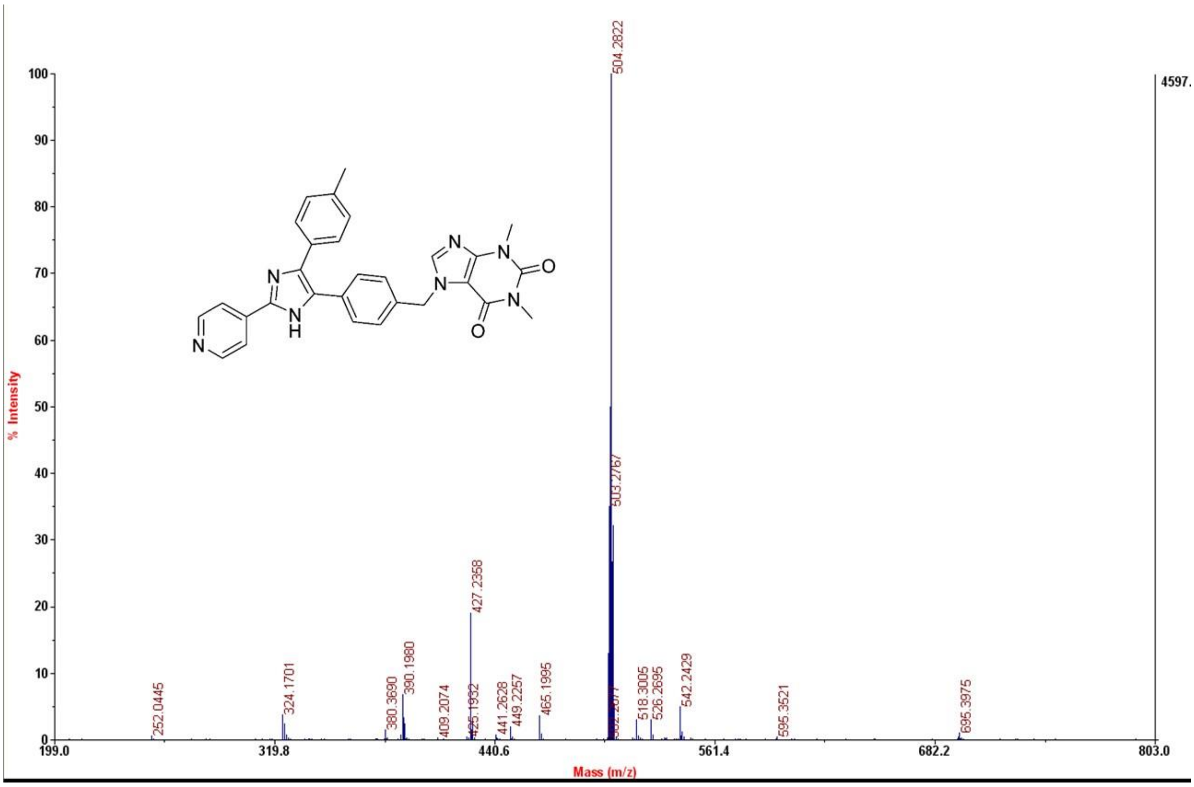

100
90
80
70
60
50
40
30
20
10
0
% Intensity
504.2822
4597.
503.2767
427.2358
390.1980
380.3690
324.1701
252.0445
409.2074
441.2628
449.2257
465.1995
518.3005
526.2695
542.2429
595.3521
695.3975
199.0
319.8
440.6
561.4
682.2
803.0
Mass (m/z)
N
H
O

(D)

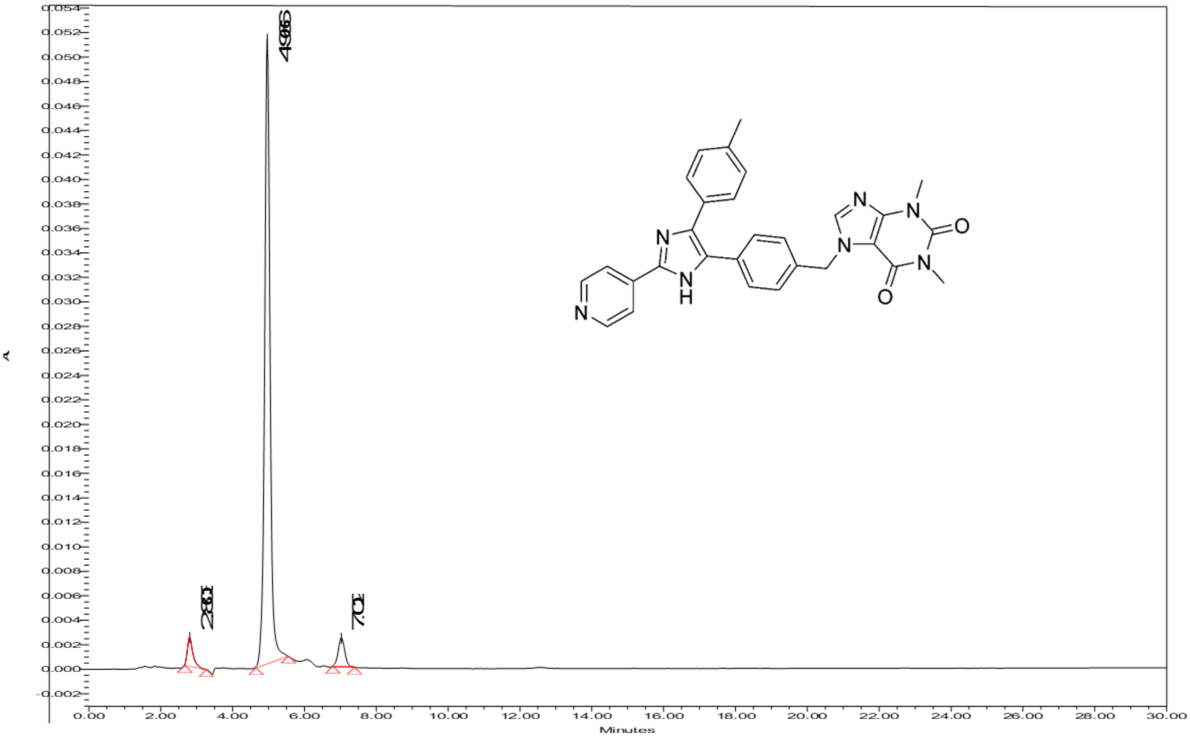

Peak#	Retention Time (min)	Area (µV*sec)	% Area
1	2.803	25531	4.34
2	4.966	533442	90.60
3	7.029	29811	5.06

Fig S17. (A) ^{1}H NMR (400 MHz, CHLOROFORM-*d*) spectra of SG-144C, **(B)** ^{13}C NMR (100MHz, CHLOROFORM-*d*) of SG-144C, **(C)** HRMS (EI+) Spectra of SG-144C. **(D)** HPLC chromatogram of SG-144C.

(A)

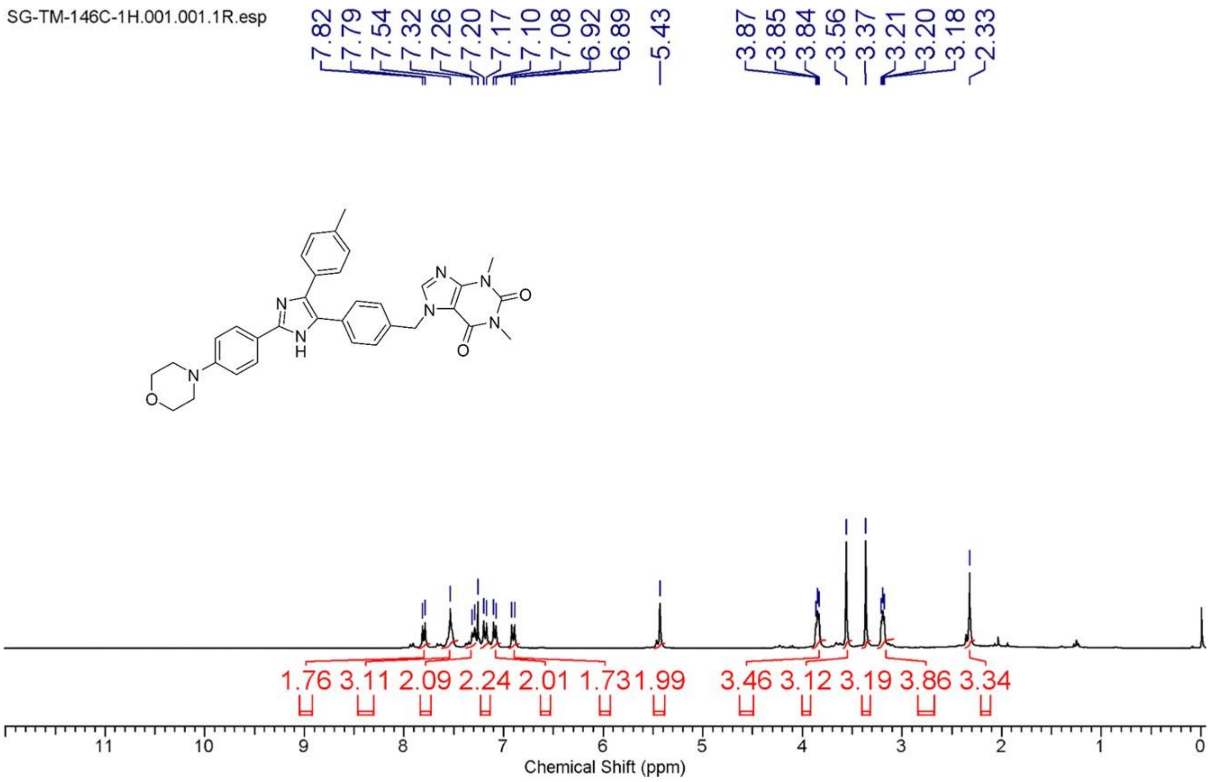
SG-TM-146C-1H.001.001.1R.esp
7.82
7.79
7.54
7.32
7.26
7.20
7.17
7.10
7.08
6.92
6.89
5.43
3.87
3.85
3.84
3.56
3.37
3.21
3.20
3.18
2.33
1.76 3.11 2.09 2.24 2.01 1.73 1.99 3.46 3.12 3.19 3.86 3.34
11 10 9 8 7 6 5 4 3 2 1 0
Chemical Shift (ppm)

(B)

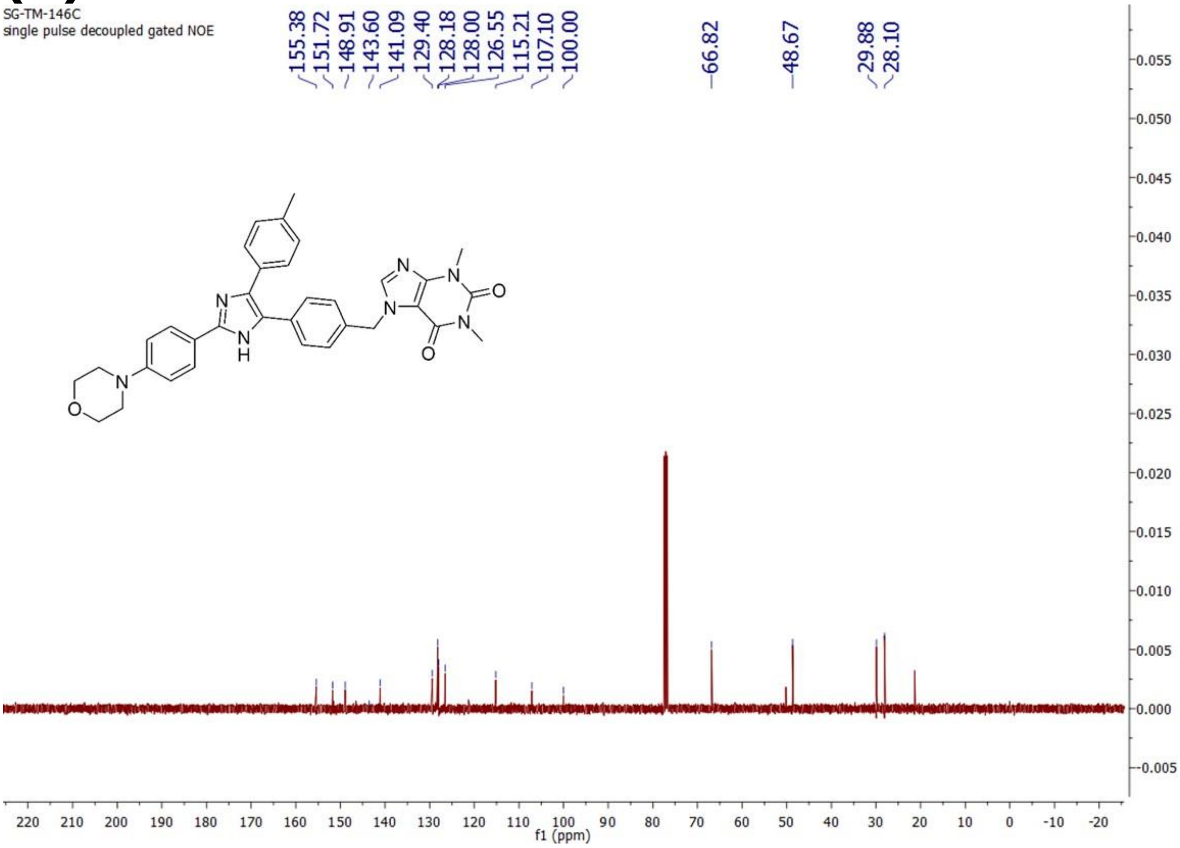
SG-TM-146C
single pulse decoupled gated NOE
155.38
151.72
148.91
143.60
141.09
129.40
128.18
128.00
126.55
115.21
107.10
100.00
66.82
48.67
29.88
28.10
f1 (ppm)

(C)

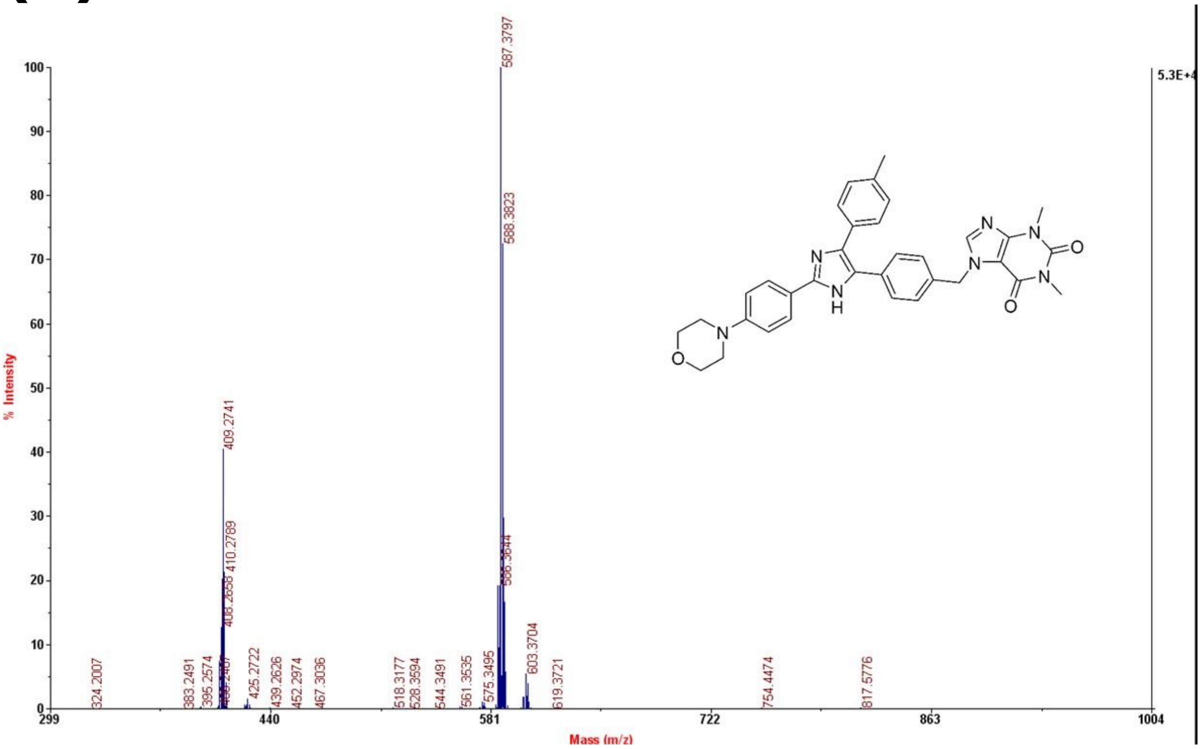
% Intensity
Mass (m/z)
5.3E+4
587.3797
588.3823
409.2741
410.2789
408.2658
586.3644
603.3704
324.2007
383.2491
395.2574
425.2722
439.2626
452.2974
467.3036
518.3177
528.3694
544.3491
561.3535
575.3495
619.3721
754.4474
817.5776
299
440
581
722
863
1004

(D)

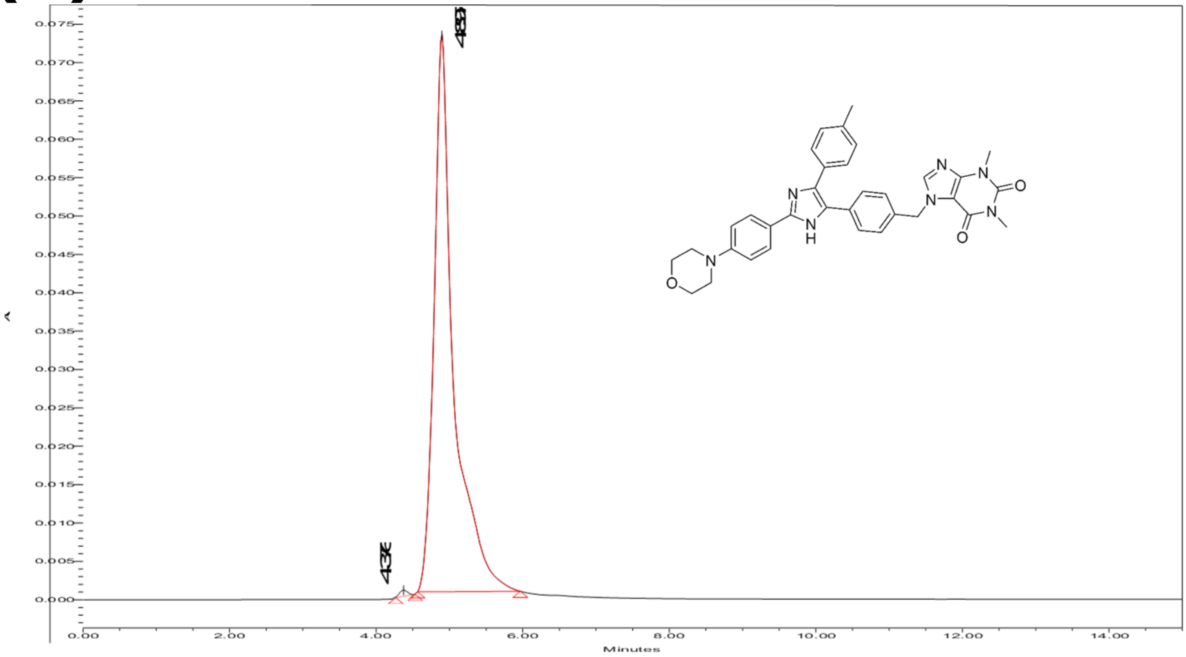

Peak#	Retention Time (min)	Area (µV*sec)	% Area
1	4.376	23308	1.64
2	4.897	213588	98.36

Fig S18. (A) ^{1}H NMR (300 MHz, CHLOROFORM-*d*) spectra of SG-146C, **(B)** ^{13}C NMR (100MHz, CHLOROFORM-*d*) of SG-146C, **(C)** HRMS (EI+) Spectra of SG-146C. **(D)** HPLC chromatogram of SG-146C.

(A)

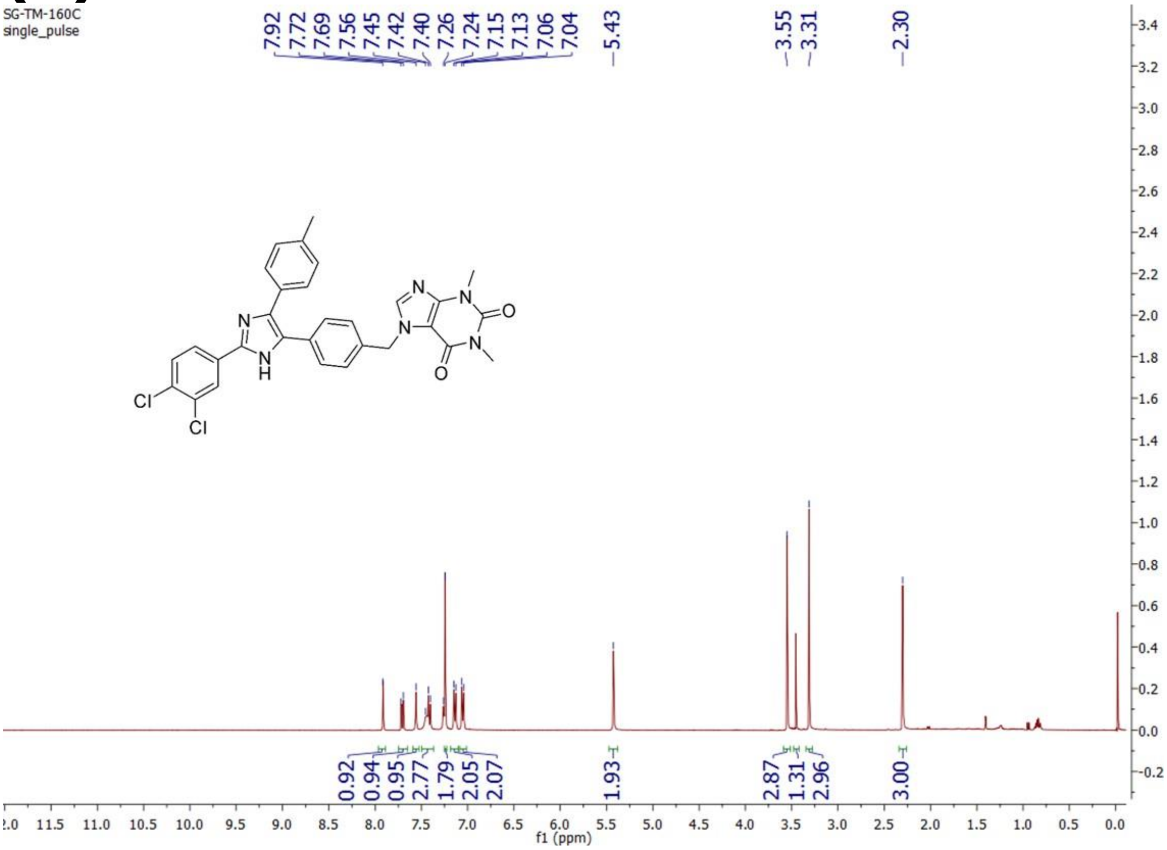
SG-TM-160C
single_pulse
7.92 7.72 7.69 7.56 7.45 7.42 7.40 7.26 7.24 7.15 7.13 7.06 7.04
5.43
3.55 3.31
2.30
0.92 0.94 0.95 2.77 1.79 2.05 2.07
1.93
2.87 1.31 2.96
3.00
N
NH
Cl
Cl
N
N
N
N
O
O
f1 (ppm)

(B)

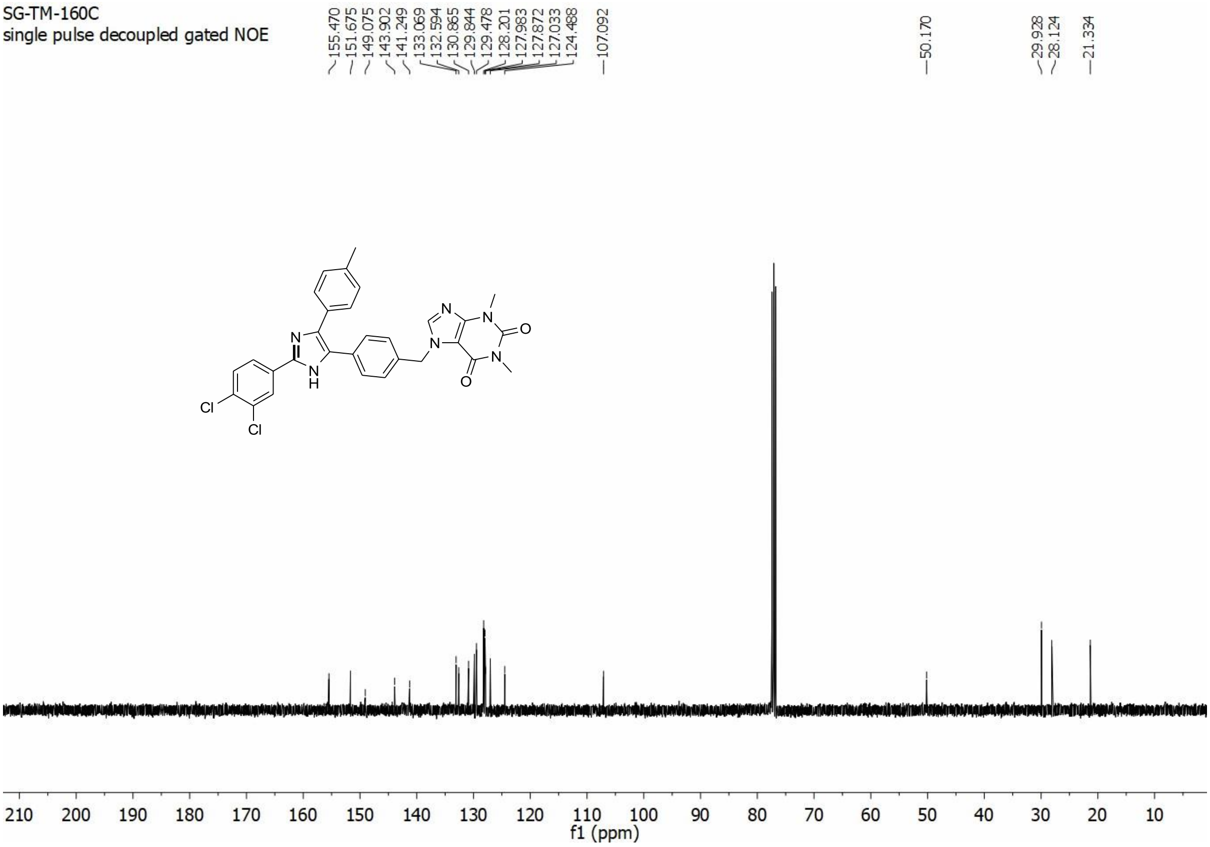
SG-TM-160C
single pulse decoupled gated NOE
155.470 151.675 149.075 143.902 141.249 133.069 132.594 130.865 129.844 129.478 128.201 127.983 127.872 127.033 124.488
107.092
50.170
29.928 28.124
21.334
N
NH
Cl
Cl
N
N
N
N
O
O
f1 (ppm)

(C)

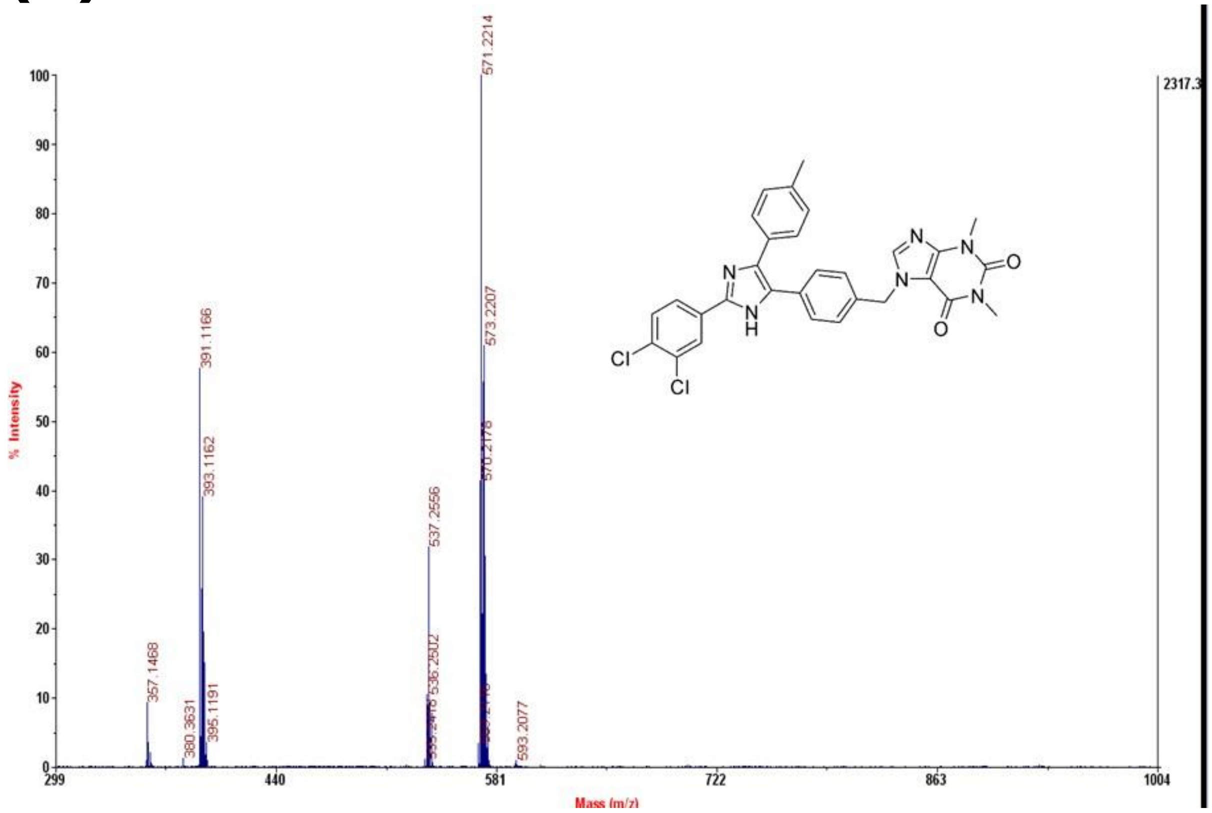
571.2214
2317.3
391.1166
573.2207
393.1162
570.2178
537.2556
536.2502
357.1468
380.3631
395.1191
593.2077
% Intensity
Mass (m/z)
299
440
581
722
863
1004
Cl
Cl
N
H
N
N
N
N
O
O

(D)

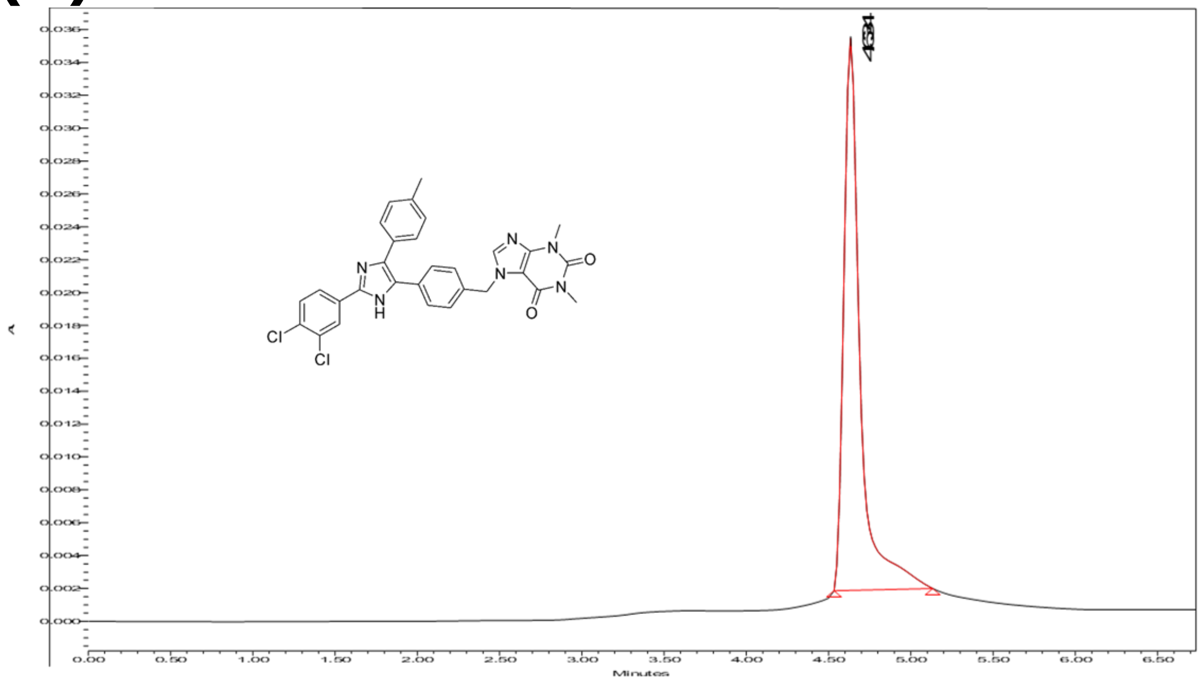

Peak#	Retention Time (min)	Area (µV*sec)	% Area
1	4.634	232783	100

Fig S19. (A) ^{1}H NMR (300 MHz, CHLOROFORM-*d*) spectra of SG-160C, **(B)** ^{13}C NMR (100MHz, CHLOROFORM-*d*) of SG-160C, **(C)** HRMS (EI+) Spectra of SG-160C.**(D)** HPLC chromatogram of SG-160C.

(A)

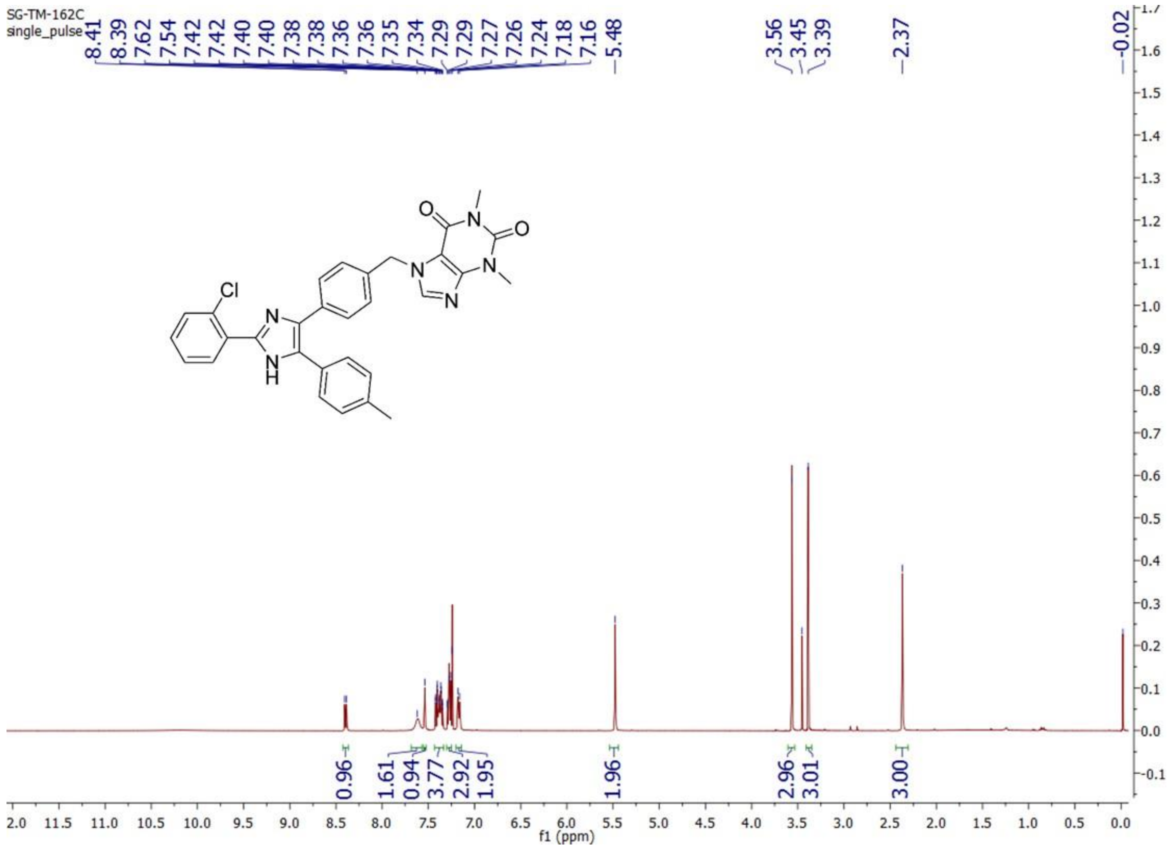
SG-TM-162C
single_pulse
8.41 8.39 7.62 7.54 7.42 7.42 7.40 7.40 7.38 7.38 7.36 7.36 7.35 7.34 7.29 7.29 7.27 7.26 7.24 7.18 7.16 5.48
3.56 3.45 3.39
2.37
-0.02
0.96 1.61 0.94 3.77 2.92 1.95 1.96 2.96 3.01 3.00
f1 (ppm)
Cl
N
H
O
N

(B)

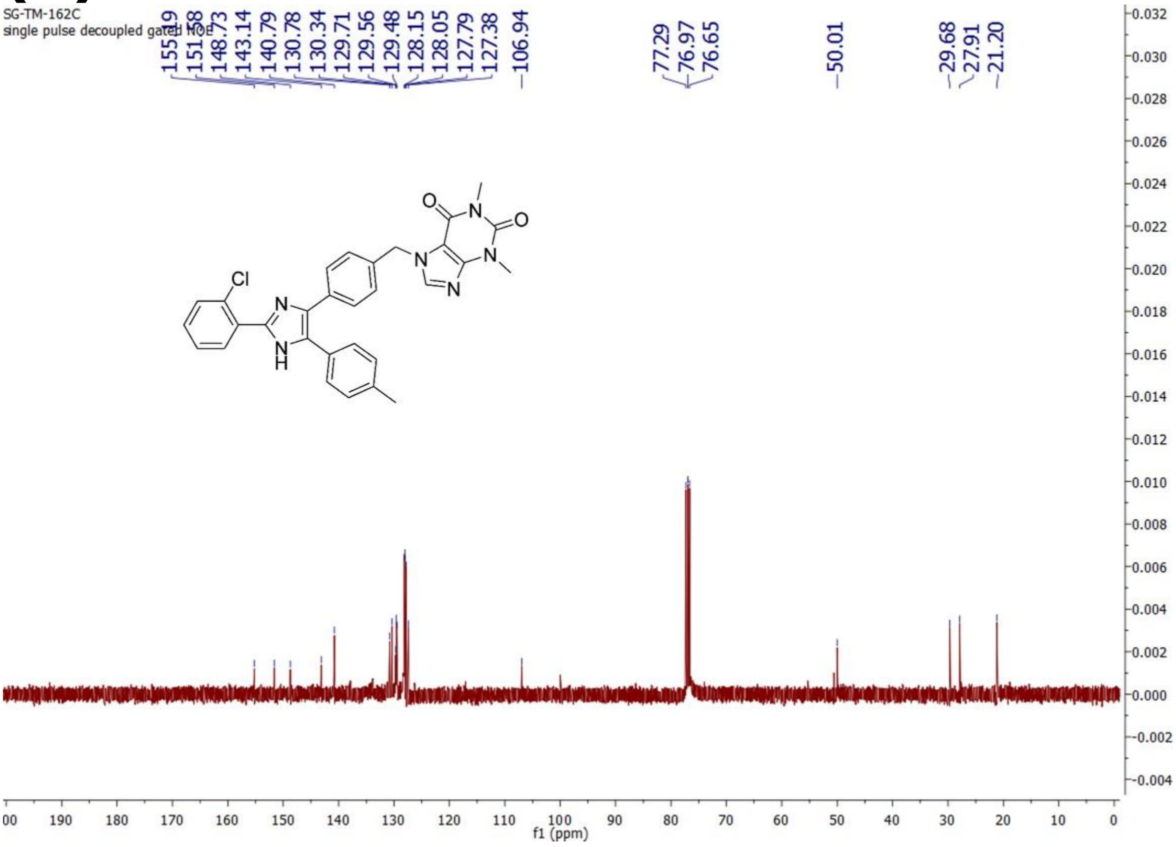

SG-TM-162C
single pulse decoupled gated NOE
155.19 151.58 148.73 143.14 140.79 130.78 130.34 129.71 129.56 129.48 128.15 128.05 127.79 127.38 106.94
77.29 76.97 76.65
50.01
29.68 27.91 21.20
Cl
N
NH
H
O
f1 (ppm)

(C)

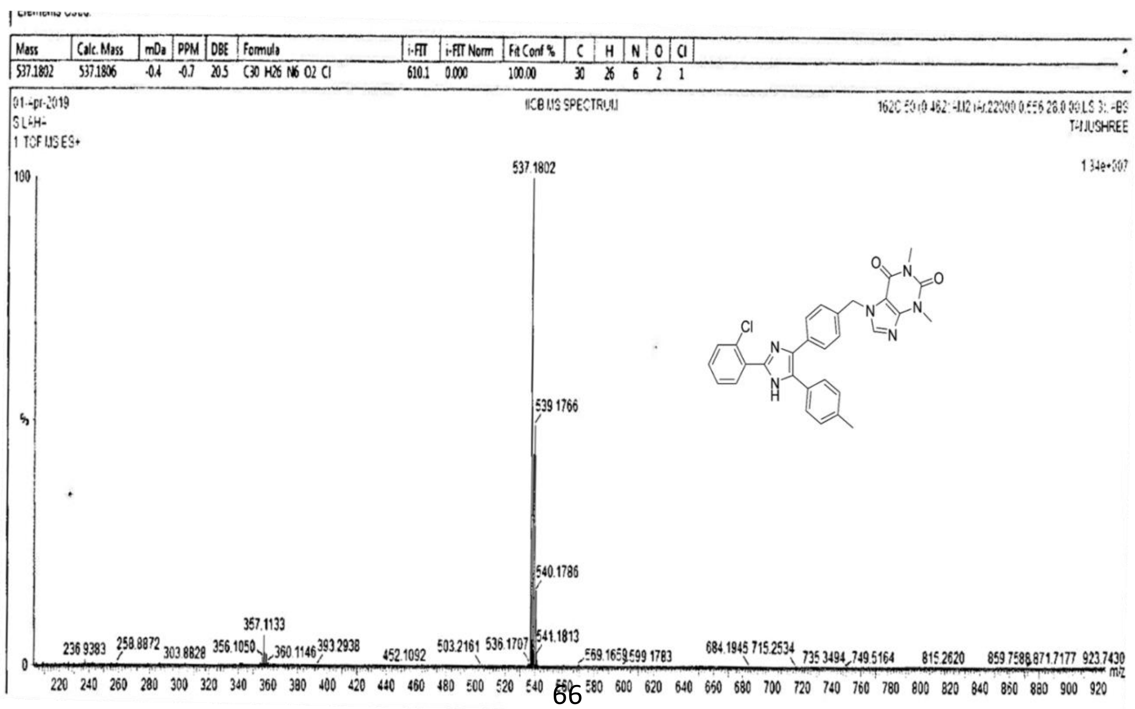

Mass | Calc. Mass | mDa | PPM | DBE | Formula | i-FIT | i-FIT Norm | Fit Conf % | C | H | N | O | Cl
537.1802 | 537.1806 | -0.4 | -0.7 | 20.5 | C30 H26 N6 O2 Cl | 610.1 | 0.000 | 100.00 | 30 | 26 | 6 | 2 | 1
1 TOF MS ES+
537.1802
539.1766
540.1786
541.1813
357.1133
Cl
N
NH
O
m/z

(D)

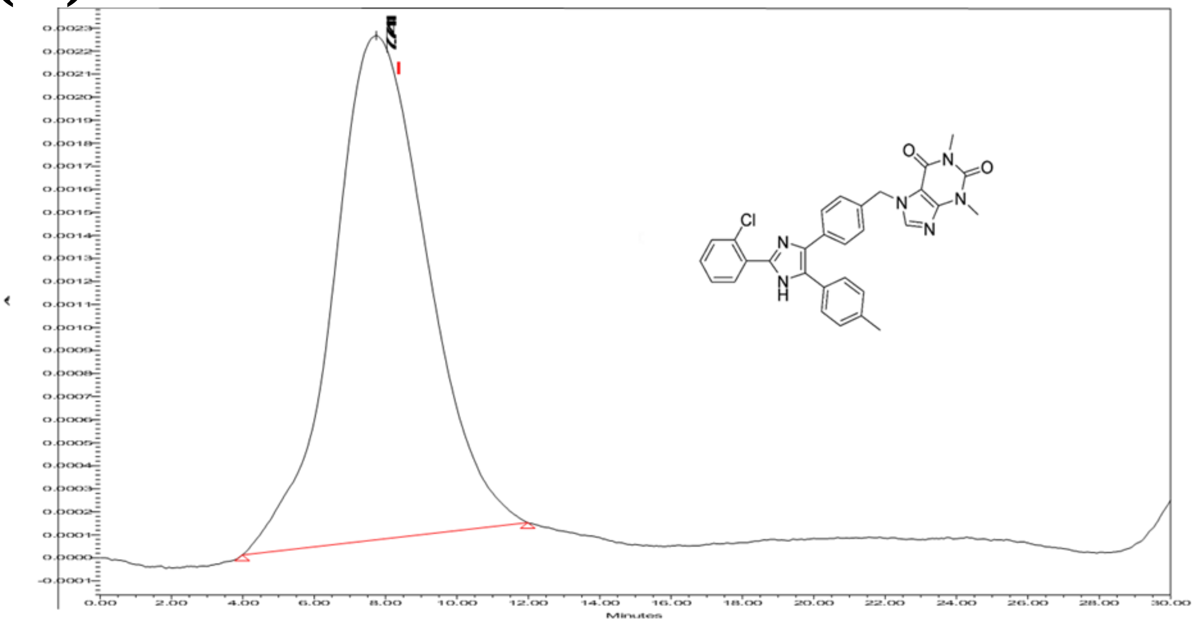

Peak#	Retention Time (min)	Area (µV*sec)	% Area
1	7.741	124327	100

Fig S20. (A) ^{1}H NMR (300 MHz, CHLOROFORM-*d*) spectra of SG-162C, **(B)** ^{13}C NMR (100MHz, CHLOROFORM-*d*) of SG-162C, **(C)** HRMS (EI+) Spectra of SG-162C. **(D)** HPLC chromatogram of SG-162C.

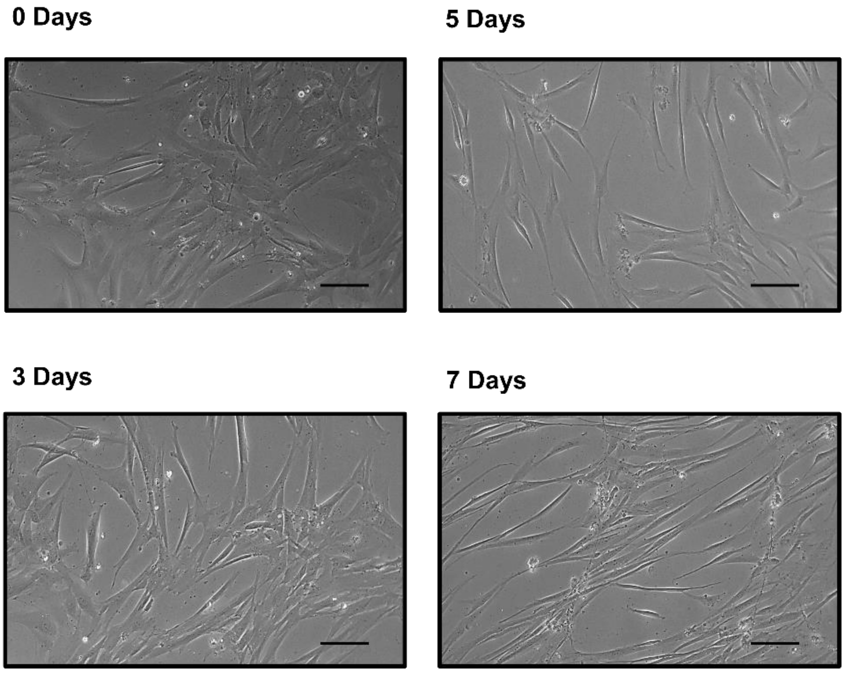

Fig S21. Phase contrast microscopy to check the morphological changes in a day-wise manner after the SG-145C treatment. Prominent extensions and processes were observed till the 7th day of treatment.

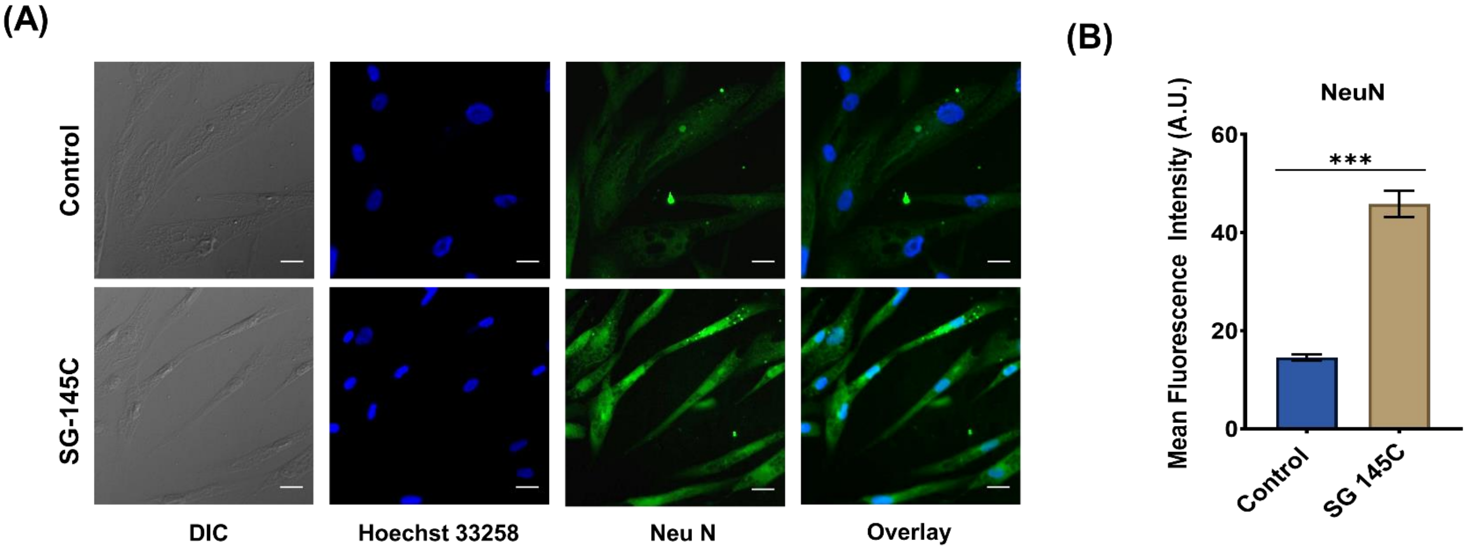

Fig S22.(A) ICC of differentiated neurons revealing the expression of neural markers such as NeuN (AF-488 (B) Mean fluorescence Intensity graph of ICC. Images were captured in 40X magnification where the scale bar corresponds to 20µm. n>3 in all data sets and significance was calculated using students unpaired t-test. *p < .05, **p < .01, ***p < .001

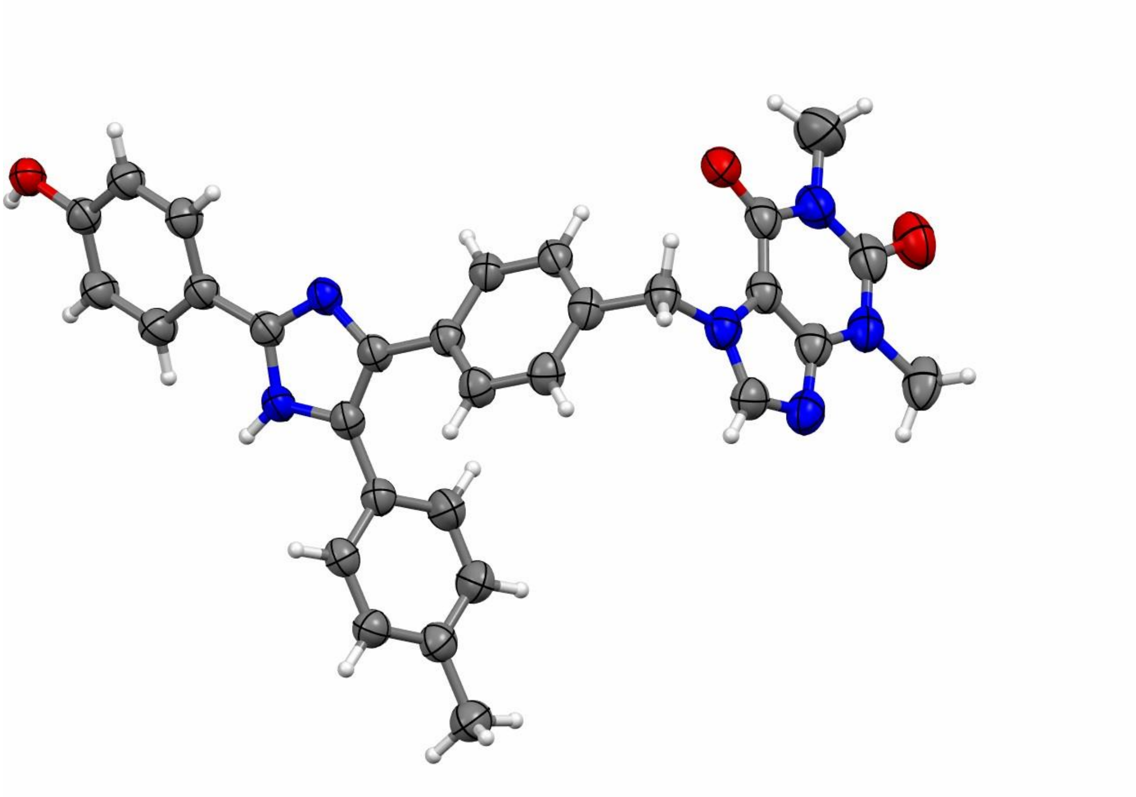

Fig S23. X-ray determined molecular structure of SG-145C. (The thermal ellipsoids are shown in 50% probability level).

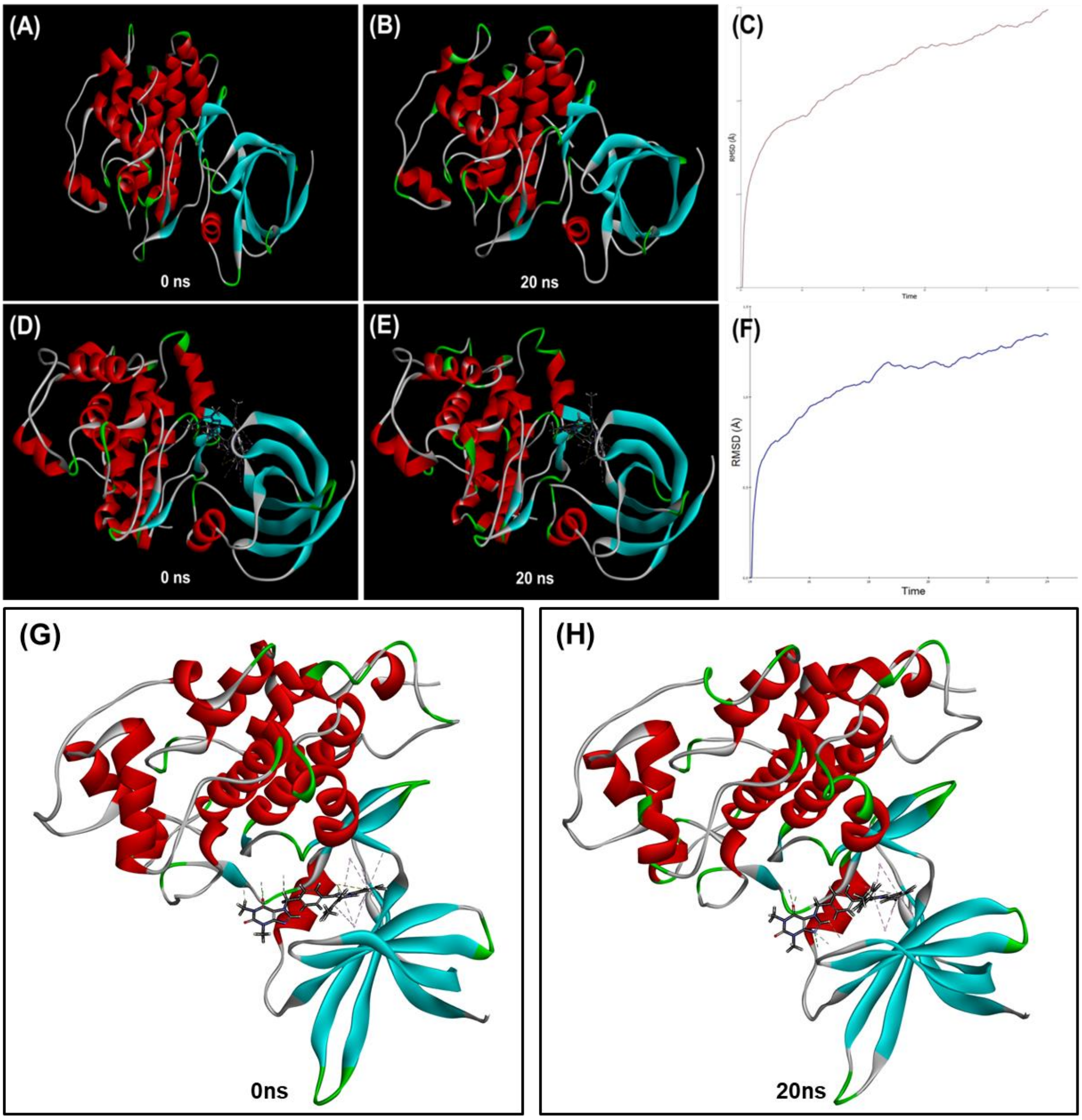

Fig S24. (A, B) MD simulation of 20ns showing the changes in the molecular structure of the lone receptor. **(C)** RMSD calculation of lone receptor. **(D, E)** MD simulation of 20ns showing the changes in the molecular structure of the docked receptor. **(F)** RMSD calculation of lone receptor. **(G,H)** MD simulation of 20ns showing the changes in the molecular structure of the docked receptor after minimizing the energy using CHARMM36 forcefield.

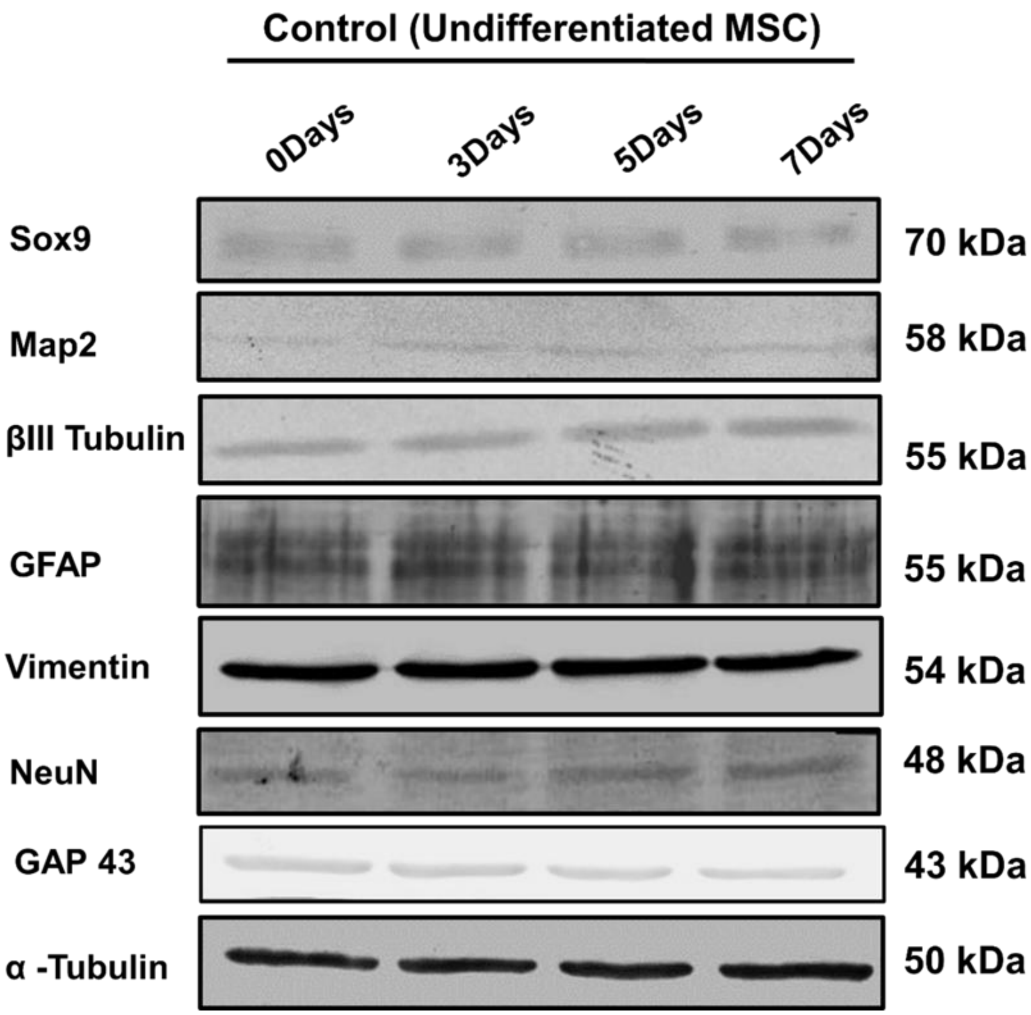

Fig S25 Western Blot to evaluate the neuronal markers in undifferentiated MSCs across various time points in the absence of SG-145C treatment.

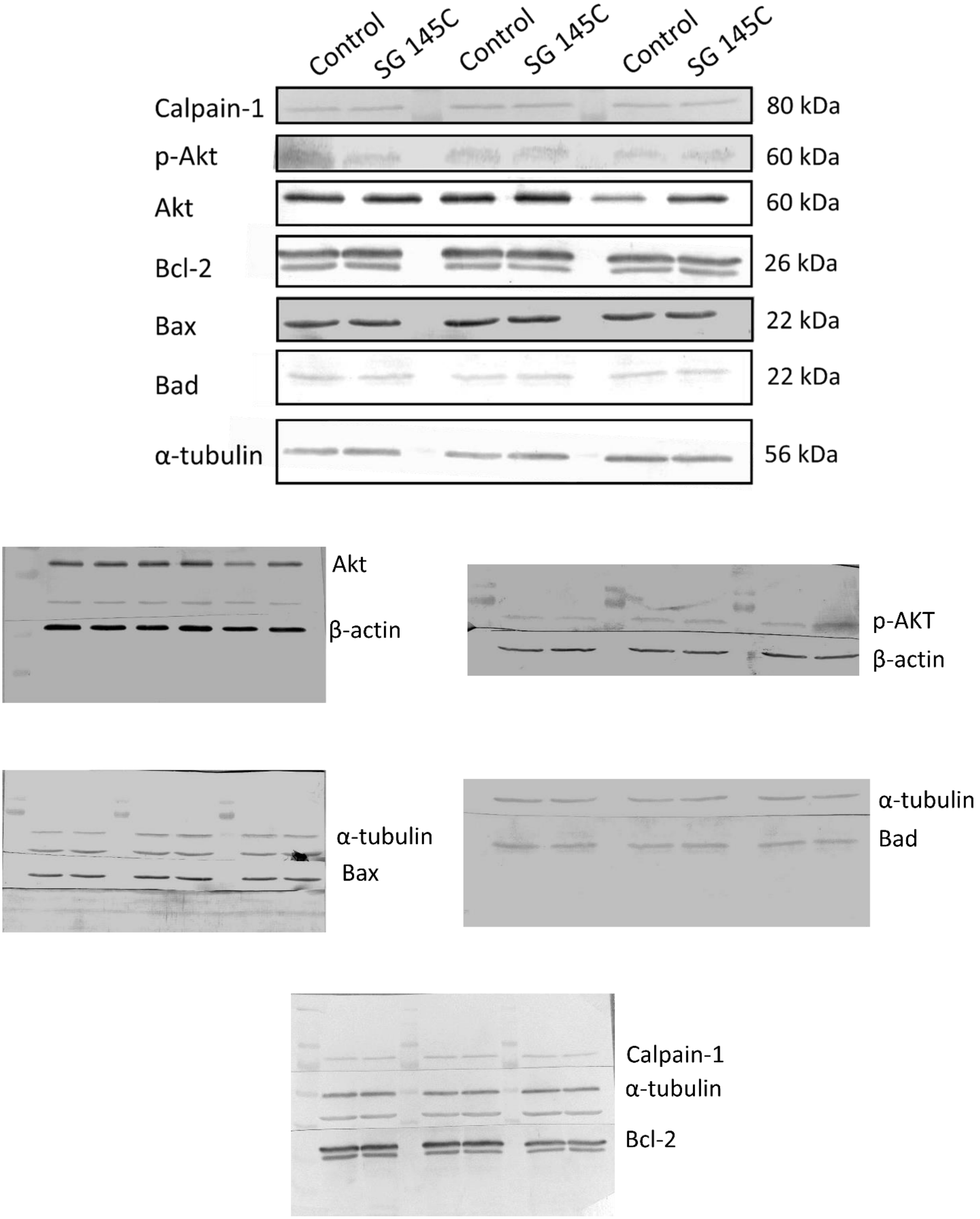

Fig S26. Full Size raw Immunoblots of survival genes in 3 replicates

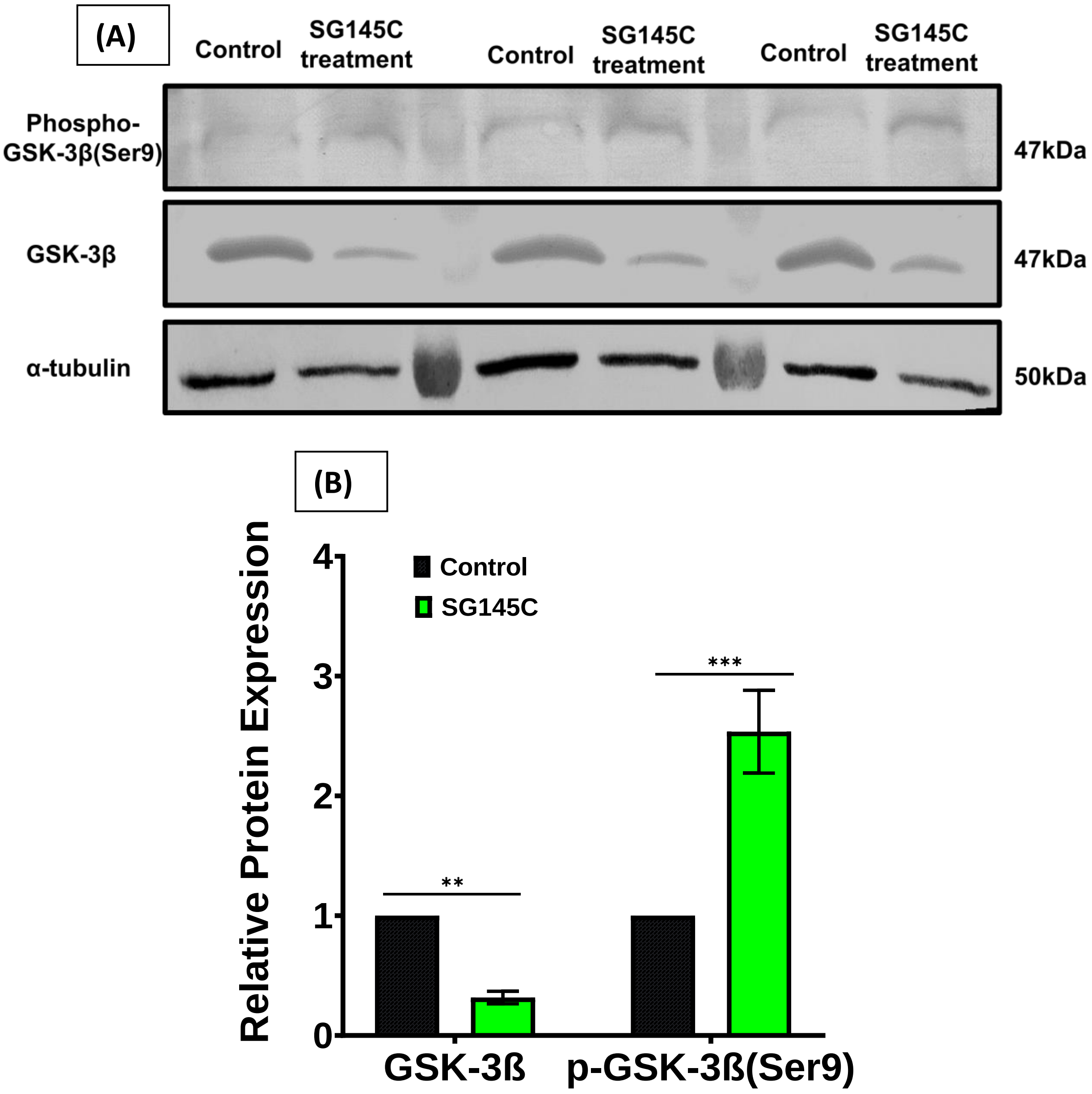

Fig S27. Effect of SG145C on the expression of p-GSK-3β (Ser 9).
(A)Western blot analysis to the check the effect of SG-145C on p-GSK-3β. (B) Densitometric Analysis. Significance was calculated using students unpaired t-test. *$p < .05$, **$p < .01$, ***$p < .001$.

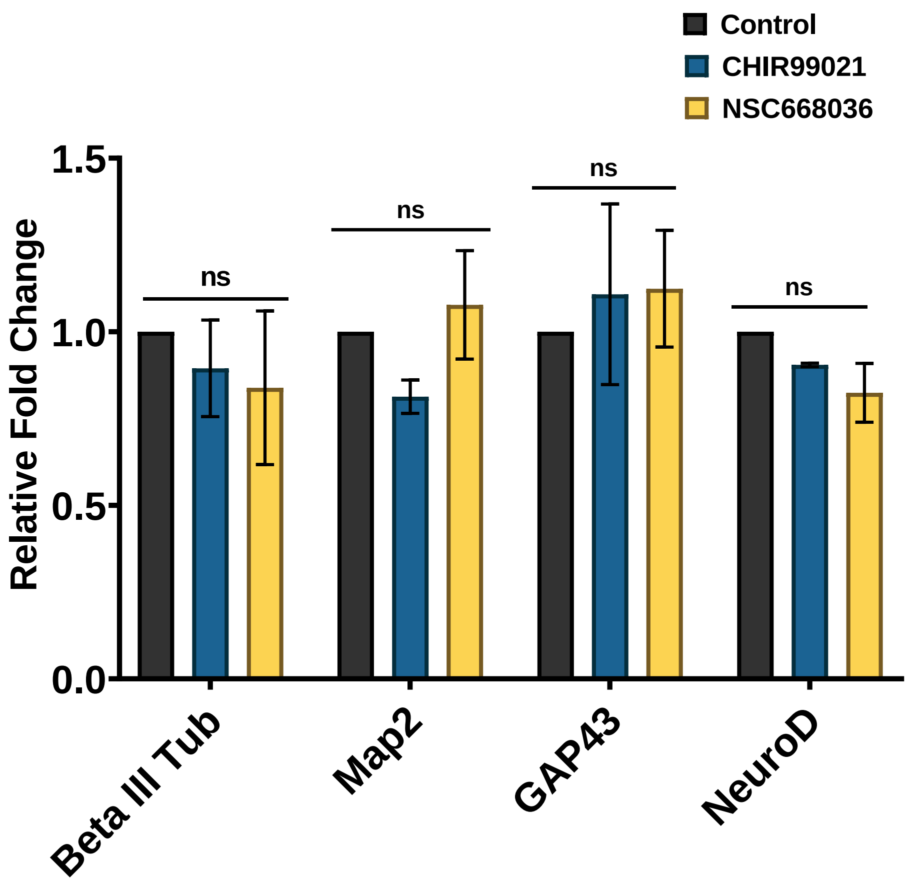

Fig S28. qPCR Analysis to check the effect of CHIR99021 and NSC668036 on neural differentiation. Significance was calculated using students unpaired t-test. *p < .05, **p < .01, ***p < .001.

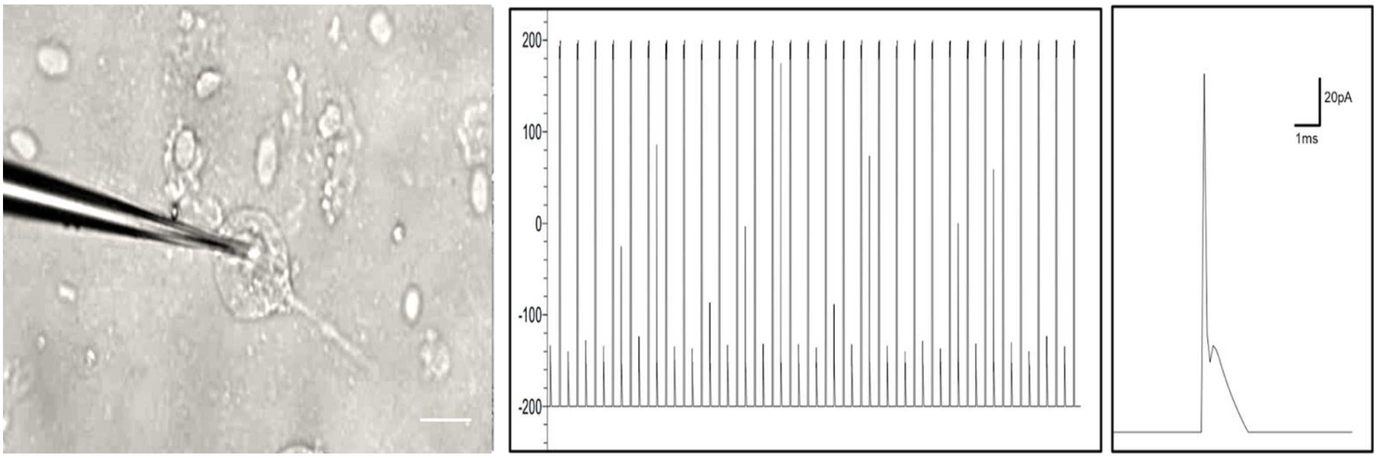

Fig S29. Electrophysiology of transdifferetiated hMSCs showing spikes upon performing voltage clamp.

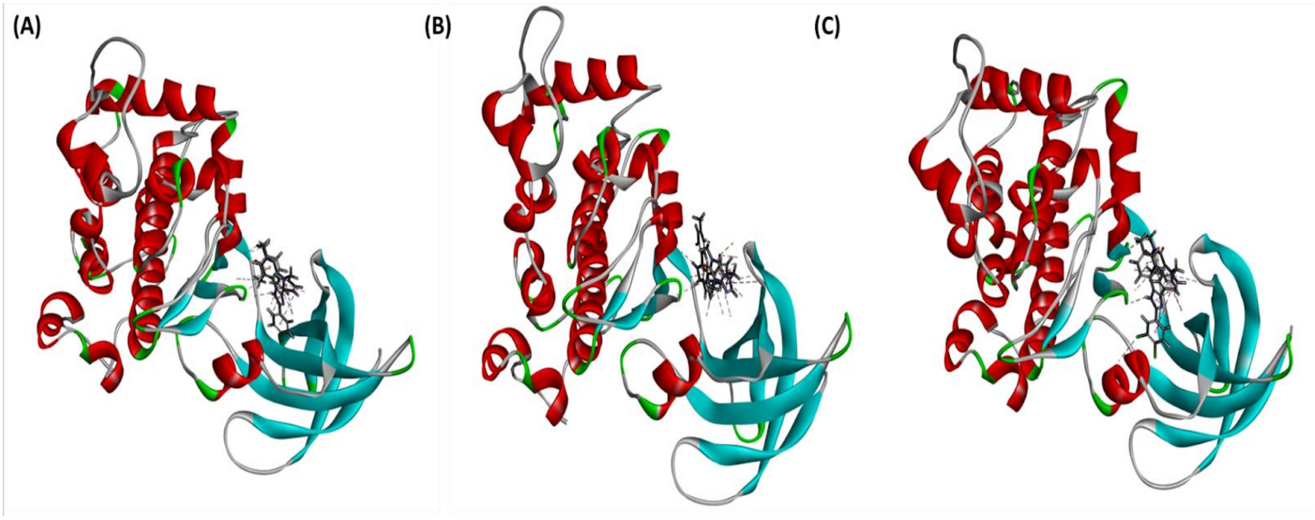

S30. Molecular docking of molecules in CHARMM36 force field.

Reference:

1. Mestres, R. & Palenzuela, J. High atomic yield bromine-less benzylic bromination. *Green Chem.* **2002**, *4*, 314-316.

2. Yu, Y., Liang, Q.; Liu, H., Luo, Z.; Hu, H;, Perlmutter, J.S. & Tu, Z. Development of a carbon-11 PET radiotracer for imaging TRPC5 in the brain. *Organic & biomolecular chemistry* **2019**, *17*, 5586-5594.

3. Kim, G.H.; Halder, D.; Park, J.; Namkung, W. & Shin, I. Imidazole-based small molecules that promote neurogenesis in pluripotent cells. *Angew. Chem. Int. Ed. Engl*. **2014** *25*; 9271-9274
